# Acetylation of C/EBPα inhibits its granulopoietic function

**DOI:** 10.1038/ncomms10968

**Published:** 2016-03-23

**Authors:** Deepak Bararia, Hui Si Kwok, Robert S. Welner, Akihiko Numata, Menyhárt B. Sárosi, Henry Yang, Sheena Wee, Sebastian Tschuri, Debleena Ray, Oliver Weigert, Elena Levantini, Alexander K. Ebralidze, Jayantha Gunaratne, Daniel G. Tenen

**Affiliations:** 1 Cancer Science Institute, National University of Singapore, Singapore 117599, Singapore; 2 Dana Farber/Harvard Cancer Center, Boston, Massachusetts 02215, USA; 3 Department of Internal Medicine III, University Hospital of the Ludwig-Maximilians-University Munich, Munich D-81377, Germany; 4 Experimentelle Leukämie-und Lymphomforschung (ELLF), LMU, Munich D-81377, Germany; 5 German Cancer Consortium (DKTK), Heidelberg D-69120, Germany; 6 German Cancer Research Center (DKFZ), Heidelberg D-69120, Germany; 7 National University of Singapore Graduate School for Integrative Sciences and Engineering, Singapore 117456, Singapore; 8 Harvard Stem Cell Institute, Harvard Medical School, Boston, Massachusetts 02215, USA; 9 University of Alabama at Birmingham, Department of Medicine, Division of Hematology/Oncology, Birmingham, Alabama 35294, USA; 10 Institute of Inorganic Chemistry, Faculty of Chemistry and Mineralogy, Universität Leipzig, Leipzig D-04103, Germany; 11 Translational Biomedical Proteomics Laboratory, Institute of Molecular and Cell Biology, Agency for Science, Technology and Research, Singapore 138673, Singapore; 12 Program in Cancer and Stem Cell Biology, Duke-NUS Graduate Medical School, Singapore 169857, Singapore; 13 Institute of Biomedical Technologies, National Research Council (CNR), Pisa 56124, Italy; 14 Department of Anatomy, Yong Loo Lin School of Medicine, National University of Singapore, Singapore 117594, Singapore

## Abstract

CCAAT/enhancer-binding protein alpha (C/EBPα) is an essential transcription factor for myeloid lineage commitment. Here we demonstrate that acetylation of C/EBPα at lysine residues K298 and K302, mediated at least in part by general control non-derepressible 5 (GCN5), impairs C/EBPα DNA-binding ability and modulates C/EBPα transcriptional activity. Acetylated C/EBPα is enriched in human myeloid leukaemia cell lines and acute myeloid leukaemia (AML) samples, and downregulated upon granulocyte-colony stimulating factor (G-CSF)- mediated granulocytic differentiation of 32Dcl3 cells. C/EBPα mutants that mimic acetylation failed to induce granulocytic differentiation in C/EBPα-dependent assays, in both cell lines and in primary hematopoietic cells. Our data uncover GCN5 as a negative regulator of C/EBPα and demonstrate the importance of C/EBPα acetylation in myeloid differentiation.

## Abstract (web summary)

C/EBPα is an essential transcription factor for myeloid lineage commitment. Here, the authors show that acetylation of C/EBPα at K298 and K302, mediated at least in part by GCN5, impairs C/EBPα DNA binding ability and modulates C/EBPα transcriptional activity.

## Graphical abstract

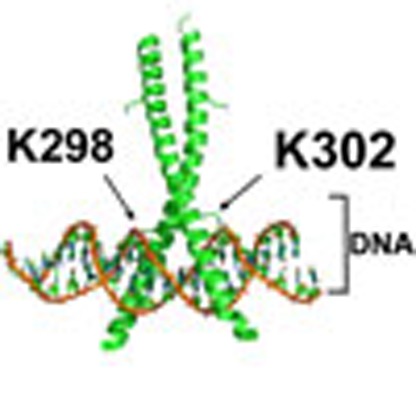

Hematopoiesis is a precisely controlled process involving differentiation of multipotential hematopoietic stem cells (HSCs) into specific lineages, regulated by transcription factors. CCAAT/enhancer-binding protein alpha (C/EBPα) is one of the transcription factors that is crucial for both myeloid differentiation and maintenance of quiescence in adult HSCs. The role of C/EBPα in granulopoiesis is demonstrated through *Mx1-Cre*-driven conditional disruption of C/EBPα in adult mice, resulting in a differentiation block during the transition from common myeloid progenitors to granulocyte monocyte progenitors and increased HSC self-renewal^1^,^2^. Moreover, expression of C/EBPα in both leukaemic cell lines and in human CD34^+^-enriched HSCs lead to granulocytic differentiation^3^,^4^,^5^. Changes in C/EBPα expression and function are associated with impaired myeloid lineage fate decisions while dysregulation of C/EBPα function is frequently associated with leukaemogenesis^6^,^7^,^8^,^9^,^10^. Acetylation catalysed by lysine acetyltransferases (KATs) is an important post-translational modification (PTM), and KATs play pivotal roles in numerous cellular processes. More importantly, histones are not the only physiological acetylation targets^11^. C/EBPα involvement with KATs has been previously reported^12^,^13^,^14^,^15^, but the role of acetylation in modulating C/EBPα function has not been addressed.

In this study, we identify the ability of KAT GCN5 to interact and acetylate C/EBPα on at least lysine K298 and K302 in the basic DNA-binding domain (DBD), which results in impaired DNA-binding activity. Moreover, wild-type (WT) GCN5 but not the mutant, catalytically inactive GCN5 represses C/EBPα transactivation and short hairpin RNA (shRNA) knockdown of GCN5 results in upregulation of C/EBPα activity. C/EBPα acetylation is detectable in myeloid cell lines, primary leukaemic samples and drops dramatically during granulocytic differentiation of 32Dcl3 cells on granulocyte-colony stimulating factor (G-CSF) treatment. An acetylation mimetic mutant of C/EBPα shows loss of DNA binding resulting in the loss of transcriptional activity as predicted by molecular dynamics (MD) simulations. Our study is the first to provide an understanding of how C/EBPα activity is regulated by KAT at the post-translational level.

## Results

### C/EBPα is acetylated in its C-terminal region by GCN5

**Figure 1:**
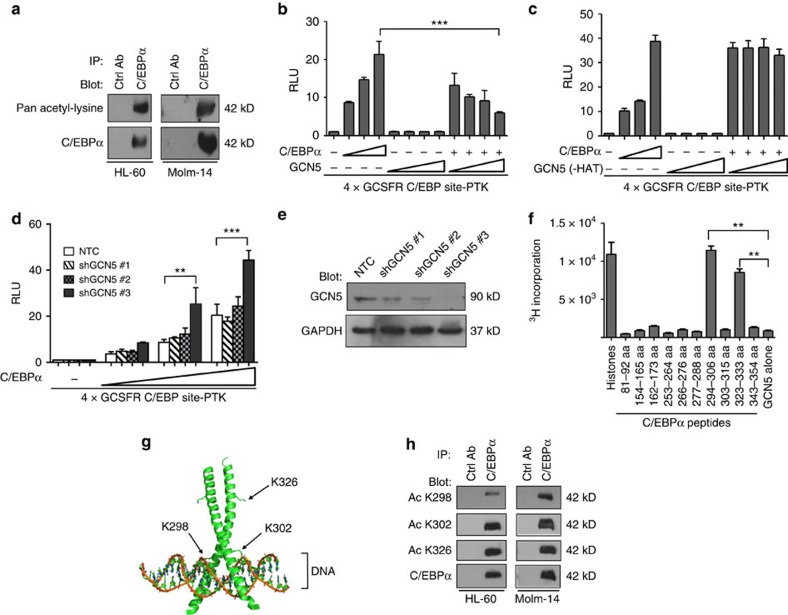
GCN5-mediated acetylation of C/EBPα is linked to loss of transcriptional activity. (**a**) Western blot analysis with anti-pan-acetyl-lysine antibody following immunoprecipitation (IP) of C/EBPα with rabbit anti-C/EBPα in HL-60 and Molm-14 human myeloid cell lysates. Rabbit anti-GFP antibody was used as IP control. (**b**,**c**) GCN5 decreases the ability of C/EBPα to transactivate a minimal p(CEBP)_4_TK promoter in a dose-dependent manner and is dependent on the GCN5 histone acetyltransferase (HAT) domain. 293T cells were transiently transfected with p(CEBP)_4_TK, pRL-null, and pcDNA6 expression plasmids for C/EBPα and with GCN5 or GCN5 (-HAT), respectively. Protein expression of corresponding constructs were shown in Supplementary Fig. 1c. Luciferase activity was measured in duplicate for each experiment and data are shown as mean±s.d. (*N*=3). (**d**) Knockdown of endogenous GCN5 results in an increase in C/EBPα transactivation potential. GCN5 knockdown in 293T cells enhanced C/EBPα transactivation capacity on a minimal p(CEBP)_4_TK promoter with pRL-null and pcDNA6 C/EBPα plasmids. Firefly luciferase readings were normalized against internal control renilla luciferase. Luciferase activity was measured in duplicate for each experiment and data are shown as mean±s.d. (*N*=3). (**e**) Western blot demonstrating knockdown efficiencies of endogenous GCN5 by 3 different shRNAs in 293T cells used in **d**. (**f**) GCN5 acetylates C/EBPα at K298, K302 and K326. C/EBPα peptides were incubated with the GST-HAT domain of GCN5 in the presence of ^3^H-labeled acetyl-CoA. Tritium incorporation by C/EBPα peptides was measured by scintillation counting. Error bars represent mean±s.e.m. Experiments were performed twice in duplicate. (**g**) Structure of the C/EBPα basic region-leucine zipper domain bound to DNA. Arrows indicate the location of the acetylated lysine residues. (**h**) Endogenous C/EBPα is acetylated in the basic leucine zipper region in HL-60 and Molm-14 cells. C/EBPα protein was immunoprecipitated using a rabbit antibody recognizing total C/EBPα protein, followed by immunoblotting against acetylated C/EBPα antibodies (K298, K302, and K326). Rabbit anti-GFP antibody was used as a control for IP. **P<0.01 and ***P<0.001; Student's unpaired *t*-test (**b**,**d** and **f**).

Given C/EBPα expression normally triggers differentiation of immature myeloblasts into mature granulocytes, we investigated why some leukaemic cell lines remain undifferentiated despite high levels of expression of non-mutated C/EBPα. One possibility is the inactivation of WT C/EBPα protein by PTM. Therefore, we investigated whether acetylation is a negative regulator of C/EBPα activity. Using a pan-acetyl-lysine antibody, acetylated C/EBPα was detected in leukaemic cell lines HL-60 and Molm-14 (Fig. 1a). Next, we sought to investigate which acetyltransferase/s represses C/EBPα activity. A C/EBPα-dependent promoter-luciferase reporter construct was used to measure the transcriptional activity of C/EBPα. Co-transfection of various acetyltransferases with a C/EBPα expression plasmid was performed in 293T cells lacking endogenous C/EBPα. Among the acetyltransferases tested, GCN5 was found to repress C/EBPα transcriptional activity (Fig. 1b). Co-transfection of p300 and C/EBPα resulted in an increase in the C/EBPα transactivation activity as previously reported (Supplementary Fig. 1a)^14^. Co-transfection of GCN5 antagonized the ability of C/EBPα to activate the reporter plasmid in a dose-dependent manner, without affecting C/EBPα protein levels (Fig. 1b, Supplementary Fig. 1a–c). GCN5 alone had no effect on luciferase activity, indicating that GCN5 specifically mediates downregulation of the reporter through interaction with C/EBPα (Fig. 1b). Expression of these acetyltransferases and C/EBPα were confirmed by using western blot (Supplementary Fig. 1b). A co-immunoprecipitation experiment showed both GCN5 and PCAF (p300/CBP associated factor, a GCN5 homologue) interact with C/EBPα (Supplementary Fig. 1d). To address the question whether acetyltransferase activity of GCN5 is required to repress C/EBPα transcriptional activity, an acetyltransferase-defective mutation of GCN5 (-HAT, Y621A/P622A) was used in place of WT GCN5 (ref. 16). Indeed, the mutated HAT-deficient form failed to repress C/EBPα transcriptional activity, indicating that acetyltransferase activity is essential for repression (Fig. 1c, Supplementary Fig. 1c). In a reciprocal experiment, C/EBPα transcriptional activity doubled when endogenous GCN5 was efficiently knocked down (shGCN5 # 3; Fig. 1d,e). Taken together, our data indicate that GCN5 inhibits C/EBPα transactivation in an acetyltransferase-dependent manner, although the role of other HATs in this process cannot be excluded.

Subsequently, we performed an *in vitro* acetyltransferase assay using GCN5 and C/EBPα peptides. K298, K302 and K326 were identified as the sites of acetylation by GCN5 (Fig. 1f, Supplementary Fig. 1e–g and Supplementary Table 1). These lysine residues have high degree of evolutionary conservation across different species, suggesting crucial role for C/EBPα function (Supplementary Fig. 1h). K298 and K302 are exposed on the basic DBD, whereas K326 resides in the leucine zipper dimerization domain (Fig. 1g)^17^,^18^.

To further investigate the protein domains involved in the C/EBPα–GCN5 interaction, we performed co-immunoprecipitation assays in 293T cells (Supplementary Fig. 1i). While immunoblot analysis using FLAG and V5 antibodies revealed that GCN5 interacts with C/EBPα WT, C/EBPα 1-207, and C/EBPα p30/120-358, it failed to interact with C/EBPα 204-358 (Supplementary Fig. 1j). By performing pull-down assays with FLAG antibody for C/EBPα-TAD1 (Transactivation domain 1), and C/EBPα-DBD separately, we were unable to detect any interaction between GCN5 and TAD1 or DBD domain of C/EBPα (Supplementary Fig. 1k). Collectively, these observations suggest that the GCN5 interaction domain in C/EBPα lies in the N-terminal region of C/EBPα (Supplementary Fig. 1l).

The relevant lysine residues (K298, K302 and K326) were substituted with arginine to generate non-acetylated mimetic forms of C/EBPα (referred to as K3R). We further tested whether a pan-acetyl antibody is able to detect acetylation differences between C/EBPα WT and K3R or C/EBPα-DBD and C/EBPα-DBD K3R (Supplementary Fig. 1m). Immunoprecipitated C/EBPα WT or K3R mutant showed no difference in acetylation using a pan-acetyl antibody, both with (lanes 4 and 5) and without (lanes 2 and 3) GCN5 co-transfection. In addition, co-transfection with DBD or DBD K3R did not show any acetylation signal using a pan-acetyl antibody (lanes 6 and 7). Immunoprecipitated WT, K3R, DBD, and DBD K3R were detected by using V5 antibody. These results are in accordance with our domain-mapping data, suggesting that the C/EBPα DBD domain does not interact with GCN5, and therefore no acetylation signal is observed from either DBD or DBD K3R when co-transfected with GCN5 (Supplementary Fig. 1l).

To detect acetylation of C/EBPα in cells at K298, K302 and K326, site-specific anti-acetyl-C/EBPα antibodies were generated using synthetically acetylated peptides. The acetylated and non-acetylated forms of these peptides were first confirmed by mass spectrometry. Our antibodies were able to readily recognize acetylated C/EBPα at K298, K302 and K326. When a non-acetylated mimetic form of C/EBPα, that is, K3R was used, no signal was detected, confirming that the antibodies we generated are capable of specifically detecting acetylated C/EBPα (Supplementary Fig. 1n). Consistently, western blotting with these site-specific acetylation antibodies showed an increase in acetylated C/EBPα when GCN5 and C/EBPα were co-expressed in 293T cells (Supplementary Fig. 1o). We also examined whether K298, K302 and K326 were acetylated in HL-60 and Molm-14, and the results are consistent when probed with site-specific antibodies (Fig. 1h). These data indicate that our acetylation-specific antibodies were able to detect C/EBPα acetylation in the DBD of C/EBPα.

### Loss of C/EBPα acetylation on myeloid differentiation

We looked at whether endogenous C/EBPα is acetylated at K298, 302 and 326 *in vivo* and if the acetylation status of C/EBPα changes with respect to myeloid differentiation. Within the hematopoietic system, expression of C/EBPα is detectable in early myeloid precursors and its expression is necessary and sufficient for neutrophilic differentiation^5^,^19^,^20^. We used non-leukaemic 32Dcl3 cells to assess C/EBPα acetylation status on differentiation. Murine 32Dcl3 cells are dependent on interleukin-3 (IL-3) for survival and proliferation, and readily differentiate into mature granulocytes on removal of IL-3 and addition of G-CSF^21^,^22^.

**Figure 2:**
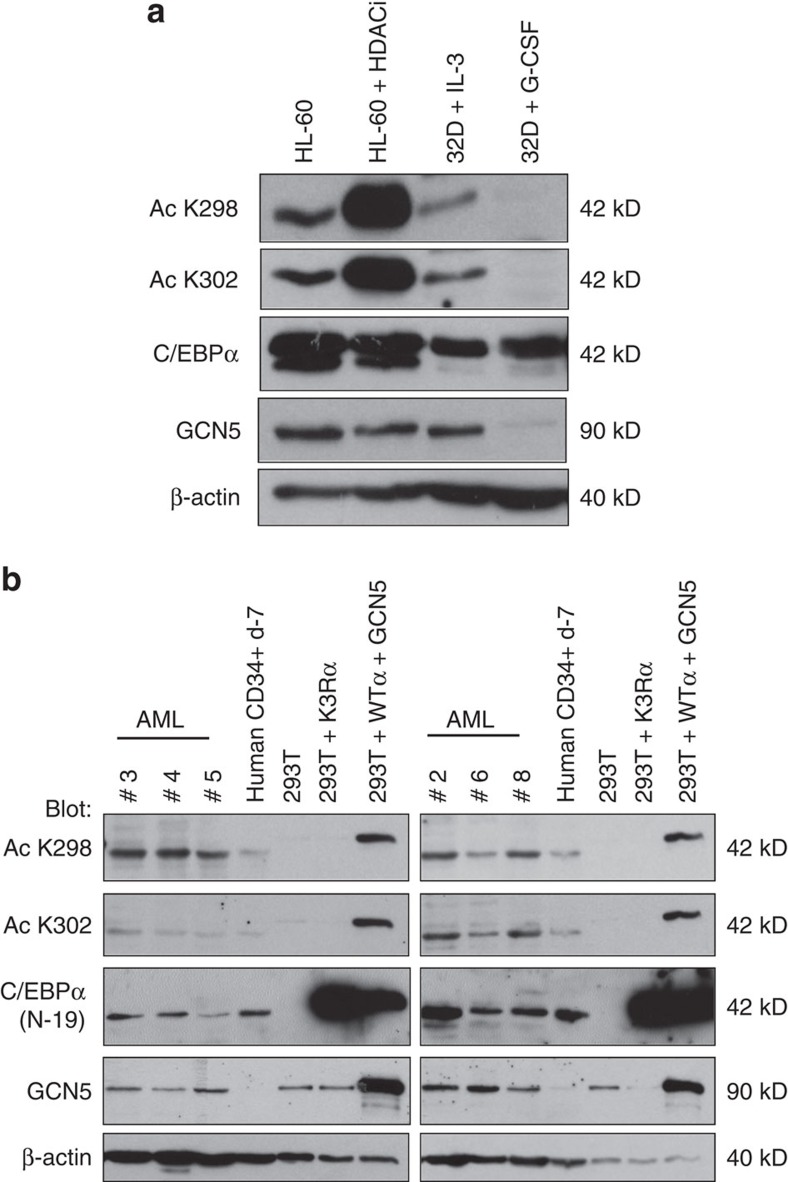
Loss of C/EBPα acetylation on granulocytic differentiation and detection in primary AML samples. (**a**) 32Dcl3 murine IL-3 dependent cells are differentiated into granulocytes with addition of G-CSF for 4 days. Western blot analysis showed nuclear extracts using anti acetyl K298, K302, C/EBPα (D56F10), GCN5, and β-actin antibodies. HL-60 cells and HL-60 treated with histone deacetylase inhibitors (HDACi: 400 nM Trichostatin acid (TSA), 20 mM nicotinamide (NA) and 5 mM sodium butyrate (NB) for 12-16 h before harvest.) were used as a positive control for C/EBPα specific acetylated antibodies. (**b**) Enrichment of C/EBPα acetylation at K298 and K302 in human AML cells compared to partially differentiated (day 7) human CD34^+^ cells. Whole-cell lysates were prepared and blotted with anti-acetyl K298, K302, C/EBPα, GCN5, and β-actin antibodies, respectively. Non-acetylated mimetic C/EBPα K3R was used as a negative control while C/EBPα co-transfected with GCN5 was used as a positive control for acetylation at the respective residues.

FACS and Giemsa-stained cytospins analysis revealed an efficiency of ≥80% in granulocytic differentiation of 32Dcl3 cells on day 4 on G-CSF induction (Supplementary Fig. 2a). Western blot results showed enrichment of acetylated C/EBPα for K298 and K302 in 32Dcl3 cells before differentiation by G-CSF is initiated (Fig. 2a). On G-CSF induction, there was a reduction in acetylation levels for K298 and K302, which coincides with the loss of GCN5 protein expression. We then used acetylation-specific antibodies to determine the endogenous levels of C/EBPα acetylation in primary acute myeloid leukaemia (AML) samples in which C/EBPα levels could be detected by western blot. Undifferentiated human CD34^+^ cells expressed such low levels of C/EBPα protein that we were unable to compare their C/EBPα acetylation signal with AML. Therefore, we used G-CSF treated CD34^+^ cells on day 7, in which C/EBPα protein expression was detected by western blot. C/EBPα was acetylated at K298 and K302, and GCN5 was higher in AML samples than in partially differentiated human CD34^+^ cells (day 7; Fig. 2b, Supplementary Fig. 2c). We did not observe a stronger acetylated signal at K302 in AML samples #3, #4 and #5 as compared with other AML samples (Fig. 2b). We were also unable to detect any signals using acetylated K326-specific antibody in AML samples and in G-CSF-induced differentiated 32Dcl3 cells (Supplementary Fig. 2b). Our AML sample size was insufficient to distinguish the difference between K302 acetylation, and lack of K326 acetylation (Supplementary Table 2). Our data indicate loss of C/EBPα acetylation at K298 and K302 on G-CSF-mediated differentiation of murine 32Dcl3 cells. In addition, C/EBPα acetylation is easily detectable in AML patients compared with partially differentiated human CD34^+^ cells. Interestingly, GCN5 is expressed at higher levels in AML patients than in normal CD34^+^ cells (Supplementary Fig. 2d).

### Predicting loss of DNA binding for acetylated C/EBPα

**Figure 3:**
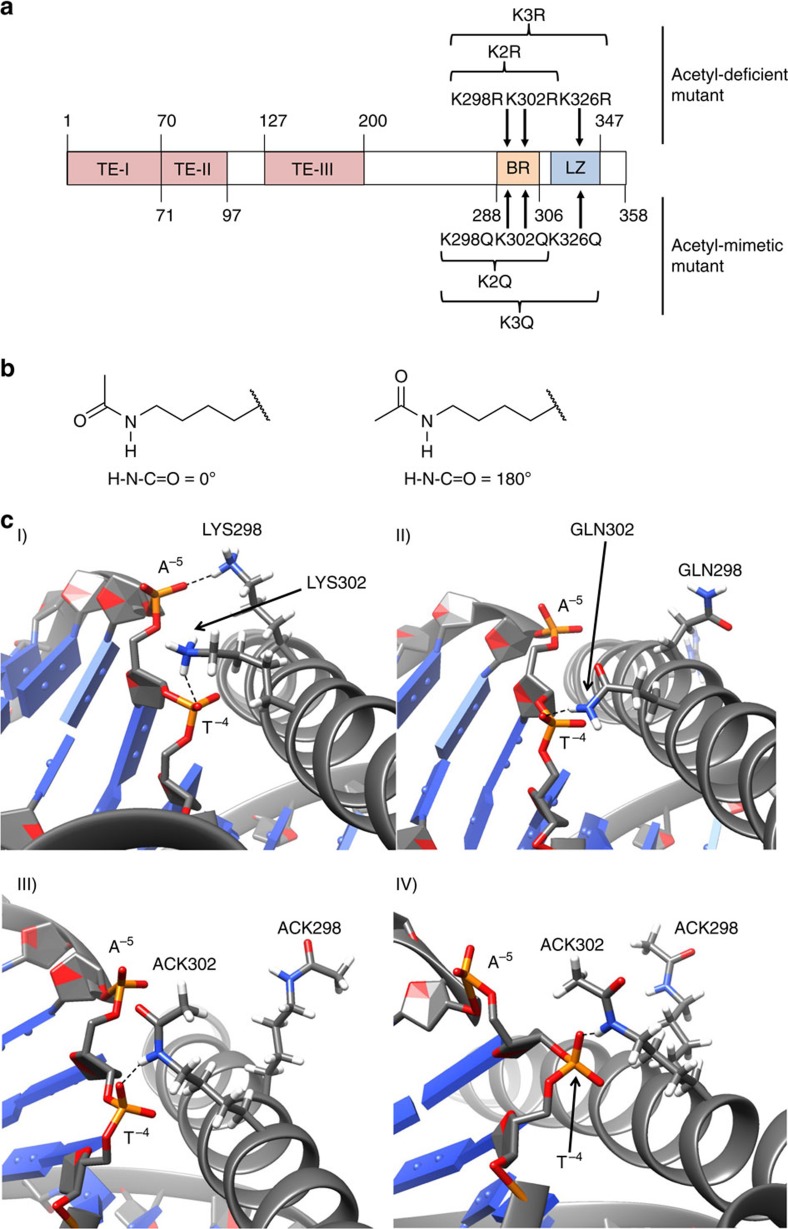
Molecular dynamics simulation of acetylation at K298 and K302. (**a**) Diagram of the C/EBPα protein. The positions of the three transactivation elements (TEs), basic region-leucine zipper (BR-LZ) and putative acetylation sites for GCN5 are indicated. (**b**) The two conformations of the acetylated lysine (ACK) side chain built into the starting coordinates of the acetylated models and preserved during the MD simulations. (**c**) MD snapshots during 3 ns simulation showing selected protein–DNA interactions: I) C/EBPα WT-DNA; II) K2Q-DNA; III) K2Ac_a-DNA; and IV) K2Ac_b-DNA. Hydrogen bonds are shown as dashed lines.

On acetylation, lysine side chains are changed in that the acetyl group neutralizes the positive charge. Substitution of lysine with glutamine (neutral side chain) mimics the acetylated form^11^. To examine whether glutamine substitution was consistent with lysine acetylation and to examine the impact of C/EBPα acetylation on its structure (Fig. 3a), we conducted MD simulations on four model systems. In addition to the WT DNA and acetylation mimic (K2Q-DNA) systems, two acetylated models were also included in this study. The H-N-C=O dihedral angle of the acetylated lysine residues was set to 0° and 180° in the two acetylated models (hereafter termed as K2Ac_a-DNA and K2Ac_b-DNA, respectively, Fig. 3b; see Computational methods section for details). The backbone atoms of the four models showed similar fluctuations during MD simulations (Supplementary Fig. 3). As compared with the C/EBPα WT–DNA complex, lysine acetylation (K2Ac_a-DNA and K2Ac_b-DNA) and acetylation mimic (K2Q-DNA) raised the calculated protein–DNA-binding free energies substantially (Table 1). This indicates a decrease in DNA-binding potency of the models (K2Ac_a-DNA, K2Ac_b-DNA and K2Q-DNA). According to the crystal structure of C/EBPα WT DNA, one of the key interactions is formed between K298 and A^−5^ (ref. 18). During MD simulations, the three ammonium hydrogen atoms of K302 formed hydrogen bonds with the phosphate oxygen atoms of T^−4^ (Fig. 3c, Supplementary Table 3). The electrostatic interaction energy between K298 and K302, and DNA residues T^−4^ and A^−5^ is a major component of the total protein–DNA-binding energy. Both acetylated lysine and glutamine formed significantly less hydrogen bonds with DNA during the 3 ns MD simulations (Supplementary Table 3). Furthermore, the electrostatic stabilization between the C/EBPα protein and DNA residues were significantly lowered in the mutant systems (Supplementary Table 4). Acetylation might also destabilize the C/EBPα structure. 15 μs MD simulations have shown higher conformational fluctuations and loss of enzymatic activity both during acetylation and mutation of K104 to glutamine in RAS^23^. Similar structural destabilization might also be responsible for the loss of C/EBPα transcriptional activity on acetylation.

### Acetylation mimetic mutants impairs myeloid differentiation

To investigate the consequence/s of acetylation on C/EBPα function, we generated and tested single and various combinations of K298, K302 and K326 sites in which lysine was replaced with either arginine (hereafter termed as non-acetylated mimetic) or glutamine (acetylation mimetic) in C/EBPα-dependent reporter assays. Single acetylation mimetics of all three residues did not have any major effect on C/EBPα transactivation (Supplementary Fig. 4a). Interestingly, K298 and K302Q (C/EBPα-K2Q) and K298, K302 and K326Q (C/EBPα-K3Q) acetylation mimetic mutation combinations completely abolished C/EBPα transactivation (Supplementary Fig. 4a,b). Western blots of the C/EBPα mutations confirmed similar expression levels, indicating that the loss in reporter activity is not due to reduction in C/EBPα protein levels (Supplementary Fig. 4a,b). All corresponding lysine-to-arginine mutations had luciferase activity similar to that of C/EBPα-WT, substantiating that mimicking acetylation results in the inactivation of C/EBPα function. To eliminate the possibility of altered conformation and affected transactivation by clusters of lysine-to-glutamine mutations, another mutated form (C/EBPα-KIIIQ) was generated to mimic acetylation of a cluster of three lysine residues (K275, K277 and K278) located close to the DBD. These three lysine residues were substituted with glutamine. Although both mutants represented acetylation mimetic forms, unlike the C/EBPα-K3Q form, C/EBPα-KIIIQ indeed retained transcriptional activity (Supplementary Fig. 4b). This strongly argues that retaining the positive charge is only crucial for the residues in the basic region, and substituting lysine residues for glutamine does not always result in loss of function.

**Figure 4:**
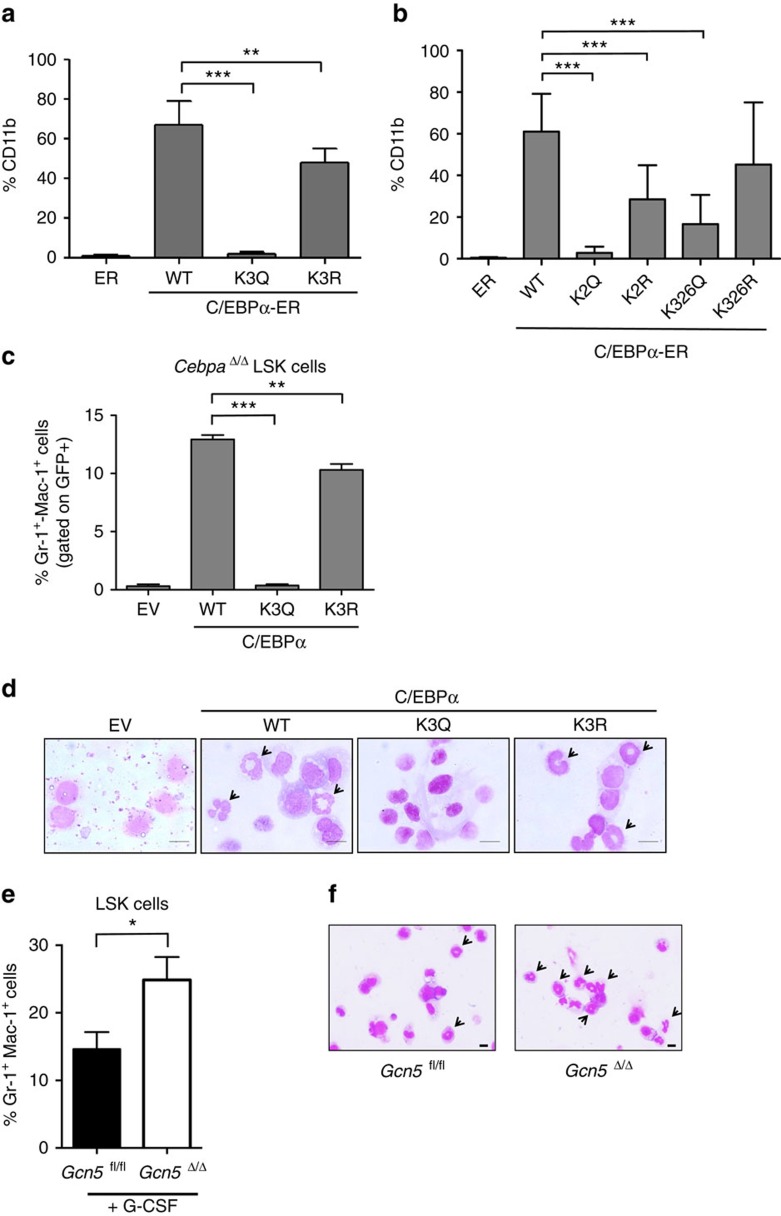
Loss of differentiation potential in acetylation mimetic mutations of C/EBPα. (**a**,**b**) C/EBPα K3Q-ER and C/EBPα K2Q-ER fail to upregulate differentiation marker CD11b in K562 cells. K562 cells were stably transfected with EV-ER, C/EBPα WT-ER, K3Q-ER, K3R-ER, K2R-ER, K2Q-ER, K326R-ER, or K326Q-ER. Induction of C/EBPα nuclear localization was performed with addition of 5 μM β-estradiol. Cells were analysed with FACS for surface marker CD11b following 4 days of culture. Bar graph shows the percentage of CD11b^+^ cells, which is indicative of granulocytic differentiation. Data are mean±s.d. from two independent clones for each construct. ***P< 0.001; Student's unpaired *t*-test *N*=6 (**a**) and *N*=9 (**b**). (**c**) C/EBPα-K3Q fails to differentiate hematopoietic early progenitor cells. Lin^−^Sca-1^+^c-Kit^+^ (LSK) cells from bone marrow of *Cebpa*^Δ/Δ^ mice were transduced with retroviral expression vectors pMIG EV, C/EBPα-WT, C/EBPα-K3R, and C/EBPα-K3Q to induce differentiation into granulocytes. Cells gated on the GFP^+^ fraction were analysed on day 7 for surface markers Gr-1 and Mac-1, indicative of granulocytic differentiation. Data are mean±s.d. ***P<0.001; Student's unpaired *t*-test *N*=3. (**d**) Lack of mature granulocytes in C/EBPα-K3Q transduced LSK cells. Cytospins were stained with Wright-Giemsa. Original magnification x100, scale bars indicate 10 μm. The arrows indicate granulocytes with their polymorphonuclear morphology. (**e**) Increase in myeloid cells on deletion of *Gcn5*. LSK cells from *Gcn5*^Δ/Δ^ *Vav-iCre*^+^ and control (*Gcn5*^fl/fl^) mice were cultured in G-CSF supplemented media. After 7 days, viable cells were assessed for relative expression of Gr-1 and Mac-1 using flow cytometry. Data shown are representative from four independent experiments done in triplicate. Data are mean±s.d. *P<0.05; Student's unpaired *t*-test. (**f**) Mature granulocytes morphology from G-CSF induced *in vitro* culture. Cells were subjected to Wright-Giemsa staining and pictures were acquired at an original magnification × 40. Scale bars indicate 10 μm. The arrows indicate granulocytes with their polymorphonuclear morphology.

The C/EBPα-deficient human cell line K562 was engineered to conditionally express β-estradiol-inducible constructs to study how various C/EBPα mutants affect differentiation^4^. Without induction, fusion proteins are constitutively expressed in the cytoplasm, where they are inactive. On induction with β-estradiol, the fusion protein translocates to the nucleus, where it is active. Consistent with results from MD simulation and luciferase assays, cells expressing C/EBPα WT-oestrogen receptor (ER), C/EBPα K3R-ER and C/EBPα K2R-ER differentiated, whereas cells expressing ER only, C/EBPα K3Q-ER and C/EBPα K2Q-ER, remained in an undifferentiated state as assessed by surface expression of the myeloid marker CD11b, cell morphology and neutrophilic enzymatic activity (Fig. 4a,b, Supplementary Fig. 4c–f). Expression levels of the individual fusion protein in K562 stable clones were assessed by western blots (Supplementary Fig. 4g). Furthermore, our results showed that K562 C/EBPα-WT-ER was able to inhibit the increase in cell numbers within 5 days of culture, as compared with vector-alone and vehicle (ethanol) control (Supplementary Fig. 4h). The K562 C/EBPα-K2Q mutant cell line showed an intermediate inhibition in cell number, which lacked statistically significant difference (*P* value=0.148, *N*=6 with two-way analysis of variance test; Supplementary Fig. 4h). C/EBPα WT-ER was able to downregulate endogenous c-Myc as previously reported by our group whereas C/EBPα-K3Q and C/EBPα K2Q-ER mutants showed partial downregulation of c-Myc (Supplementary Fig. 4i)^24^.

Using a murine model, we further explored whether the acetylation mimetic form is unable to differentiate progenitors into mature granulocytic cells. As granulocyte differentiation is impaired in *Cebpa*^fl/fl^ *Mx1-cre*^*+*^ mice, we chose this model to analyse the differentiation potential of the various C/EBPα mutants. Ten- to twelve-week-old *Cebpa*^fl/fl^ *Mx1-cre+* mice and control (*Cebpa*^fl/fl^ *Mx1-cre*^-^) mice were treated with poly(I:C)^1^,^2^,^20^. Bone marrow was harvested 17–21 days later, and excision of the *Cebpa* gene was confirmed by PCR. Multipotent progenitor Lin^−^Sca-1^+^c-Kit^+^ (LSK) cells from *Cebpa*^Δ/Δ^ mice were FACS-purified and cultured. Cells were transduced with retroviruses-expressing green fluorescent protein (pMSCV IRES GFP, MIG empty vector), WT (C/EBPα-WT), or mutated forms of C/EBPα (C/EBPα -K3Q and C/EBPα-K3R). Expression levels of MIG C/EBPα-WT and mutants were assessed by western blot (Supplementary Fig. 4j). After 7 days of culture, the percentage of granulocytic differentiation was measured by Gr-1 and Mac-1 surface staining and by Giemsa stain for morphological analysis (Fig. 4c,d). The C/EBPα-K3Q acetylation mimetic mutation was notably unable to form mature granulocytes. These results suggest that acetylation at K298, K302 and K326 antagonizes C/EBPα granulocytic differentiation potential. In addition, acetylation of K298 and K302 alone is sufficient to impede differentiation.

Further testing of the possibility of GCN5 involvement in myeloid differentiation was done by using 10–12 weeks old *Gcn5*^Δ/Δ^*Vav-iCre*^+^ and control mice (*Gcn5*^fl/fl^)^25^,^26^. LSK cells were FACS-purified and placed in myeloid culture conditions containing G-CSF. LSK cells from *Gcn5* conditional knockout (*Gcn5*^Δ/Δ^) produced a 1.7-fold increase in the percentage of Gr-1^+^ Mac-1^+^ cells compared with control mice (*Gcn5*^fl/fl^; Fig. 4e,f; *P* value=0.0137, *N*=3, with unpaired two-tailed Student's *t*-test). In conclusion, G-CSF induced *in vitro* myeloid differentiation is enhanced in the absence of GCN5.

### Acetylation at K298 and K302 reduces DNA-binding ability

To understand how acetylation of K298 and K302 leads to C/EBPα inactivation, we assessed changes in homodimerization, cellular sublocalization and dominant negative function of the mutant forms over C/EBPα WT. No difference in homodimerization or localization between C/EBPα WT and mutated forms was detected (Supplementary Fig. 5a,b). In a C/EBPα-dependent promoter-luciferase reporter assay, co-transfection of the C/EBPα-K2Q mutant together with C/EBPα WT did not show any dominant negative effect by the K2Q mutant, unlike the C/EBPα p30 form (Supplementary Fig. 5c)^27^. Expression of C/EBPα, C/EBPα p30 form, and C/EBPα-K2Q mutant were confirmed using western blot (Supplementary Fig. 5d).

**Figure 5:**
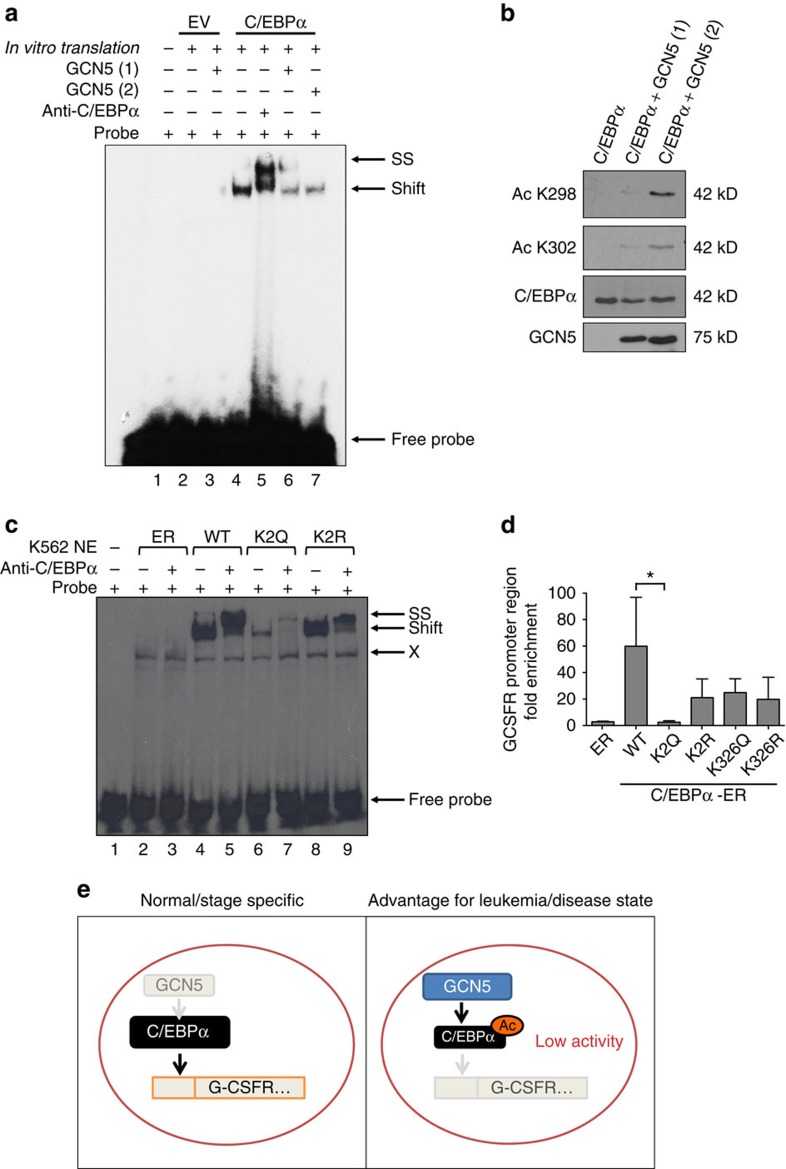
Acetylation in the basic region impairs DNA binding ability. (**a**) GCN5 attenuates DNA binding ability of C/EBPα *in vitro*. EMSA was performed using a double-stranded C/EBPα binding site oligonucleotide. *In vitro*-translated C/EBPα was incubated with two separate sources of recombinant GCN5 (HAT) domain proteins. Co-incubation of *ivt* C/EBPα with GCN5 (lane 6, 7) resulted in decrease in DNA binding as compared to C/EBPα alone (lane 4). No binding was observed from *ivt*. Empty vector (EV) control (lane 2) or from EV incubated with GCN5 (lane 3). Specificity of C/EBPα binding to the probe was shown by supershift (SS) using C/EBPα antibody (lane 5). (**b**) Western blot showing *in vitro*-translated C/EBPα acetylation at K298 and K302 by recombinant GCN5 (HAT domain) protein used in EMSA in **a**. (**c**) C/EBPα acetylation mimetic (K2Q-ER) showed reduced DNA binding affinity in EMSA. Equal amounts of nuclear extracts from K562 lines stably transfected with EV-ER (lanes 2, 3), C/EBPα WT-ER (lanes 4, 5), C/EBPα K2Q-ER (lane 6, 7), and C/EBPα K2R-ER (lane 8, 9) were used. Cells were treated with 5 μM β-estradiol for 45 min. Lane 1 contained probe only. In lanes 3, 5, 7 and 9, 1 μL of a supershifting C/EBPα antibody was added. SS indicates supershifted complex; Shift indicates C/EBPα complex; and X refers to nonspecific complex observed with this probe. The representative experiment out of three is shown here. (**d**) Acetylation mimetic form K2Q was not enriched at a C/EBPα target gene locus G-CSFR^27^. ChIP analysis of stimulated (45 min) K562 EV-ER, C/EBPα WT-ER, K2Q-ER, and K2R-ER cells using ER antibody. Fold enrichment is calculated compared to binding to control gene, the inactive involucrin (IVL) locus. Data are mean±s.d. (*N*=3). *P<0.05; Student's unpaired t-test. *P* value between C/EBPα WT and K2R, K326R, and K326Q is not significant. (**e**) Model hypothesizing the effect of C/EBPα acetylation on its function in normal hematopoiesis and leukaemia. Non-acetylated C/EBPα is capable of inducing granulocytic differentiation. C/EBPα acetylation leads to loss in DNA binding and loss of recruitment to C/EBPα target genes such as the G-CSF receptor, thereby inhibiting differentiation.

To investigate whether acetylation by GCN5 alters C/EBPα DNA binding *in vitro*, we performed electrophoretic mobility shift assays (EMSA). C/EBPα was acetylated by an *in vitro* acetyltransferase assay using GCN5 HAT domain protein. EMSA analysis showed that acetylation of *in vitro*-translated C/EBPα reduces its ability to bind to the C/EBPα binding site (Fig. 5a). No binding was observed in the control lysate. Western blot analysis of the reaction used for EMSA confirmed C/EBPα acetylation at K298 and K302 residues (Fig. 5b). MD simulations predicted a dramatic reduction in the ability of C/EBPα to bind to its target DNA sequences by the acetylation mimetic C/EBPα-K2Q (Fig. 3c, Table 1). The DNA-binding abilities of WT and mutated forms of C/EBPα were also assessed by EMSA, using nuclear extracts from K562 lines expressing ER empty vector, C/EBPα WT-ER, C/EBPα K2Q-ER and C/EBPα K2R-ER. As expected, C/EBPα K2Q-ER extracts demonstrated weakened DNA binding as compared with C/EBPα WT-ER and C/EBPα K2R-ER. Using anti-C/EBPα antibody for supershift, we demonstrated specificity of the C/EBPα-dependent binding in EMSA (Fig. 5c and Supplementary Fig. 5f). EMSA performed using 293T cells transfected with C/EBPα K2Q without the ER-tag showed loss of DNA binding similar to K2Q-ER (Supplementary Fig. 5e,g). The G-CSF receptor is an important downstream target gene of C/EBPα, and its promoter region contains C/EBPα-binding sites^28^. Minimal recruitment of C/EBPα K2Q-ER to the G-CSF receptor promoter by chromatin immunoprecipitation (ChIP) was observed (Fig. 5d). In summary, these results support the hypothesis that acetylation of these two residues leads to the loss of DNA-binding activity and subsequent loss of induction of granulocytic differentiation.

## Discussion

In this study, we demonstrated that acetylation on C/EBPα leads to the loss of differentiation potential. We identified three lysine residues (K298, K302 and K326) as the acetylation target sites in C/EBPα by GCN5. With acetylation site-specific antibodies, we detected acetylation at these three sites in leukaemic cell lines expressing endogenous C/EBPα. We provided evidence that C/EBPα acetylation levels and GCN5 expression are reduced on granulocytic differentiation. Furthermore, we demonstrated C/EBPα acetylation in primary leukaemic samples. Using C/EBPα-dependent murine and cellular models, we demonstrated that the acetylation mimetic forms (K2Q and K3Q) are incapable of driving granulocytic differentiation in inducible C/EBPα-dependent assays and in murine primary cells as a result of loss of DNA binding ability.

This is the first report demonstrating an acetyltransferase that has repressive activity on C/EBPα function, consistent with the reciprocal pattern of expression of GCN5 and C/EBPα in HSCs, in which GCN5 is enriched, while C/EBPα is barely detectable^1^,^29^. Previous studies have described the involvement of multiple PTMs in modulating C/EBPα^30^,^31^,^32^,^33^. In particular, phosphorylation of serine 21 has been reported to be clinically relevant during myeloid differentiation. Hyper-phosphorylation at S21 blocks the maturation of leukaemic blasts and neutrophilic differentiation of CD34^+^ progenitors^9^,^10^. Although both acetylation and phosphorylation impair the ability of C/EBPα to drive differentiation, the mechanisms of inactivation appear to be distinct. Acetylation results in a loss of DNA-binding capacity, while phosphorylation alters conformation of C/EBPα dimers^34^. The differential effects of these PTMs on C/EBPα are attributes to the inherent differences in the chemistry and sites of these modifications.

Our protein domain mapping data for C/EBPα indicates that GCN5 docking interaction occurs at the N terminus, whereas acetylation sites are located at the C terminus in the DBD. Such observations have been reported in C/EBPα and E2F in which deletion of the C/EBPα N terminus or the basic region mutation leads to loss of inhibition of E2F activity^4^. Several studies have reported a number of C/EBPα mutations lacking differentiation potential, some of which are located in the basic region. The BRM2 (I294A, R297A) mutant protein fails to differentiate cells into granulocyte and adipocyte lineages. Unlike C/EBPα-K2Q described in our study, BRM2 does not abrogate DNA binding^17^,^35^. The BR3 (R297G, K298T, R300G, K302N) mutant contains four substitutions in the basic region. Similar to our results in C/EBPα-K2Q mutations, although BR3 is also incapable of binding DNA, protein retains the ability to dimerize^36^. However, BR3 was designed on a structural basis, in which clusters of amino acids within the basic region exhibit a net positive charge. This differs from C/EBPα-K2Q mutations, which was selected based on the acetylation sites catalysed by GCN5. This is consistent with the idea that the C/EBPα-K2R, which retains a positive charge on these residues, is indistinguishable from WT C/EBPα in its behaviour in DNA binding and transactivation. These data show that the positive charge on K298 and K302 located within the basic region is required for DNA binding. In fact, our molecular simulation data predicted that acetylation disrupts the capacity to bind DNA by neutralizing the positive charge on these lysine residues. It is important to maintain a positively charged cluster of amino acids for the interaction with DNA. In agreement with these data and by using EMSA and ChIP assays, we demonstrated that the acetylation mimetic mutation (K2Q) is sufficient to decrease DNA binding. The C/EBPα-K2Q mutant (or acetylated C/EBPα) lacks DNA binding; however, it does not display dominant negative function over C/EBPα WT.

In comparison with C/EBPα WT, various mutants differ in the ability to drive granulocytic differentiation. We focused on the complete lack of transactivation and/or granulocytic differentiation consistently observed in K2Q and K3Q mutants of C/EBPα. K326 lies in the dimerization domain of C/EBPα, and substitution with glutamine (an acetylation mimetic) also prevented differentiation compared with C/EBPα WT and K326R. Such observations could also be due to the differences in protein–protein interactions, other than the homodimerization, which is required for efficient granulocytic differentiation. Transcription factor Foxo1 acetylation has been linked to decreased ability to bind DNA, and to become phosphorylated^37^. It will be intriguing to examine whether there is crosstalk between different PTMs on C/EBPα. Furthermore, substitution of lysine with arginine creates potential new methylation sites resulting in altered differentiation potential of mutant C/EBPα. At the very least, substitution of lysine with glutamine (acetylation mimetic) eliminates the potential for such self-modification sites.

To investigate the role of GCN5 in hematopoiesis, we crossed *Gcn5*^fl/fl^ mice with hematopoietic-specific *Vav-iCre*. We did not observe any substantial alteration in myeloid lineage commitment and differentiation in *Gcn5* excised mice (*Gcn5*^Δ/Δ^*Vav-iCre*^+^). However, by testing *in vitro* granulocytic terminal maturation in a more refined experiment aimed at analysing the effect of G-CSF on LSK cells from *Gcn5*^Δ/Δ^*Vav-iCre*^+^, we observed a 1.7-fold increase in Gr-1^+^ Mac-1^+^ cells. The possibility exists that this model, in which GCN5 was deleted at the earliest stages of embryonic hematopoietic development, does not represent an ideal model to study C/EBPα-dependent myeloid commitment in adult hematopoiesis. We cannot rule out the possibility that C/EBPα acetylation may be compensated by other HATs. PCAF shares high homology (88%) with GCN5 in the acetyltransferase domain, and is reported to be redundant with GCN5 (refs 38, 39, 40). While we did not observe PCAF-mediated repression of C/EBPα transactivation potential in transient transfection assays, given these previous reports of redundancy of GCN5 and PCAF *in vivo*, future studies involving GCN5 and PCAF double knockout mice could highlight the role of C/EBPα acetylation in hematopoietic cells.

GCN5 has been reported to be highly expressed in non-small cell lung cancer, which correlates with tumour size^41^. GCN5 also represses PGC-1α and β transcriptional activity via acetylation in cultured hepatocytes in mouse liver tissues and in primary skeletal muscle cells. This acetylation has been linked to changes in glucose concentration and controls expression of metabolic genes involved in fatty acid oxidation^16^,^42^. C/EBPβ is also reported as a target of GCN5-mediated acetylation during glucocorticoid stimulated preadipocyte differentiation of 3T3L1 and NIH3T3 cells^43^. Given the role of C/EBPα in lung development, glucose metabolism and adipogenesis, it would be of interest to investigate whether C/EBPα acetylation participates in these cellular processes in addition to its critical role in granulocytic differentiation. C/EBPα is inactivated in leukaemia by diverse molecular mechanisms. In this study, we demonstrated C/EBPα PTM (acetylation) in primary leukaemia cells, which may affect its activity. It will be of great interest to investigate whether the administration of a GCN5 inhibitor represents a novel therapeutic approach for the treatment of leukaemias.

## Methods

### Cell Culture

HEK293T (ATCC, CCL11268) cells were maintained in DMEM (Biowest) supplemented with 10% heat-inactivated FBS (Biowest). BOSC23 (ATTC, CRL11270), HL-60 (ATCC, CCL240), K562 (ATCC, CCL246) and Molm-14 (kindly provided by Chng Wee Joo, Cancer Science Institute, Singapore) were maintained in RPMI 1640 supplemented with 10% heat-inactivated FBS (Biowest). For inhibition of lysine deacetylases, K562 C/EBPα-ER stable cell lines were maintained in phenol red-free RPMI 1640 supplemented with 10% charcoal-stripped FBS (Invitrogen). K562, HL-60 and Molm-14 identities were confirmed by STR profiling (Genetica DNA Laboratories, NC, USA). All cell lines are tested negative for mycoplasma contamination by MycoAlert PLUS mycoplasma detection kit (Lonza).

32Dcl3 cells (kindly provided by Motomi Osato, Cancer Science Institute, Singapore) were maintained in RPMI 1640 supplemented with 10% FBS and IL-3 (5 ng ml^−1^; PeproTech). Induction of granulocytic differentiation was performed with removal of IL-3 by three washes with PBS and addition of recombinant human G-CSF (100 ng ml^−1^; PeproTech). Cells were harvested for western blot analysis 4 days post induction.

Bone marrow-derived CD34^+^ cells (1 × 10^6^ cells) were obtained from Stem Cell Technologies and cultured in IMDM (ATCC) supplemented with 10% FBS, FLT3 ligand (50 ng ml^−1^), SCF (50 ng ml^−1^), IL-6 (50 ng ml^−1^) and G-CSF (50 ng ml^−1^; PeproTech) to induce granulocytic differentiation. IL-6 was replaced with IL-3 (10 ng ml^−1^) and G-CSF was reduced to 20 ng ml^−1^ in the culture media from day 4 onwards. Fresh media was replenished every 3 days and cells were harvested on day 7 for western blot analysis.

### Patient AML samples

We obtained frozen bone marrow blasts of AML from patients at the National University Hospital, Singapore. The institutional review board of the National University of Singapore has approved the study. All patients provided written informed consent in accordance with the Declaration of Helsinki Principles. A summary of leukaemia status of the patient samples is described in Supplementary Table 2.

### Plasmid construction

WT 42-kDa human C/EBPα was cloned into the pcDNA6 V5-His-tagged plasmid. All mutations from lysine to either glutamine or arginine were created by site-directed mutagenesis (Agilent). All mutant constructs created by PCR were sequence verified. The following C/EBPα mutant expression constructs were created in the pcDNA6 V5-His backbone: C/EBPα K298Q, C/EBPα K302Q, C/EBPα K326Q, C/EBPα K298, 302Q (K2Q), C/EBPα K298, 302, 326Q (K3Q), C/EBPα K275, 277, 278Q (KIIIQ), C/EBPα K326R, C/EBPα K298, 302R (K2R) and C/EBPα K298, 302, 326R (K3R). For C/EBPα-ER receptor fusion protein, the 42-kDa (‘wild-type') human C/EBPα construct and the respective C/EBPα mutants were subcloned from pcDNA6 plasmids into the pBabe-ER plasmid (provided by Alan Friedman, Johns Hopkins University School of Medicine, Baltimore, USA) digested with *Bam*HI and *Xho*I, such that they are in frame with the C-terminal ER ligand-binding domain. Lentiviral shRNAs targeting GCN5 were purchased from MISSION shRNA at Sigma-Aldrich. For generation of pMIG-C/EBPα WT and mutant expression constructs, the respective C/EBPα inserts were subcloned from pcDNA6 plasmids into pMIG plasmid digested with *Eco*RI and *Xho*I. C/EBPα deletion expression constructs were PCR amplified using pcDNA6 C/EBPα WT V5-His as template and cloned into the pcDNA6 V5-His plasmid using *Eco*RI and *Xho*I. Insert sequences were confirmed by the Sanger sequencing method.

### Luciferase reporter assays

The firefly luciferase reporter gene in the pXP2 vector is driven by four C/EBP-binding sites derived from G-CSF receptor promoter, starting from −57 to −37 bp upstream of the major transcription start site of the G-CSF receptor gene^28^. As an internal control plasmid for co-transfections, the pRL-null construct encoding a Renilla luciferase gene (Promega) was used^44^. Firefly and Renilla luciferase activities were determined 24 h post transfection with the dual-luciferase reporter assay system (Promega). Firefly luciferase readings were normalized against internal control Renilla luciferase and calculated as fold differences against the activity obtained for the reporter plasmid without C/EBPα transfection. Luciferase assays were done with p(CEBP)_4_TK (200 ng), pRL-null (10 ng), pcDNA6 expression plasmids for C/EBPα (5, 10, 20 or 30 ng), GCN5 or GCN5 (-HAT) (20, 50, 100, 200 ng) and GCN5 or p300 (1, 5, 20 or 50 ng), respectively. For GCN5 knockdown effect on C/EBPα transactivation, 100, 500 pg and 2.5 ng of pcDNA6 C/EBPα plasmid concentrations were used. C/EBPα acetylation mimetic and non-mimetic mutants luciferase assays were performed using 500 pg, 1 and 5 ng concentrations of respective pcDNA6 expression plasmids. Luciferase assays where pcDNA6 C/EBPα K2Q was compared with pcDNA6 C/EBPα K298Q or pcDNA6 C/EBPα K302Q 1, 5 and 50 ng plasmid concentrations were used (Supplementary Fig. 4a).

### *In vitro* acetylation assay

A GST-tagged GCN5 HAT domain expression construct was provided by Axel Imhof (LMU, Munich, Germany). GST-tagged GCN5 protein was produced and purified from *Escherichia coli* BL21. GST-tagged recombinant GCN5 (Catalogue #K311-381G) was also purchased from SignalChem. *In vitro*-translated C/EBPα protein was produced in a coupled transcription and translation rabbit reticulocyte lysate (Promega, Catalogue #L1170). Peptides were synthesized by first Base (Singapore; Supplementary Table 1). All peptides were purified by high-performance liquid chromatography to obtain >95% purity. Substrate peptides were incubated with 0.25 μ Ci [^3^H] acetyl coenzyme A (Amersham) and 0.1 μg of purified GCN5-GST acetyltransferase (HAT) domain in a 30 μl acetylation buffer containing 50 mM Tris-HCl (pH 8.0), 50 mM NaCl, 10% glycerol, 0.5 mM EDTA, 1 mM dithiothreitol, 10 mM sodium buytrate and 1 mM phenylmethylsulfonyl fluoride. Reaction mixtures were incubated at 30 °C for 45 min. Subsequently, the peptides were spotted onto P81 filter paper (Whatman). The filter paper was air-dried and measured for acetylation activity using liquid scintillation counting.

Mass spectrometric analysis performed on the *in vitro* acetylated C/EBPα peptides that cover K298, K302 and K326 lysine residues confirmed acetylation of these three residues and uncovered an additional acetylation site at K304 (Supplementary Fig. 1e–g). But as acetylation at K304 was not seen in the *in vitro* acetyltransferase assay with K304 in overlapping peptides (Supplementary Table 1 for P-7 and P-8; Fig. 1f, Supplementary Fig. 1e–g), no further studies on this lysine residue were pursued.

### Liquid chromatography mass spectrometry analysis

Peptide samples were analysed using nano-HPLC coupled to an LTQ Velos (Thermo Fisher Scientific). Peptides were trapped onto a C18 pre-column and separated on an analytical column using a 1 or 2-h gradient ranging from 2% to 8% acetonitrile/0.1% formic acid, followed by a 5 min gradient ranging from 8% to 80% acetonitrile/0.1% formic acid and stayed at 80% acetonitrile/0.1% formic acid for 20 min. MS scans ranged from 310 to 1,400 m/z, AGC target 3E4, and maximum injection time of 50 ms. The 10 most intense ions with an ion intensity above 1,000 and a charge state excluding one were sequentially isolated to a maximum AGC target value of 4E4 for a maximal 100 ms and fragmented by Electron Transfer Dissociation (ETD). The following parameters were employed for ETD: the reagent ion source temperature of 160°C, reagent ion source emission current of 50 μA, reagent ion source electron energy of −70.00 V, reagent ion source chemical ionisation of 20 p.s.i., reagent vial 1 ion time of 50, reagent vial 1 AGC target of 1E5, reagent vial 2 ion time of 50, reagent vial 2 AGC target of 1E5, supplemental activation energy of 15. A dynamic exclusion list was applied using an exclusion list size of 500, one repeat count, repeat duration of 45 s, exclusion duration of 30 s as well as a mass width of 1.0 low and 1.5 high. Expiration count was disabled.

### Data processing and database search

Raw file processing for the peptide samples was carried out using Mascot Daemon (version 2.3.2, Matrix Science). Data import filter for precursor masses was from 700 to 4,000 Da, with a minimum scan per group of 1 and a minimum peak count of 10. Mascot search was performed using the custom made database consisting of three peptide sequences only: RLRKRVEQLSRC, IAVRKSRDKAKQRC and RKSRDKAKQRNVEC. No enzyme cleavage was specified. Acetyl (C), Acetyl (K), Acetyl (N-term), Acetyl (S), Oxidation (M) were set as variable modifications. Tolerance for the precursor masses was 2 Da and for fragments 0.8 Da. The highest score of MS/MS spectrum for each intact unique acetylated peptide (with a minimum mascot score of 25) was manually validated.

### Generation of site-specific acetylation antibodies

Acetylation-specific C/EBPα antisera was raised in rabbits against keyhole limpet haemocyanin (KLH)-conjugated peptides, which comprised of the following residues of human C/EBPα: IAVRK^Ac^SRDKAKQR (Ac-K298 antiserum), IAVRKSRDK^Ac^AKQR(Ac-K302 antiserum) and RLRK^Ac^RVEQLSR (Ac-K326 antiserum), with a C-terminal cysteine residue added to the peptide sequence. Addition of cysteine was for the conjugation of the synthetic peptide to the carrier protein KLH. Antisera were purified by binding to acetylated peptide-conjugated Sulfolink gel (Pierce) and subsequently passed through an additional non-acetylated peptide-conjugated Sulfolink gel to eliminate non-acetylation-specific antibodies. Purified acetylation-specific antibodies were used at a dilution of 1:1,000 for western blotting.

### Immunoblotting and Immunoprecipitation

For whole-cell lysis, 1 × 10^7^ cells were lysed with radioimmunoprecipitation assay buffer. For subcellular fractionation, nuclear and cytosolic fractions were prepared using Nuclear Extract Kit from Active Motif. Protein concentrations were quantitated with Biorad Protein Assay. Proteins were separated on 10% SDS-PAGE gels. Immunoblots were incubated with primary antibody (Supplementary Table 5) overnight at 4 °C, followed by a secondary horseradish peroxidase-conjugated antibody at room temperature for 1 h.

For immunoprecipitation of C/EBPα, 800 μg of whole cell lysate was used. radioimmunoprecipitation assay buffer was diluted four times and used as immunoprecipitation buffer with antibody. Lysate was pre-cleared with rabbit IgG antibody (Cell Signaling Technology) and Dynabeads Protein A (Invitrogen), while antibody for immunoprecipitation was bound to Dynabeads Protein A for 1 h at 4 °C. Subsequently, the pre-cleared lysate was incubated with the beads-bound antibody for 3 h with constant rotation at 4 °C. Beads-bound immunoprecipitates were washed thrice with immunoprecipitation buffer. Co-immunoprecipiation of FLAG-tagged proteins with M2 agarose beads (Sigma) was performed according to manufacturer's instructions. Briefly, 1 mg of whole cell lysate was lysed in lysis buffer (50 mM Tris-HCl, 150 mM NaCl, 1 mM EDTA, 1% Triton X-100) and incubated with anti-FLAG M2 agarose beads overnight at 4 °C. Beads-bound immunoprecipitates were washed thrice with TBS.

Proteins were eluted from the beads under acidic conditions with 0.1 M glycine, pH 2.3 at room temperature for 5 min with constant shaking. Elutant was neutralized with 1/10 volume of 0.5 M Tris, pH 7.4 with 1.5 M NaCl and analysed with immunoblotting.

### Computational methods

The crystal structure of the C/EBPα-DNA complex (known as C/EBPα WT-DNA) was downloaded from the Protein Data Bank (PDB code: 1NWQ)^18^. To generate the coordinates for the acetylation mimetic C/EBPα-K2Q complexed with DNA (K2Q-DNA), K298 and K302 residues were replaced with glutamine using the UCSF Chimera package^45^. The GLN rotamers from the Dunbrack library were used^46^. The acetylated lysine residues (K2Ac) were built manually with UCSF Chimera. The H–N–C=O dihedral angle of all four acetylated lysine residues were set to 0° in the first acetylated model (hereafter termed as K2Ac_a-DNA) based on the most populated structures derived from computations^47^. However, the acetylated lysine residues in recently published crystal structures were found to have the H–N–C=O dihedral angles with both 0° and 180° (Fig. 3b)^48^,^49^,^50^. Thus, the H–N–C=O dihedral angle of all four acetylated lysine residues were set to 180° in the second acetylated model (K2Ac_b-DNA). The backbone atoms of the four models showed similar fluctuations during the 3.2 ns long MD simulations (Supplementary Fig. 3). However, longer MD trajectories are needed to meaningfully discuss the stability of the four models. The scope of the current computational study is to gain insight into how different mutations affect protein–DNA-binding energy and protein-DNA hydrogen bonding. For this purpose, the relatively short MD trajectories presented here should be sufficient^51^. The ff03 molecular mechanics (MM) force field was assigned for all residues^52^. The acetylation of partial charges derived for use with the ff03 force field were taken from literature with corresponding atom names and atom types^47^.The force field parameters were set up using the leap programme of the AmberTools13 package^53^. Na^+^ counterions were added to neutralize the protein–DNA model systems. The crystallographic water molecules were retained in all four model systems. Each protein–DNA model complex was placed into a pre-equilibrated TIP3P truncated octahedral water box^54^. The crystallographic water molecules were included in the TIP3P solvent boxes which extended at least 12 Å from the protein-DNA complex.

MM minimizations and MD simulations were carried out with the sander programme of the AMBER12 package^53^.The MD simulations were preceded by three minimization steps: (1) solute atoms were held fixed while solvent molecules were relaxed (1000 steps of steepest descent and 1,000 steps of conjugate gradient minimization); (2) solvent atoms were held fixed while solute molecules were relaxed (1,000 steps of steepest descent and 1,000 steps of conjugate gradient minimization); (3) both solvent and solute were optimized without any restraints (1,000 steps of steepest descent and 4000 steps of conjugate gradient minimization). After minimization, a two-step MD simulation was carried out on all systems with a 2 kcal mol^−1^Å^2^ restraint on the solute atoms: (1) each system was heated from 0 to 300 K over a period of 50 ps in the canonical (NVT) ensemble; (2) each system was relaxed for 50 ps in the isothermic-isobaric (NPT) ensemble (*T*=300 K and *P*=1 atm). Finally, production MD simulations were run for 3.2 ns in the NPT ensemble (*T*=300 K and *P*=1 atm). Force calculations were performed with periodic boundary conditions and a 9 Å cutoff was used for non-bonded interactions. Covalent bonds connecting hydrogen atoms were constrained with SHAKE^55^. A collision frequency of 3 ps^−1^ was used in the Langevin thermostat to maintain the system temperature^56^. A timestep of 2 fs was used in all MD simulations. Atom coordinates were saved every 10 ps during the MD simulations (100 frames ns^−1^). The random number generator seed was altered at every restart of the MD simulations.

The polar desolvation free energy was calculated with the *MMPBSA.py* program^57^. The last 3 ns of the MD trajectories were used for Molecular Mechanics/Poisson−Boltzmann Surface Area (MM/PBSA) calculations and hydrogen bond analysis. The radii optimized by Tan *et al*.^58^ were used with a 0.5 Å grid spacing, a 1.4 Å solvent probe radius, a protein dielectric constant of 1.0, and a solvent dielectric constant of 80. Due to the expensive computational demand, entropies were not considered. MM/PBSA calculations can yield satisfactory results without the inclusion of entropy^59^.

### Generation of stable K562 cell lines

To generate WT or mutant C/EBPα lines, K562 cells were stably transfected by electroporation with the respective pBabe-C/EBPα-ER fusion constructs. Briefly, 2 × 10^6^ cells were electroporated in an Amaxa apparatus with 5 μg of *Sca-1* linearized plasmid. At 48 h post-transfection, cells were subjected to selection with 0.5 μg ml^−1^ puromycin and 50 cells were seeded per 96-well plate. C/EBPα-ER nuclear translocation was induced by addition of 5 μM of β-estradiol (Sigma, E2758). Cells were assessed 4 days post induction for differentiation by CD11b surface marker expression, morphology and neutrophilic enzymatic activity^4^. Multiple individual clones for each type of mutant were picked and analysed by western blot to confirm expression of the fusion protein. The results from two representative clones are presented in this study.

### Assessment of granulocytic differentiation

Nitroblue tetrazolium analysis with 2.5 × 10^5^ cells was done as previously described^4^. The percentage of nitroblue tetrazolium-positive cells was quantified by counting at least 100 cells for each mutant under brightfield microscopy (Nikon). Cell morphology was assessed by Wright-Giemsa staining. Cytospin preparation of 2.5 × 10^5^ cells were done as previously described^60^. For staining of CD11b surface marker, 1 × 10^6^ cells were stained with CD11b antibody (Supplementary Table 6) and analysed using LSRII flow cytometry (BD Biosciences). The resulting data were processed with FlowJo Version 7.6.4 software (TreeStar).

### shRNA lentivirus packaging and transduction

Lentiviral plasmids (pLKO.1-puro) encoding GCN5-specific shRNA (TRCN0000038879 (shGCN5 #1), TRCN0000294386 (shGCN5 #2), TRCN0000307319 (shGCN5 #3)) and non-targeting control shRNA (SHC0002 (NTC #1), SHC007 (NTC #2)) were purchased from Sigma MISSION. Lentivirus packaging was performed in 293T cells by co-transfecting shRNA lentiviral plasmids with pCMV-dR8.91and pCMV-VSVG using Lipofectamine 2000. For GCN5 knockdown, cells were exposed to viral particles with multiplicities of infection (MOI) ranging from 1 to 2, in the presence of 8 μg ml^−1^ polybrene (Santa Cruz) for 24 h. Cells were selected in media containing 1 μg ml^−1^ puromycin (Sigma) at 48 h post transduction, and checked for knockdown efficiency by immunoblotting at 2 weeks post transduction.

### Retroviral transduction of *Cebpa* ^Δ/Δ^ LSK cells

Animal work was done at NUS with approval from IACUC. C/EBPα conditional knockout (*Cebpa*^fl/fl^) and *Mx1-Cre* mice have been described previously^2^.

### Liquid culture of GCN5 LSK cells

Animal work was done at NUS with approval from IACUC. *Gcn5*^Δ/Δ^*Vav-iCre* knockout mice were generated by crossing *Gcn5*^fl/fl^ and *Vav-iCre* mice^25^,^26^. Cells (5,000–10,000) were added to grow in Stemline II hematopoietic stem cell expansion medium (Sigma-Aldrich) supplemented with 1 × Penicillin-Streptomycin, 1 × anti-mycotic cocktail, 6 ng ml^−1^ IL-3, 10 ng ml^−1^ IL-6, 20 ng ml^−1^ stem cell factor (SCF), 20 ng ml^−1^ G-CSF and 10% FBS. Cells were grown for 7 days before proceeding with FACS and morphological analysis.

### Electrophoretic mobility shift assay

EMSA was done as previously described^4^. Briefly, EMSA was performed with nuclear extracts from K562 C/EBPα lines. The oligonucleotides used are derived from the G-CSF receptor promoter (−57 to −38 bp), the sequences are as follows (C/EBP binding sites are underlined): upper strand, 5′-CTAGGGCTTGCGCAATCTATATTCG-3′, lower stand, 5′-CGAATATAGATTGCGCAAGCCCTA-3′. EMSAs were performed by incubating 10 μg of nuclear extracts with 50,000 counts per minute double-stranded oligonucleotides in a 25 μl reaction mixture containing 10 mM HEPES-KOH buffer (pH7.9), 50 mM KCl, 2.5 mM MgCl_2_, 1 mM DTT, 10% glycerol, 1 μg bovine serum albumin (BSA) and 0.5 μg poly(dI-dC) on ice for 20 min. For supershift assay, 2 μg of anti-C/EBPα antibody (14AA) or anti-ER (HC-20) was added to the binding reaction. Binding reactions were resolved on a 6% non-denaturing polyacrylamide gel with 0.5 × TBE (0.089 M Tris borate and 0.002 M EDTA) and electrophoresed at 140 V at 4 °C.

### Chromatin immunoprecipitation

ChIP was performed with 2 μg anti-ER antibodies on K562 C/EBPα-ER cells (2 × 10^7^ cells). Briefly, cells were induced with 5 μM β-estradiol for 45 min, fixed in 1% formaldehyde for 5 min at room temperature and subsequently quenched with addition of glycine to a final concentration of 200 mM. Cells were lysed with Buffer L1 (50 mM Tris pH 8.0, 2 mM EDTA, 0.1% NP-40, 10% glycerol) for 5 min on ice, centrifuged for 10 min at 850 × g. The nuclear pellet was resuspended in Buffer L2 (50 mM Tris pH 8.0, 5 mM EDTA, 1% SDS). Crosslinked chromatin was sonicated using Bioruptor (Diagenode) at ‘high' amplitude, 12 s on/30 s off for 16 cycles, and centrifuged for 10 min at 17,000*g*. Sonicated material was diluted with 9 volumes of Buffer DB (50 mM Tris, 200 mM NaCl, 5 mM EDTA, 0.5% NP-40) and pre-cleared with salmon sperm DNA (Sigma), rabbit IgG (Santa Cruz) and Dynabeads Protein A (Invitrogen). Pre-cleared chromatin was immunoprecipitated overnight with ERα (HC-20) antibody, followed by addition of Dynabeads Protein A for 30 min at 4 °C. Beads were washed thrice with washing buffer (WB) (20 mM Tris pH 8.0, 500 mM NaCl, 2 mM EDTA, 0.1% SDS and 1% NP-40), once with WB plus 500 mM LiCl, thrice with buffer TE. Bound chromatin was eluted from the beads with 100 μl of elution buffer (1x TE, 2% SDS). After reverse-crosslinking with RNase A and proteinase K digestion, DNA was purified by Qiaquick PCR purification kit and eluted with 50 μl of TE buffer. Primers used are indicated in Supplementary Table 7. qPCR was carried out with GoTaq qPCR master mix (Promega) and analysed with a Corbett Rotorgene system. Relative enrichment is calculated over the inactive involucrin locus.

### Dimerization assay

293T cells were co-transfected with 10 μg of V5-tagged WT or mutant versions of C/EBPα and 10 μg of FLAG-tagged WT C/EBPα, cell lysates were collected 24 h after transfection. Anti-FLAG M2 Affinity gel (Sigma) was used to co-immunoprecipitate FLAG-tagged proteins. Samples were kept under constant rotation for 2 h at 4 °C. Beads-bound immunoprecipitates were washed thrice with TBS buffer and eluted in SDS-PAGE sample buffer for immunoblotting analysis.

### Gene expression analysis

Raw microarray gene expression data (CEL files) for AML and normal BM CD34^+^ samples were taken from GSE13159 (ref. 61) and GSE19429 (ref. 62), respectively. We used the RMA model to extract the gene intensity from multiple probe intensities, while the cross-array data normalization was performed using the Cross-Correlation method^63^.

### Statistical analysis

The two-tailed, Student's t-test was used to compare between two groups. Asterisk (*) indicates *P* value<0.05, ** indicates *P* value<0.01, *** indicates *P* value<0.001 in the figures.

## Additional information

**How to cite this article:** Bararia, D. *et al*. Acetylation of C/EBPα inhibits its granulopoietic function. *Nat. Commun.* 7:10968 doi: 10.1038/ncomms10968 (2016).

## Supplementary Material

Supplementary Information Supplementary Figures 1-12 and Supplementary Tables 1-7

## Figures and Tables

**Table 1:** C/EBPα and DNA interaction energy.

	Average	s.d.	s.e.m.
WT-DNA	−84.5	15.7	0.9
K2Q-DNA	−43.5	15.1	0.9
K2Ac_a-DNA	−68.9	13.3	0.8
K2Ac_b-DNA	−56.6	10.9	0.6

s.d., standard deviation; s.e.m., standard error of the mean; WT, wild type.

Calculated MM/PBSA protein–DNA-binding free energies (kcal mol^**−**1^) based on 3 ns MD trajectories.
