# Supplementary material for: Acetylation of C/EBPα inhibits its granulopoietic function
Source: Nat Commun. 2016 Mar 23;7:10968. doi: 10.1038/ncomms10968 (PMC4814574; doi:10.1038/ncomms10968)
Supplement: Supplementary Information — Supplementary Figures 1-12 and Supplementary Tables 1-7 [file ncomms10968-s1.pdf]

Supplementary Information

Supplementary Figure 1

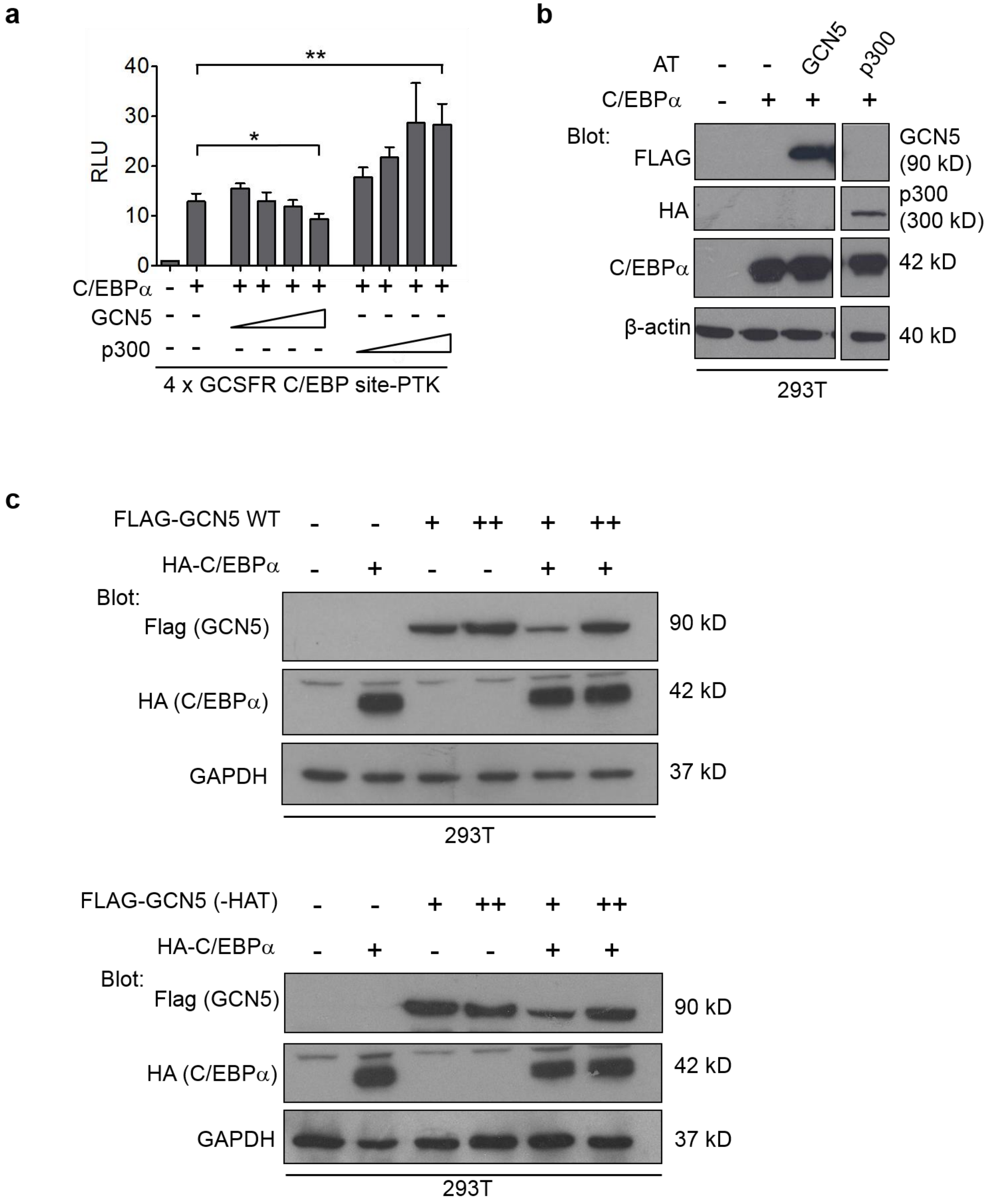

Supplementary Figure 1 (cont.)

d

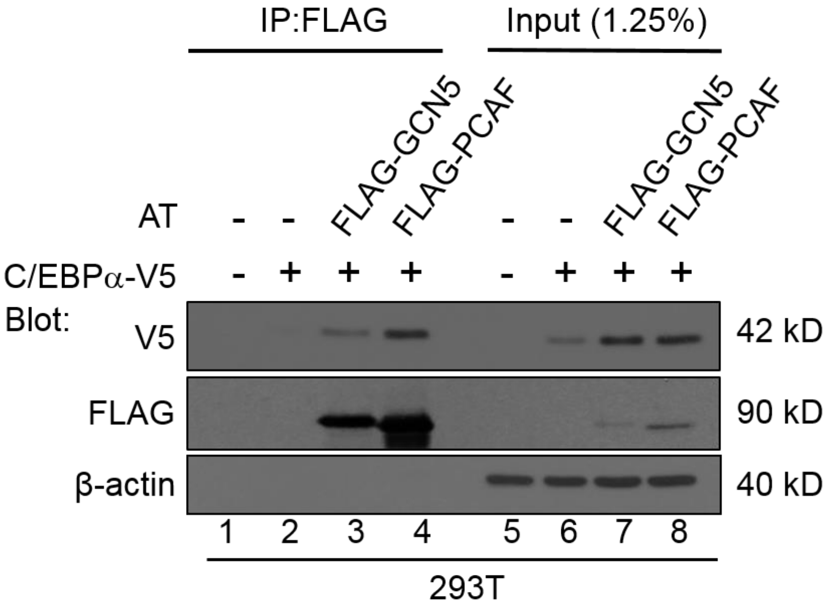

e

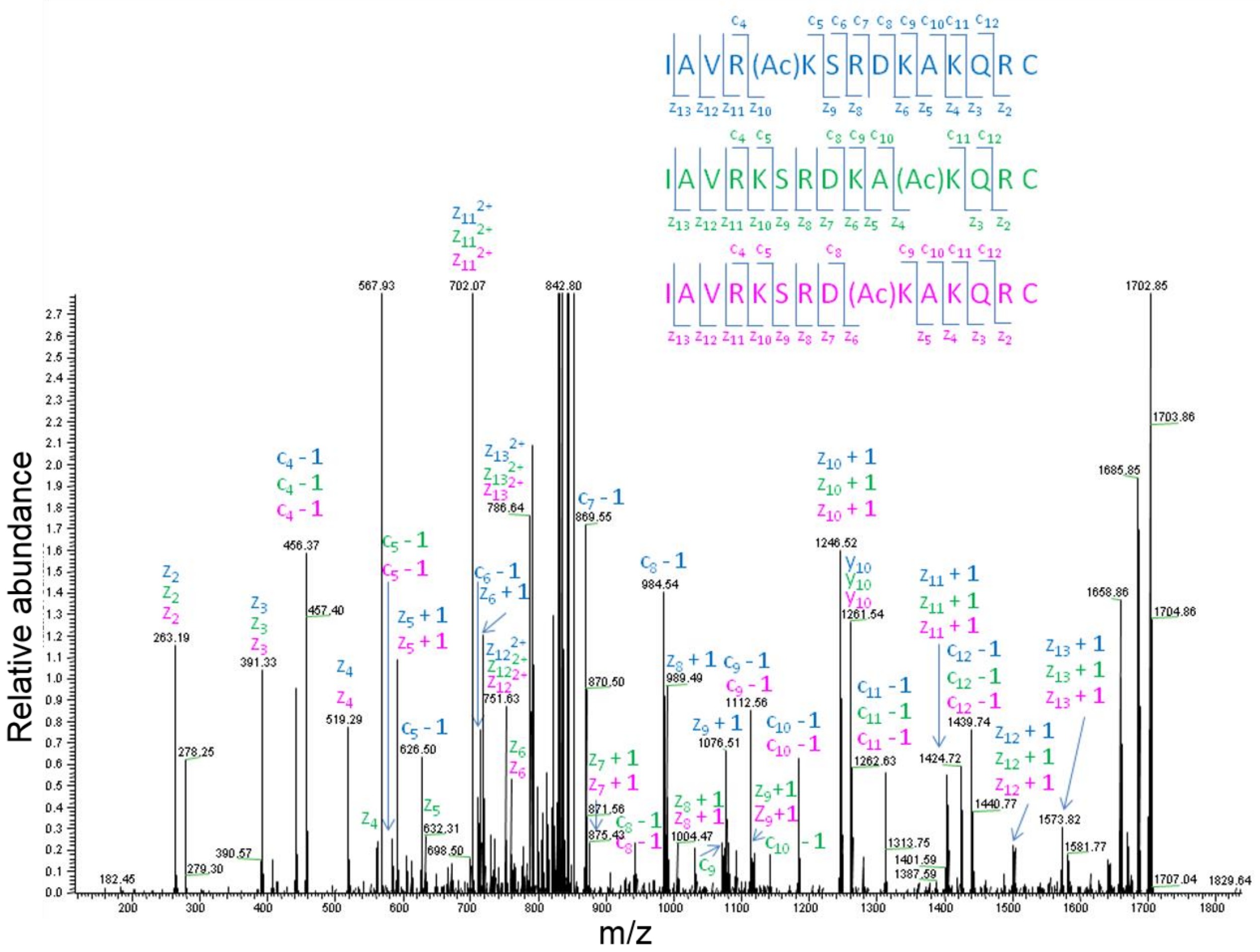

Peptide Sequence	Theoretical Mass	Observed Mass
IAVRKSRDKAKQRC	1657.9522	1657.4947

Supplementary Figure 1 (cont.)

f

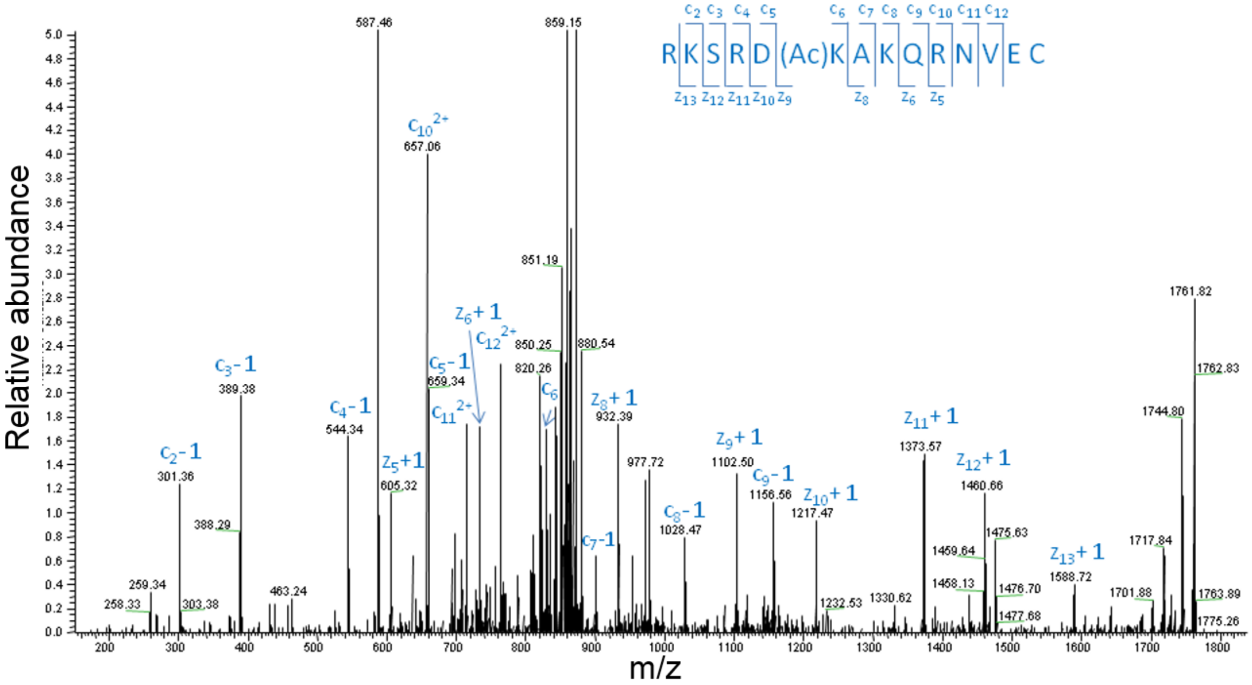

Peptide Sequence	Theoretical Mass	Observed Mass
RKSRDKAKQRNVEC	1716.9165	1717.7461

g

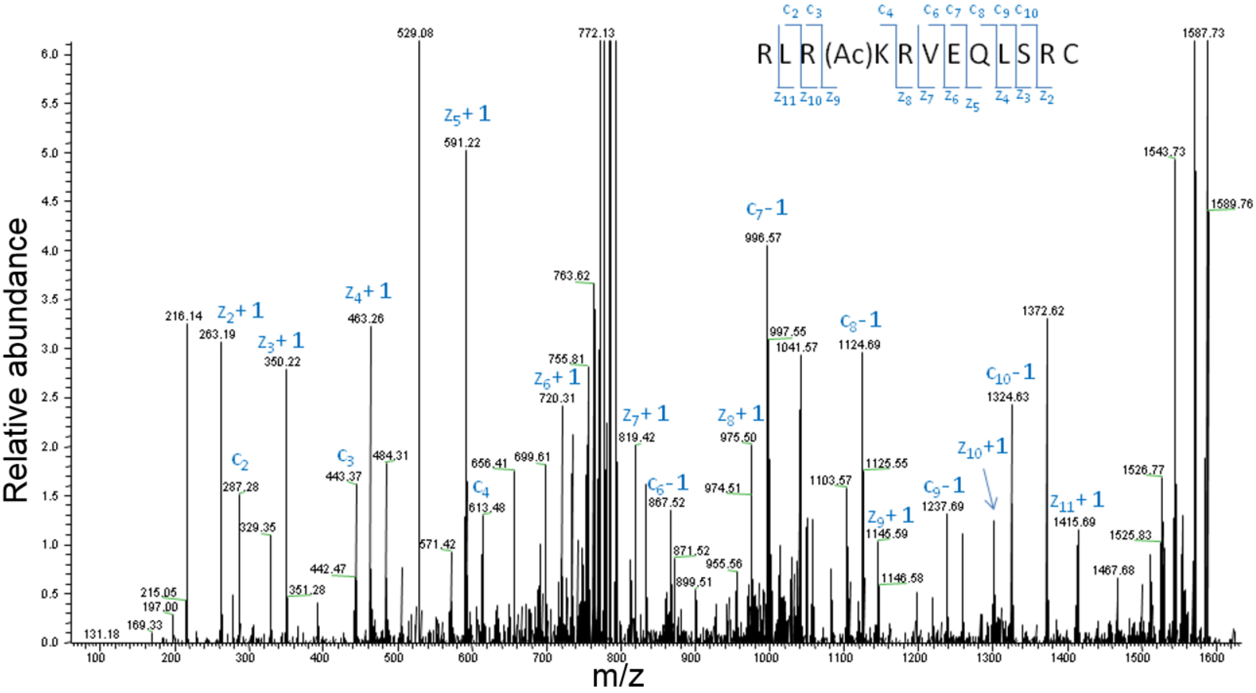

Peptide Sequence	Theoretical Mass	Observed Mass
RLRKRVEQLSRC	1542.8889	1542.7420

Supplementary Figure 1 (cont.)

h

```
NP_004355.2 (human)        293 IAVRKSRDKAKQRNVETQQKVLELTSDNDRLRKRVEQL 331
NP_031704.2 (mouse)        294 IAVRKSRDKAKQRNVETQQKVLELTSDNDRLRKRVEQL 332
NP_036656.1 (rat)          293 IAVRKSRDKAKQRNVETQQKVLELTSDNDRLRKRVEQL 331
NP_571960.1 (zebrafish)    223 IAVRKSRDKAKMRNVETQQKVIELSADNDRLRKRVEHL 261
NP_789741.2 (cow)          288 IAVRKSRDKAKQRNVETQLKVLELTSDNDRLRKRVEQL 326
NP_001186818.1 (pig)       283 IAVRKSRDKAKQRNVETQQKVLELTTDNERLRKRVEQL 321
NP_001080275.1 (frog)      240 IAVRKSRDKAKMRNVETQQKVFELSSDNDKLRKRVEQL 278
```

i

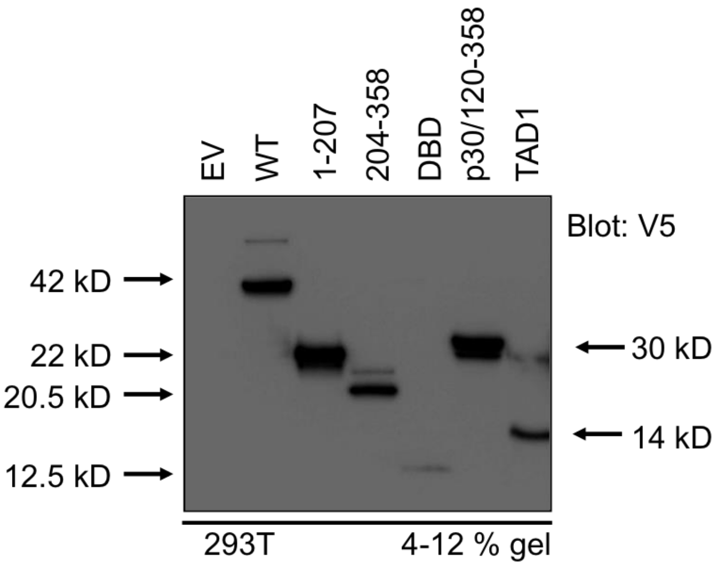

Supplementary Figure 1 (cont.)

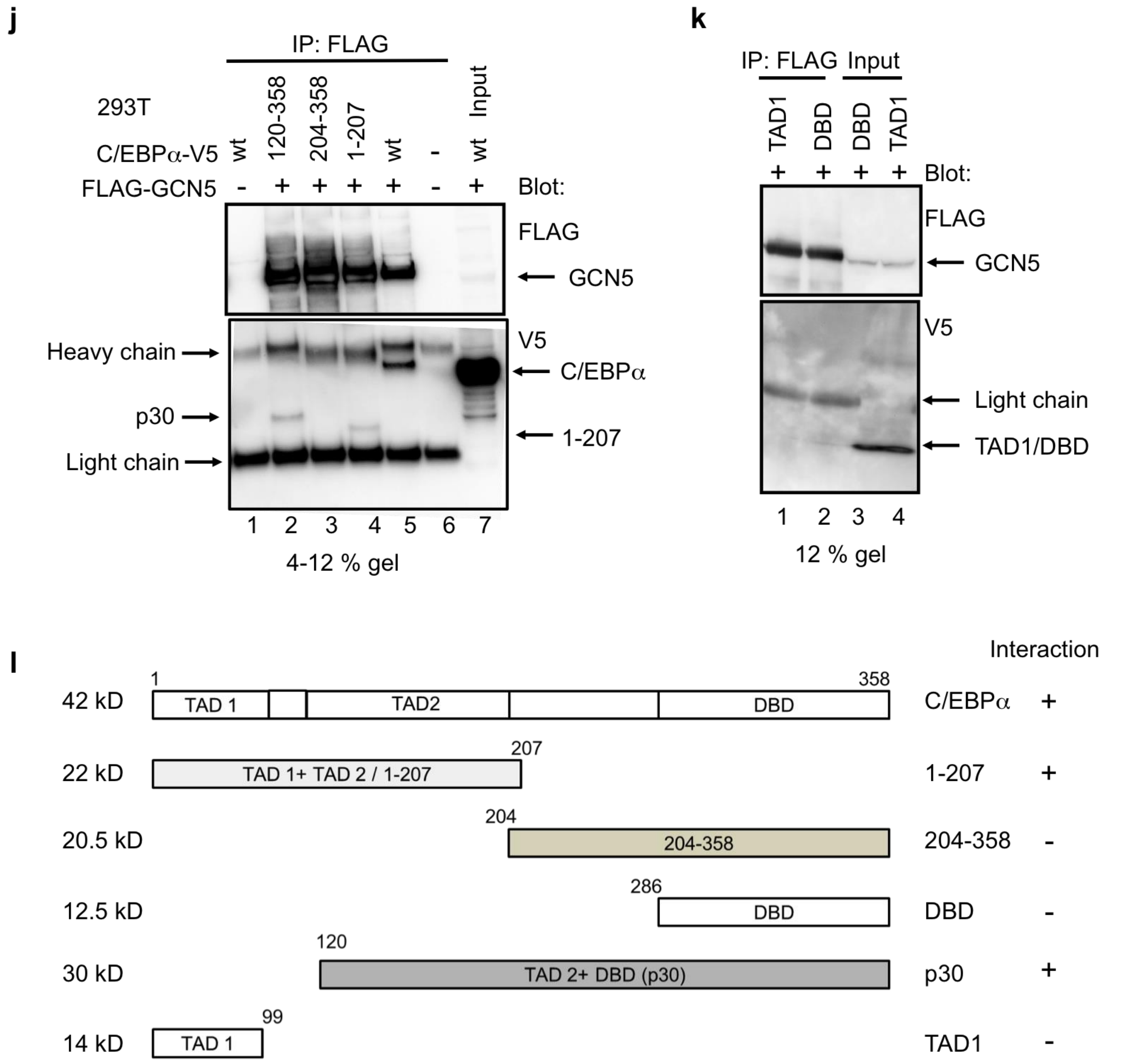

Supplementary Figure 1 (cont.)

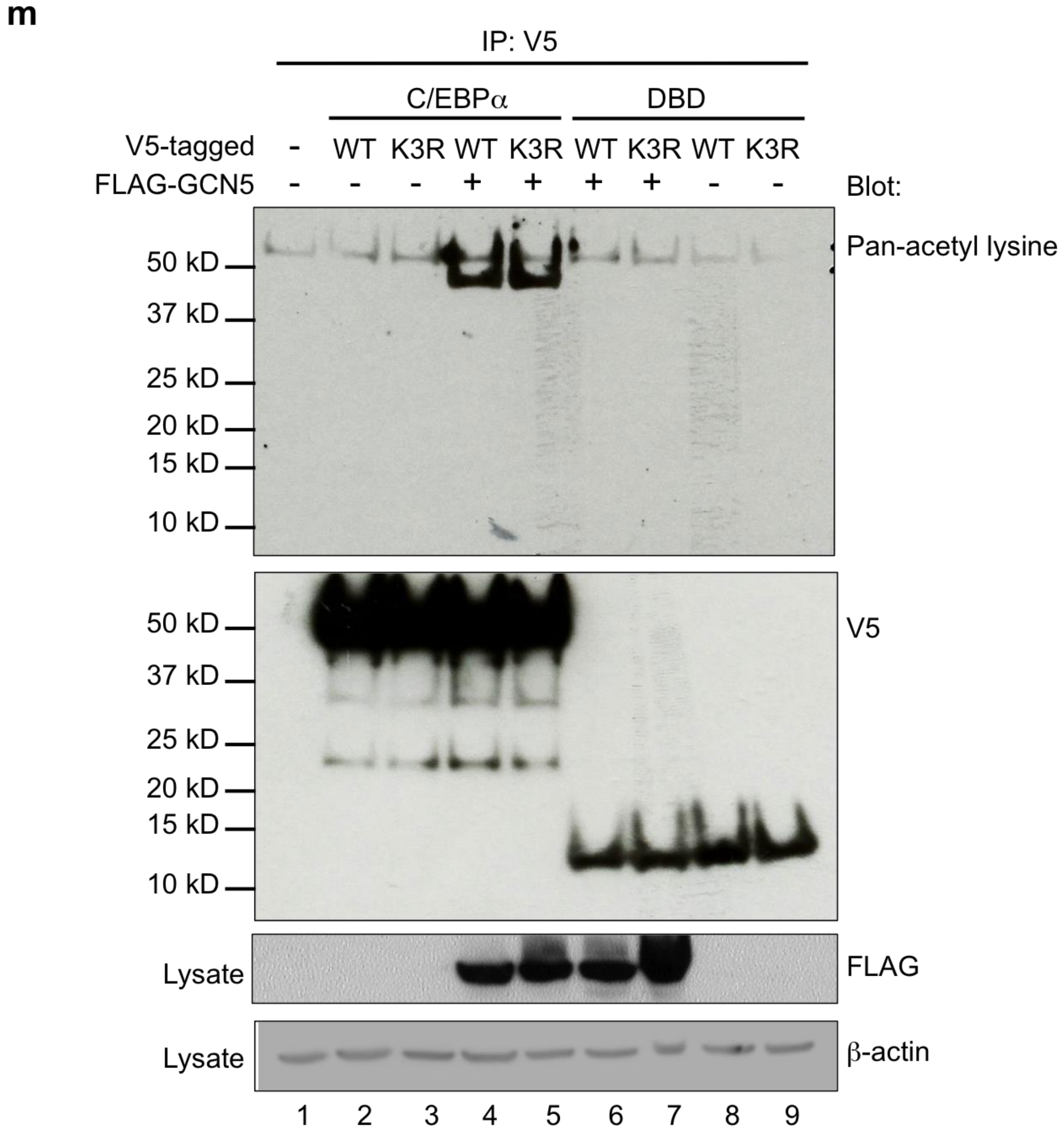

Supplementary Figure 1 (cont.)

n

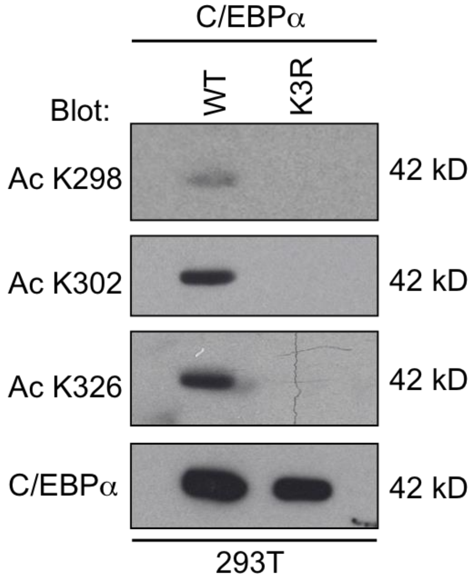

o

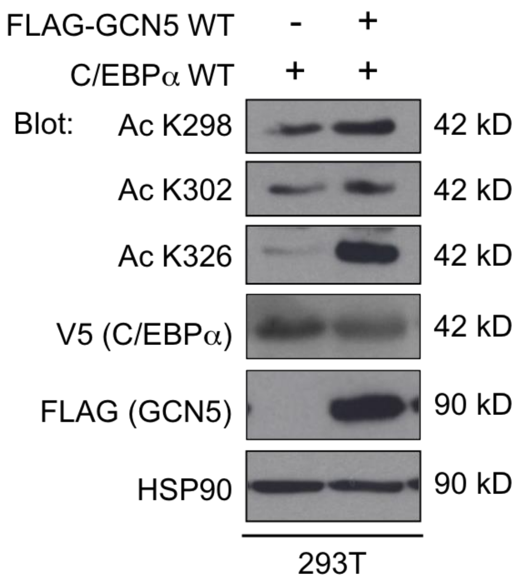

Supplementary Figure 1. GCN5 acetylates C/EBPα in C-terminus at K298, K302 and K326 while interaction domain maps in N-terminal region. All Western blots were performed by transient transfection in 293T cells.

(a) Effect of various acetyltransferases on C/EBPα transactivation. Luciferase activity was measured in duplicate and data are shown as mean±s.d. (N=3). *P < 0.05 and **P< 0.01; Student’s unpaired t-test.

(b, c) Western blot of protein expression of C/EBPα and various acetyltransferases used in Supplementary Figure 1a. AT denotes acetyltransferase. GCN5 or GCN5 (-HAT) mutant co-transfection does not alter C/EBPα protein levels as indicated by Western blot.

(d) C/EBPα interaction with GCN5 and PCAF. Co-immunoprecipitation (Co-IP) was performed using FLAG M2 beads.

(e, f, g) Mass spectra showing acetylation of C/EBPα at K298, K302, K304 (e) K302 (f), and K326 (g)

(h) C/EBPα K298, K302 and K326 are highly conserved. Human (NP_004355.2), mouse (NP_031704.2), rat (NP_036656.1), cow (NP_789741.2), zebrafish (NP_571960.1), pig (NP_001186818.1) and frog (NP_001080275.1).

(i) Expression of various C/EBPα deletion constructs. Proteins were resolved using 4-12% SDS-PAGE.

(j, k) C/EBPα interaction region is N-terminal domain with GCN5. V5-tagged C/EBPα WT, 1-207aa (TAD1+ TAD2), 204-358aa, p30 (120-358aa), were co-transfected with FLAG tagged GCN5. Whole cell lysate were incubated with FLAG M2 agarose beads. Positions of heavy and light chains were indicated. Similarly TAD1 and DBD regions of C/EBPα do not interact with GCN5. Positions of light chains were indicated.

(l) Diagram showed various C/EBPα interaction regions with GCN5. Molecular weight is shown on the left. Name and GCN5 interaction results were shown on the right. Interaction is indicated by + sign while loss of interaction by – sign.

(m) Pan-acetyl antibody signal for C/EBPα WT, K3R and DBD WT and K3R domain mutants upon co-transfection with GCN5.

(n) Test of site-specific acetyl antibodies specificity using whole cell lysates transfected either with C/EBPα WT or non-acetylated mimetic C/EBPα K3R.

(o) GCN5 acetylates C/EBPα at K298, K302 and K326. C/EBPα acetylation and protein levels were determined by immunoblotting.

(n, o) Transfected cells were treated with 400 nM TSA, 20 mM NA and 5 mM NB for 12-16 h prior to harvest.

Supplementary Figure 2

a

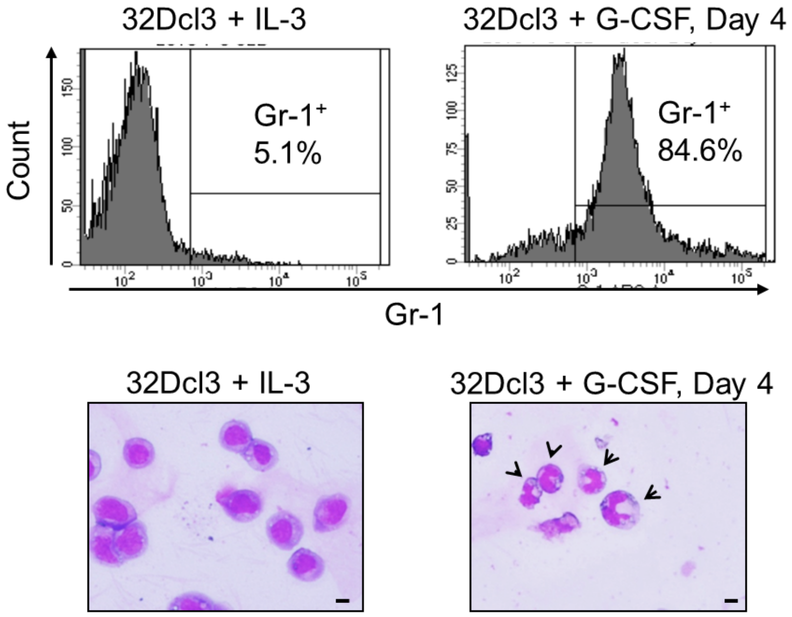

b

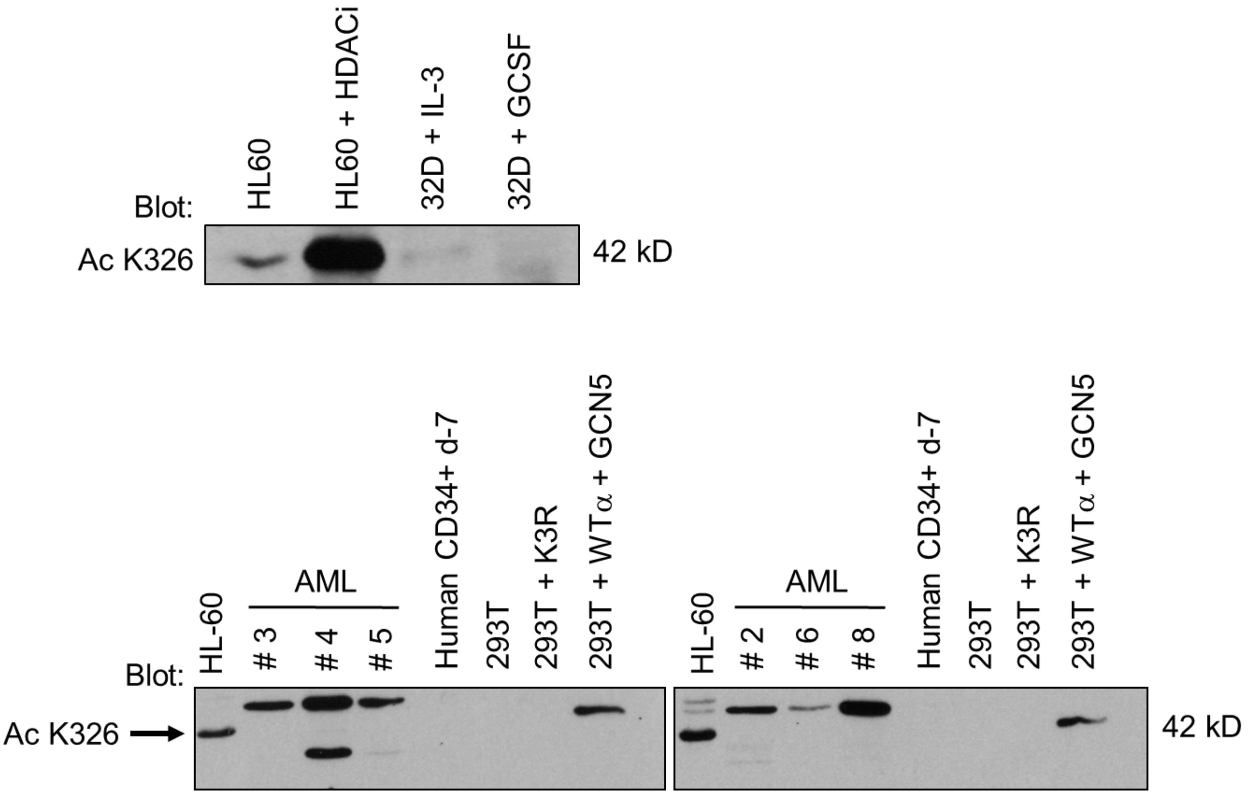

Supplementary Figure 2 (cont.)

c

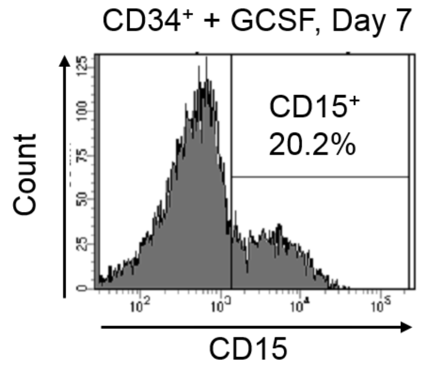

d

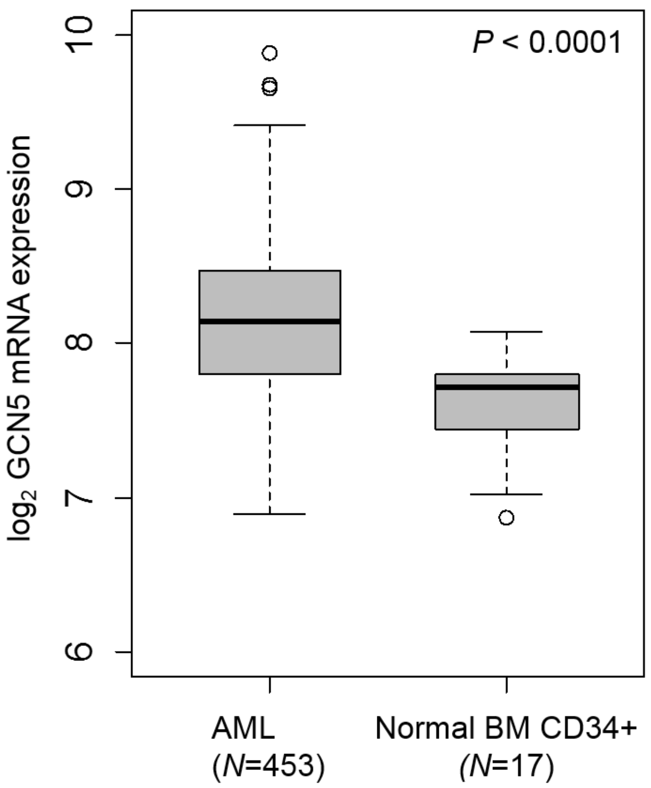

Supplementary Figure 2. G-CSF-induced granulocytic differentiation in 32Dcl3 murine myeloid cells and $CD34^{+}$ human hematopoietic progenitor cells.

(a) Gr-1 expression in 32Dcl3 murine cells upon G-CSF induction. Cells were cultured in the presence of G-CSF for the indicated time, stained with Gr-1, and analyzed by flow cytometry. FACS histograms plots of 32Dcl3 with IL-3 as a control and G-CSF induction were shown. 32Dcl3 cytospins in the presence of IL-3 and on day 4 of stimulation with G-CSF were shown by Giemsa staining. Original

magnification x 100, scale bars indicate 10 μm. Arrows indicate granulocytes with their polymorphonuclear morphology.

(b) C/EBPα acetylation at K326 is not detected in 32Dcl3 cells grown in IL-3 and after G-CSF induction. C/EBPα acetylation at K326 is not detected in human AML and partially differentiated human $CD34^+$ cells. Whole cell lysates were prepared and blotted with anti-acetyl K326. Non-acetylated mimetic C/EBPα K3R was used as a negative control for acetylation; HL-60 along with 293T co-transfected with C/EBPα WT and GCN5 cells were used as a positive control for acetylation.

(c) $CD34^+$ cells were partially differentiated with addition of G-CSF for 7 days. Granulocytic differentiation is confirmed by surface marker, CD15, by flow cytometry.

(d) Higher *GCN5 (KAT2A)* gene expression profile in AMLs (N=453) compared to normal human bone marrow $CD34^+$ cells (N=17). Box plots of mRNA expression levels are shown. The boxes indicate the upper and lower quartiles. The band within the boxes represents the median and small circles represent the outlier. $P < 0.001$; Student's unpaired t-test.

Supplementary Figure 3

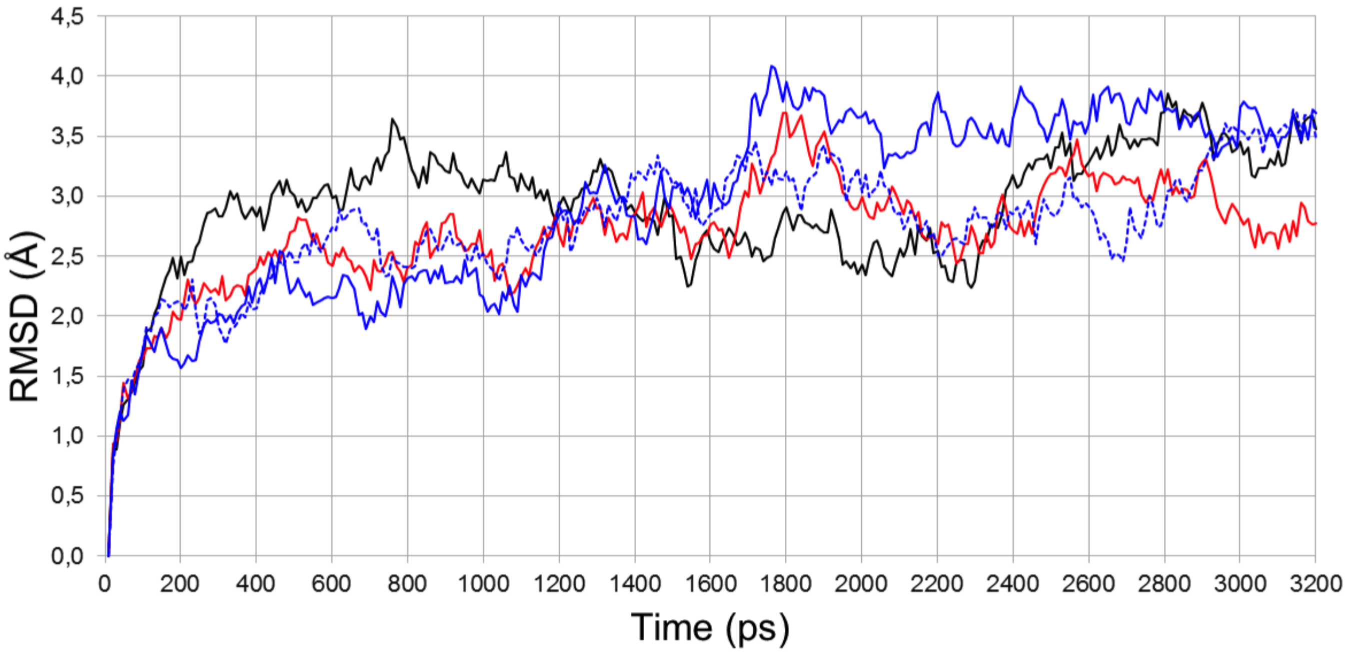

Supplementary Figure 3. Comparison of root mean square deviation of acetylation mimetic model and acetylated lysine model of C/EBPα

Protein and DNA backbone root mean square deviations (RMSD) for configurations taken at 10 ps intervals from the WT‑DNA (black) K2Q‑DNA (red), K2Ac_a‑DNA (blue) and K2Ac_b‑DNA (dashed blue) MD simulations relative to the first frame.

Supplementary Figure 4

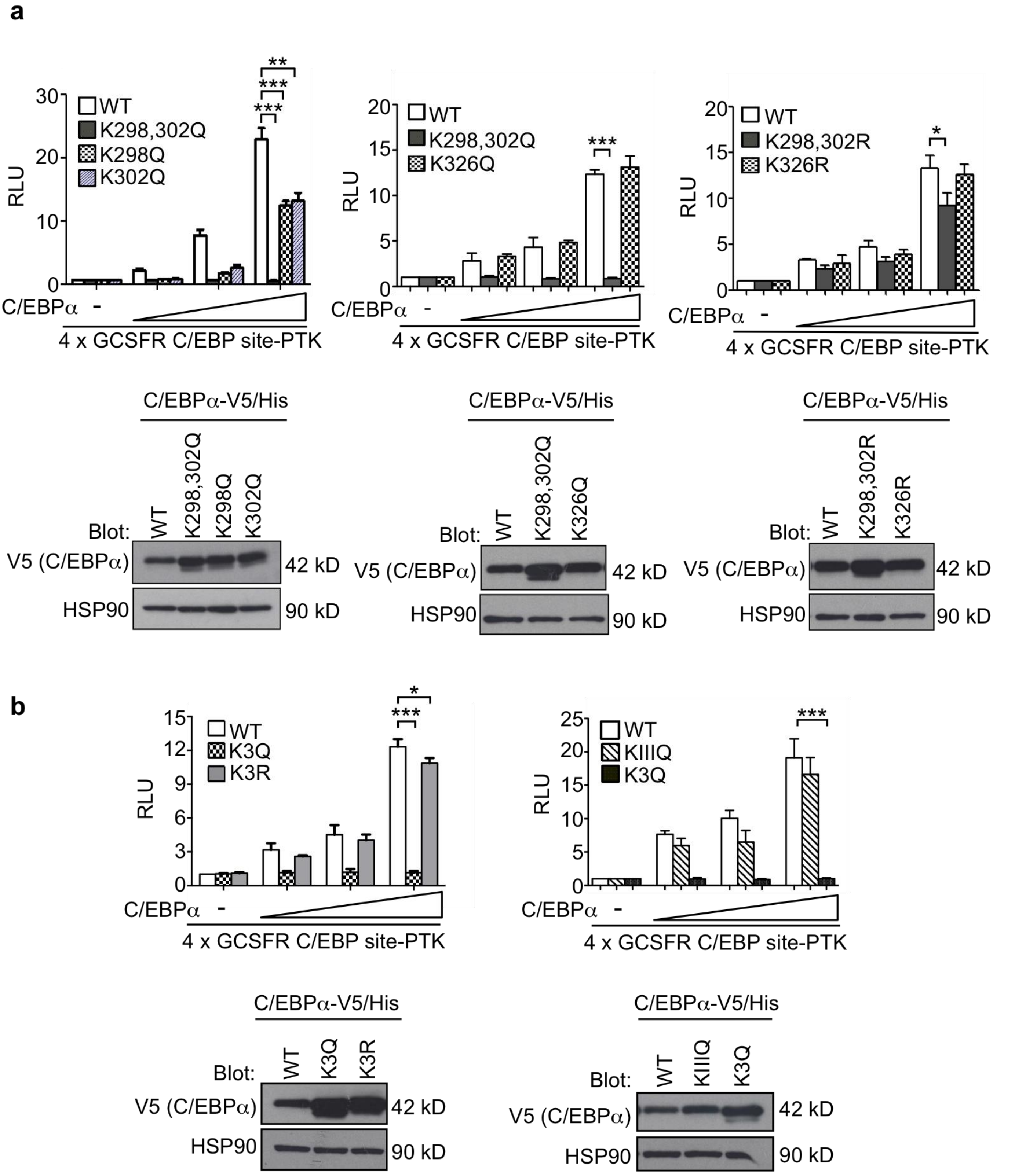

Supplementary Figure 4 (cont.)

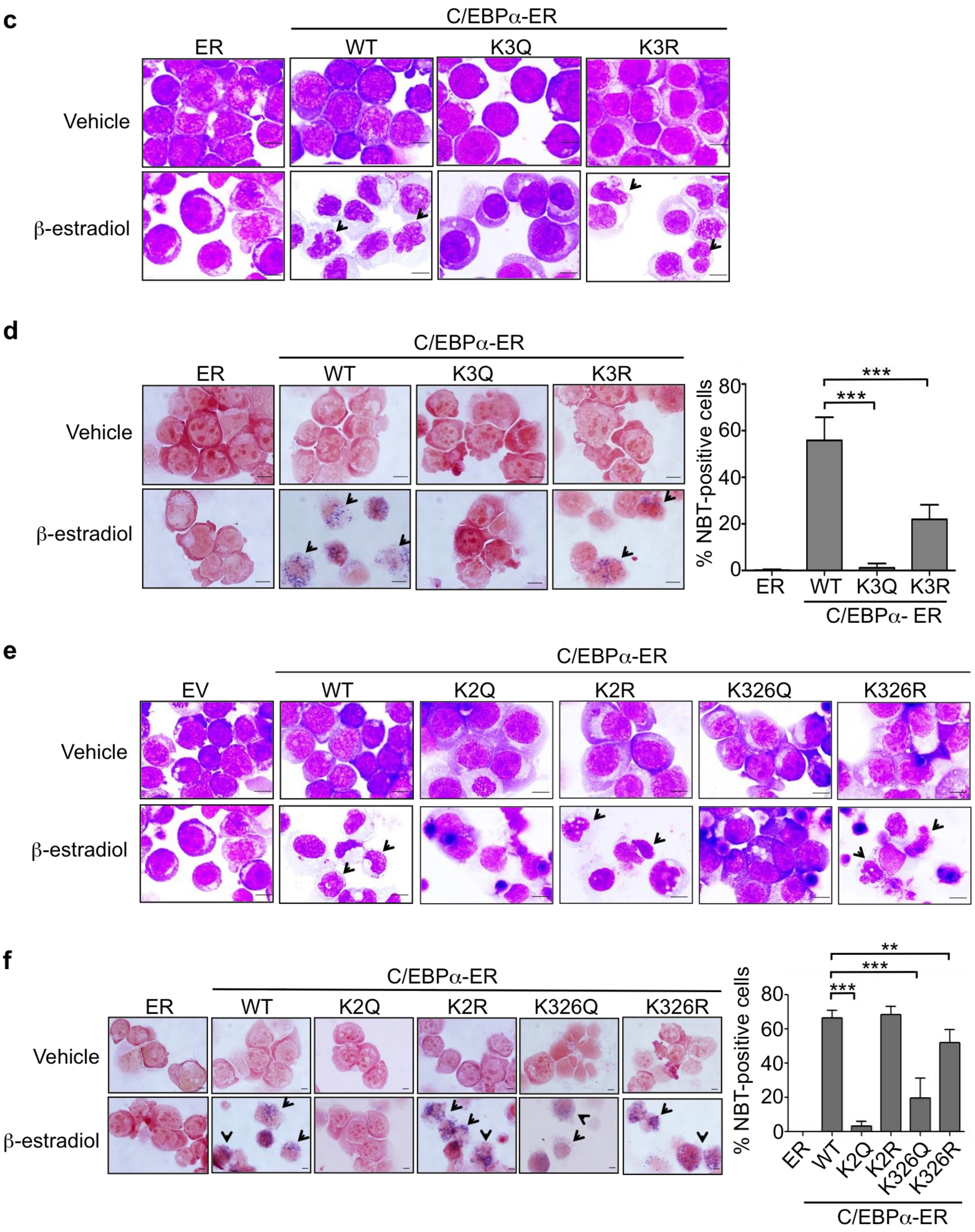

Supplementary Figure 4 (cont.)

g

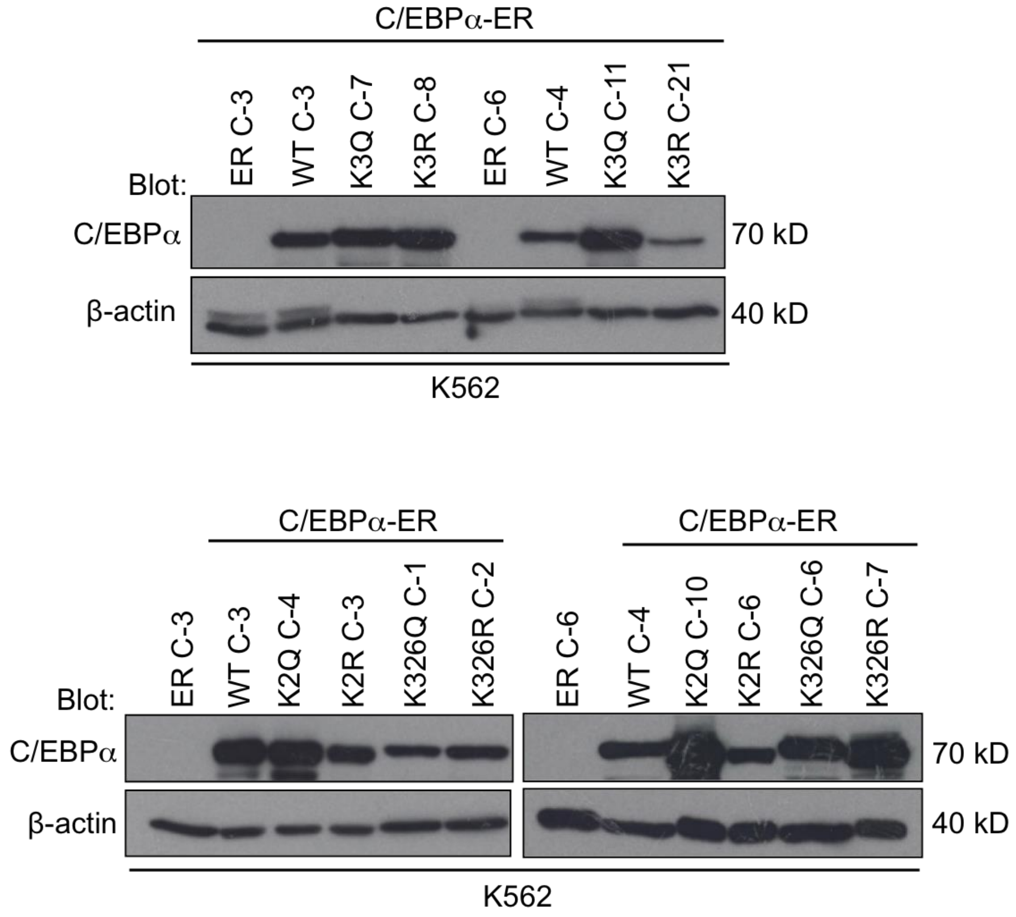

Supplementary Figure 4 (cont.)

h

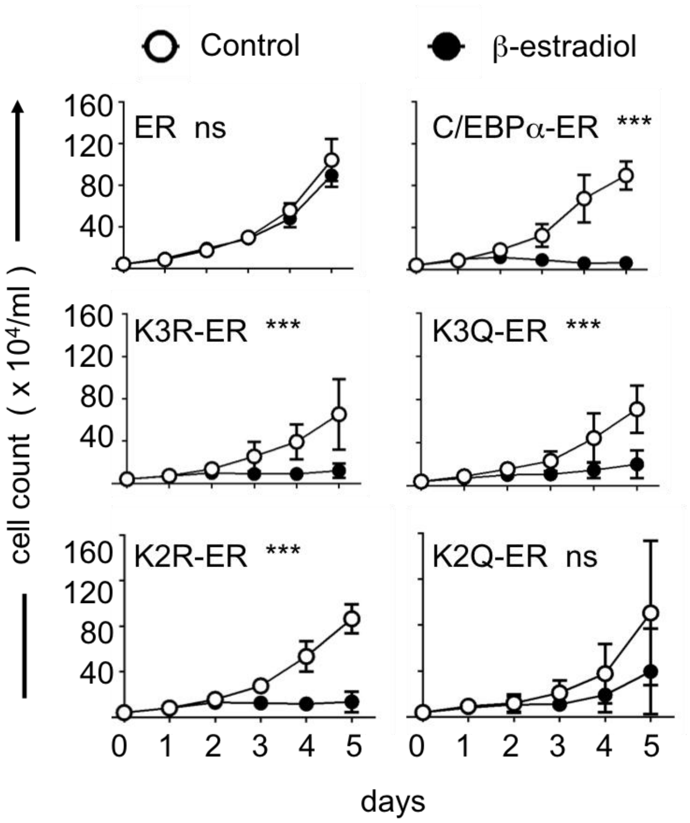

i

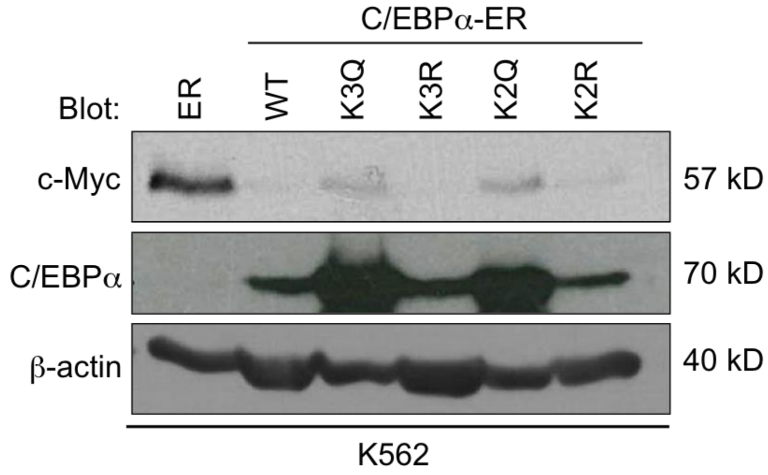

j

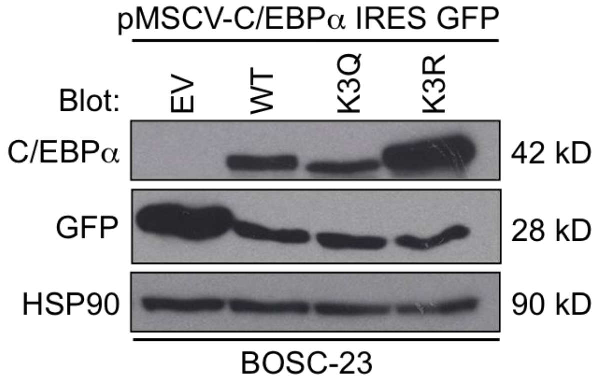

Supplementary Figure 4. **Acetylation mimetic mutants of C/EBPα lack differentiation potential and slow cell growth.**

(a, b) Luciferase assays for C/EBPα non-acetylated and acetylation mimetic mutants. Cells were transiently transfected with pcDNA6 C/EBPα WT or mutants in a dose-dependent manner. Western blots demonstrating expression of WT and mutant C/EBPα proteins are shown below the luciferase graphs. Luciferase activity was measured in duplicate for each experiment and data are shown as mean±s.d. (*N*=3).

(c, d, e, f) Cytospins shown were stained with Wright-Giemsa or NBT dye. Original magnification x100, scale bars indicate 10 μm. At least 100 cells were counted for each line for data quantification.

(c, e) Morphological differentiation of C/EBPα-ER expressing stable lines induced with 5 μM β-estradiol for 4 days. Arrows indicate polymorphonuclear morphology of granulocytes.

(d, f) C/EBPα-ER expressing stable lines were induced with 5 μM β-estradiol for 4 days and analyzed for NBT reduction. Small purple dots were indicative of NBT activity, some examples noted by arrows. Quantified data are presented on the right. Data are mean±s.d. [*N*≥3(d); *N*≥5(f)].

(g) Western blot showing expression of various K562 stably transfected clones used in this study.

(h) Acetylation mimetic mutant K2Q showed partial cell growth inhibition. Cell numbers are measured for K562 cells stably transfected with indicated C/EBPα-ER fusion proteins in the absence (o) or presence (•) of β-estradiol. ER indicates estrogen receptor vector control. The error bars represent standard deviation from 2 independent clones for each C/EBPα-ER fusion protein (*N*=6).

(i) Acetylation mimetic mutant showed intermediate downregulation of c-Myc. Western blot showing protein expression of c-Myc, C/EBPα and β-actin from various K562 stably transfected clones.

(j) Expression of retroviral constructs pMIG EV, C/EBPα WT, K3Q, and K3R were confirmed by Western blot in BOSC-23 cells. These constructs were used to transduce LSK cells from C/EBPα$^{\Delta/\Delta}$ mice. *$P < 0.05$, **$P< 0.01$ and ***$P< 0.001$; Student's unpaired t-test (a, b, d, and f); two-way ANOVA test (h).

Supplementary Figure 5

a

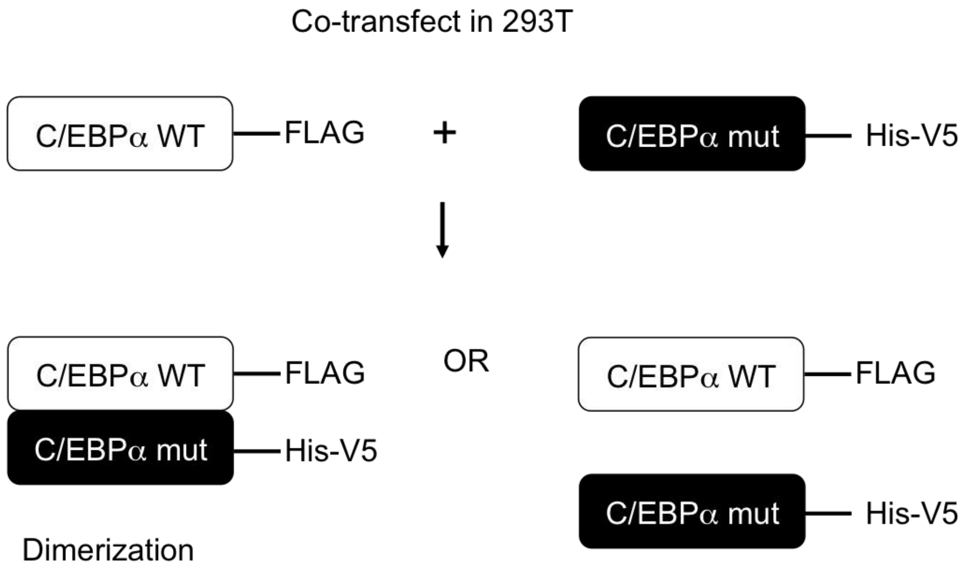

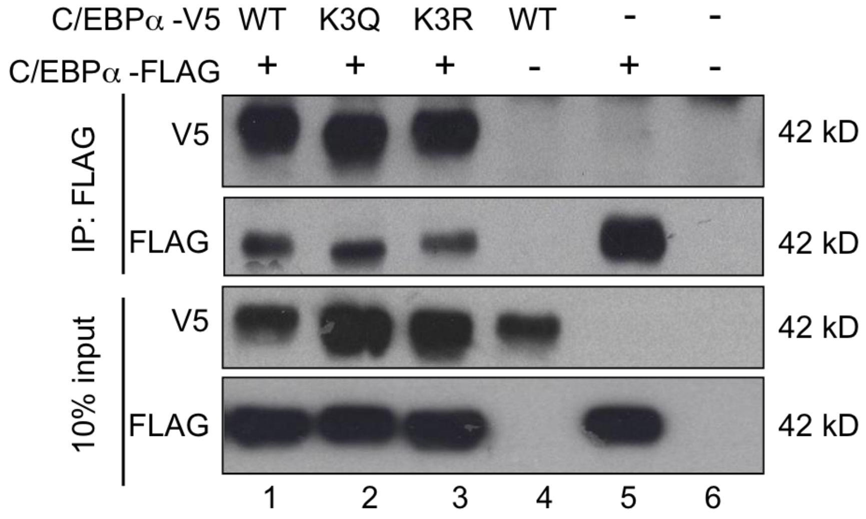

Supplementary Figure 5 (cont.)

b

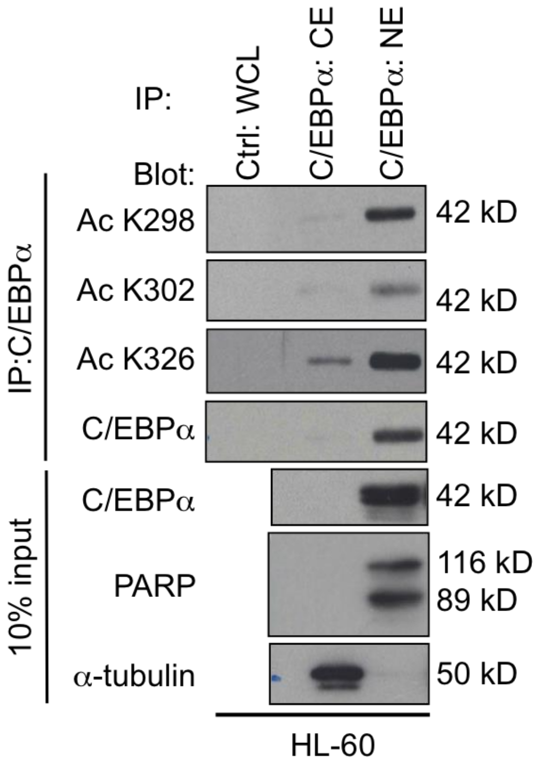

c

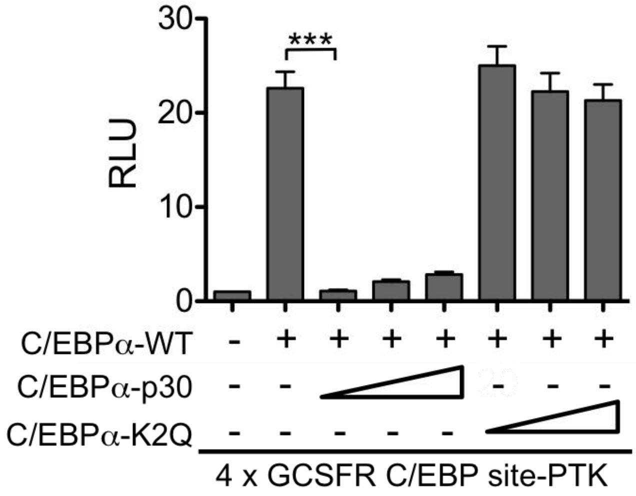

d

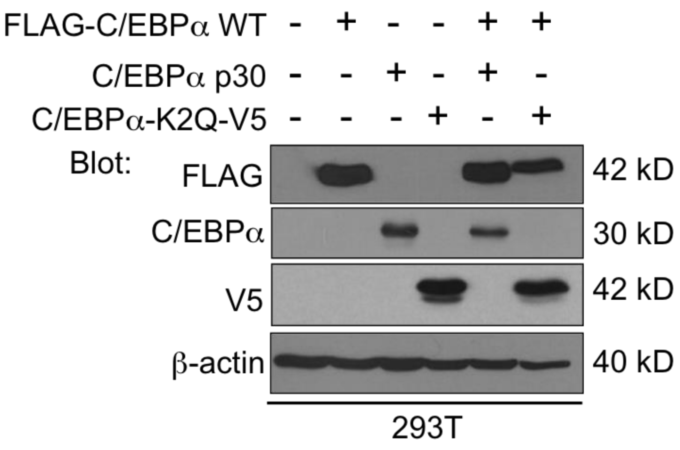

Supplementary Figure 5 (cont.)

e

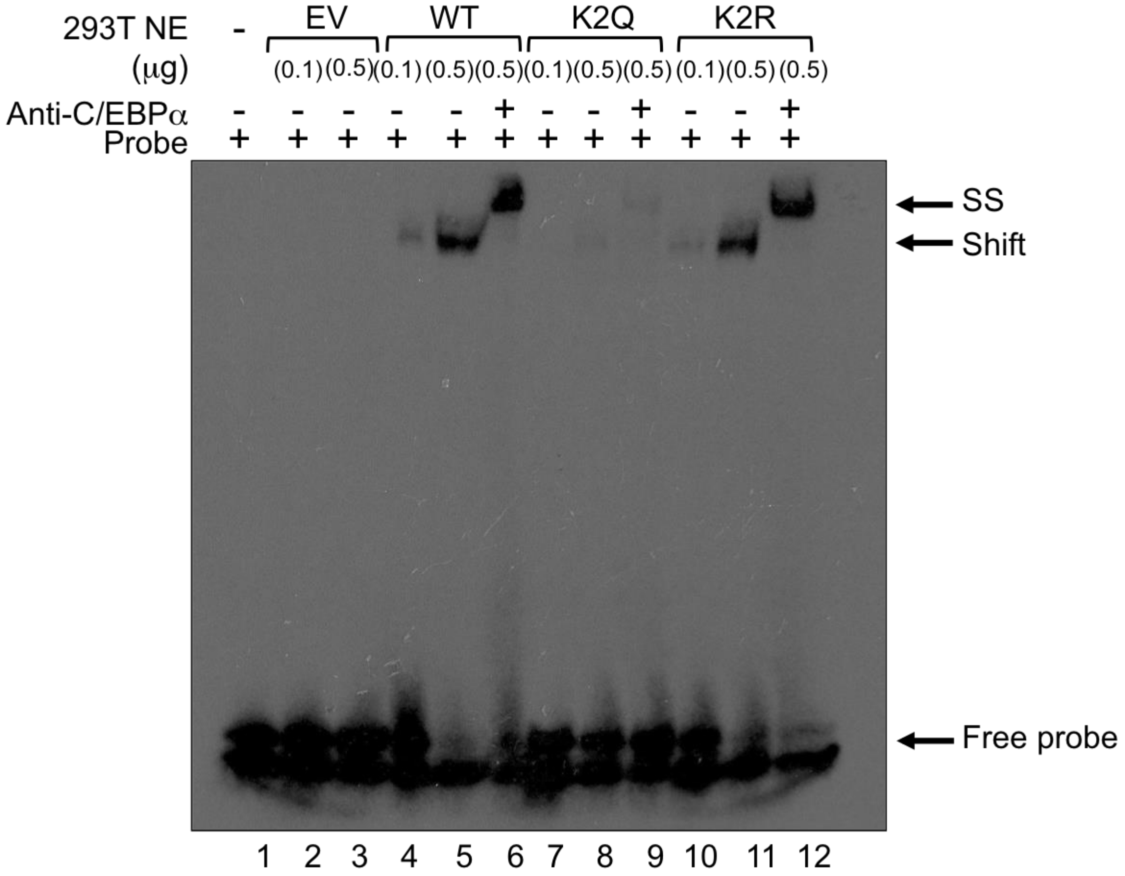

f

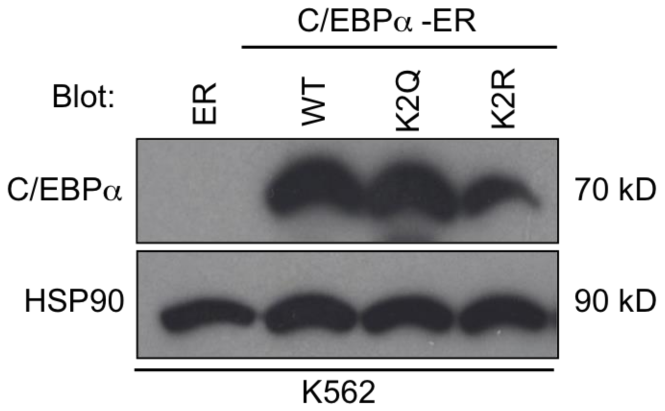

g

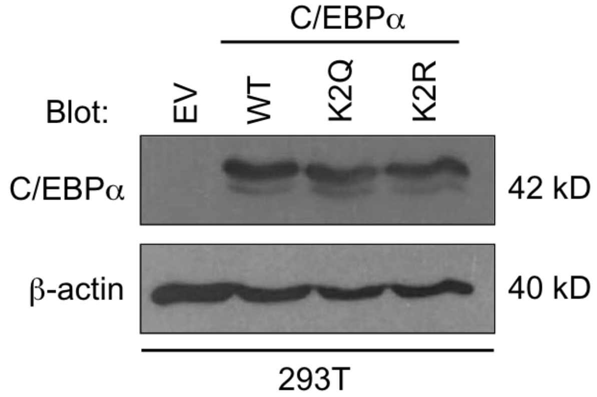

Supplementary Figure 5. Acetylation attenuates DNA binding but does not affect homodimerization, subcellular localization of C/EBPα and lacks dominant negative function over C/EBPα WT protein

(a) Acetylation does not affect homodimerization activity. 293T cells were transiently co-transfected with C/EBPα-WT, K3Q, or K3R with V5 tag along with FLAG-tagged C/EBPα-WT. Co-immunoprecipitation was performed using FLAG M2 beads. Lanes 4, 5, and 6 serve as controls.

(b) Acetylation does not alter nuclear localization of C/EBPα. HL-60 cells were fractionated into nuclear (NE) and cytosolic (CE) fractions, and C/EBPα was immunoprecipitated with anti-C/EBPα antibody and immunoblotted with acetyl-C/EBPα antibodies. Nuclear and cytosolic fractions were blotted for PARP and α-tubulin to ensure efficacy of cell fractionation.

(c, d) C/EBPα K2Q lacks dominant negative function over C/EBPα WT unlike C/EBPα p30 isoform. 293T cells were transiently transfected with C/EBPα WT (1 ng) with C/EBPα p30 or K2Q (K298, K302Q) in a dose-dependent manner (1 ng, 10 ng, 20 ng) with p(CEBP)4TK promoter , pRL-null. Luciferase assays were done as reported in Figure 1. Data represent mean±s.d (*N*=3). ****P*< 0.001; Student's unpaired t-test. Western blots demonstrating expression of C/EBPα proteins were shown in (d).

(e) C/EBPα acetylation mimetic (K2Q) is shown to have significantly reduced DNA binding affinity. EMSA was performed using equal amounts of nuclear extracts (NE: 0.1 µg and 0.5 µg) from 293T cells transiently transfected with C/EBPα WT or mutant constructs without ER tag were used for EMSA. Shift indicates C/EBPα complex and supershift showed C/EBPα antibody complex. With an increase in the amount of NE, higher binding of probe to C/EBPα complex was observed in C/EBPα WT (lane 4, 5) and C/EBPα K2R (lane 10, 11) with no binding for EV (lane 2, 3) and minimal for K2Q (lane 7, 8).

(f, g) Western blot of nuclear protein extracts from K562 stable lines treated with 5 µM β-estradiol (f) and transiently transfected 293T cells (g). The amount of extract used in Figure 5c, and Supplementary Figure 5e were adjusted according to the Western blot analysis in order to use equal amount of nuclear extracts for EMSA.

Supplementary Figure 6

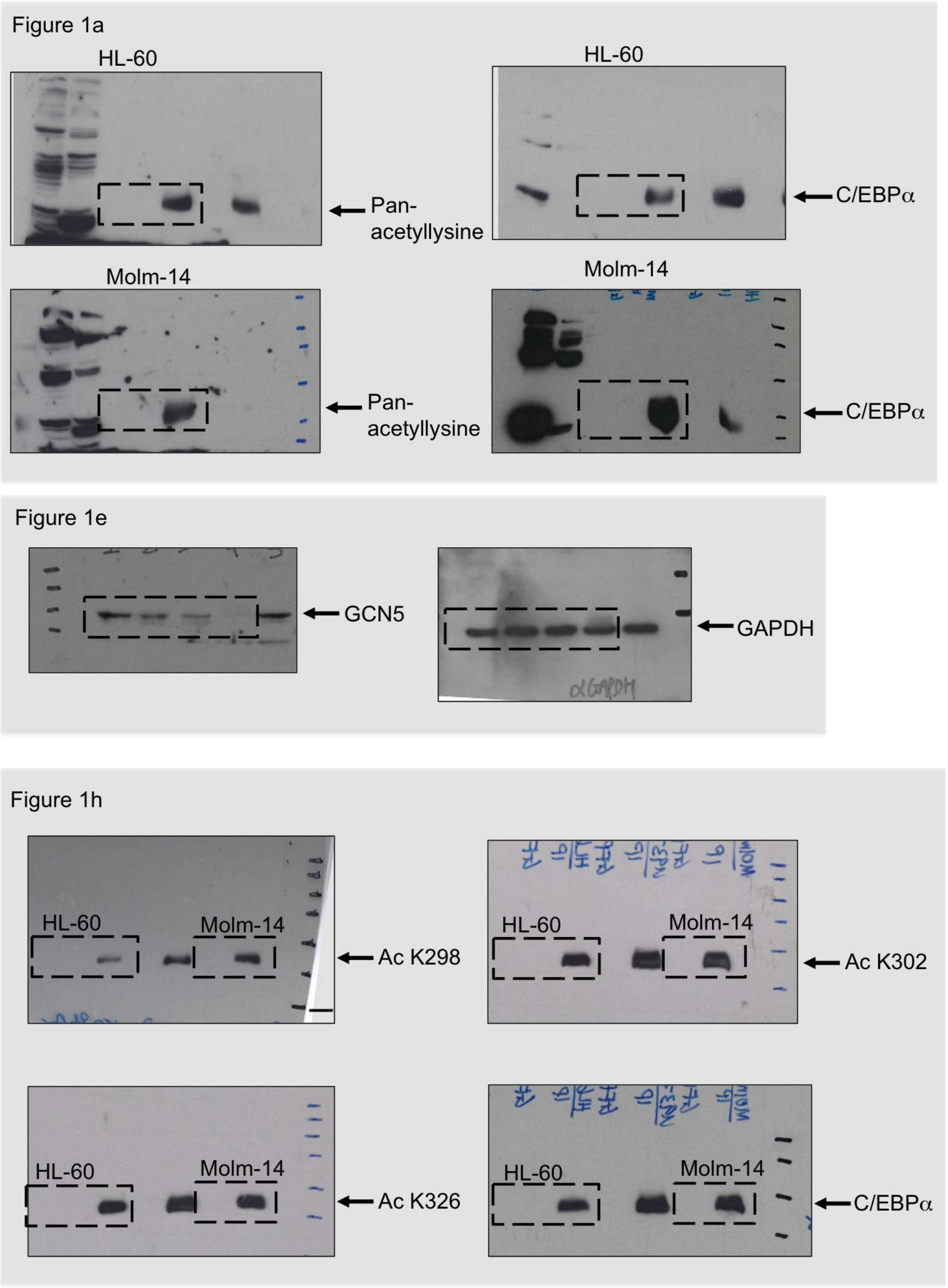

Supplementary Figure 6. Full scans of Western blot data shown in Figure 1.

Rectangles delimit cropped areas used in the indicated panels in Figure 1.

Supplementary Figure 7

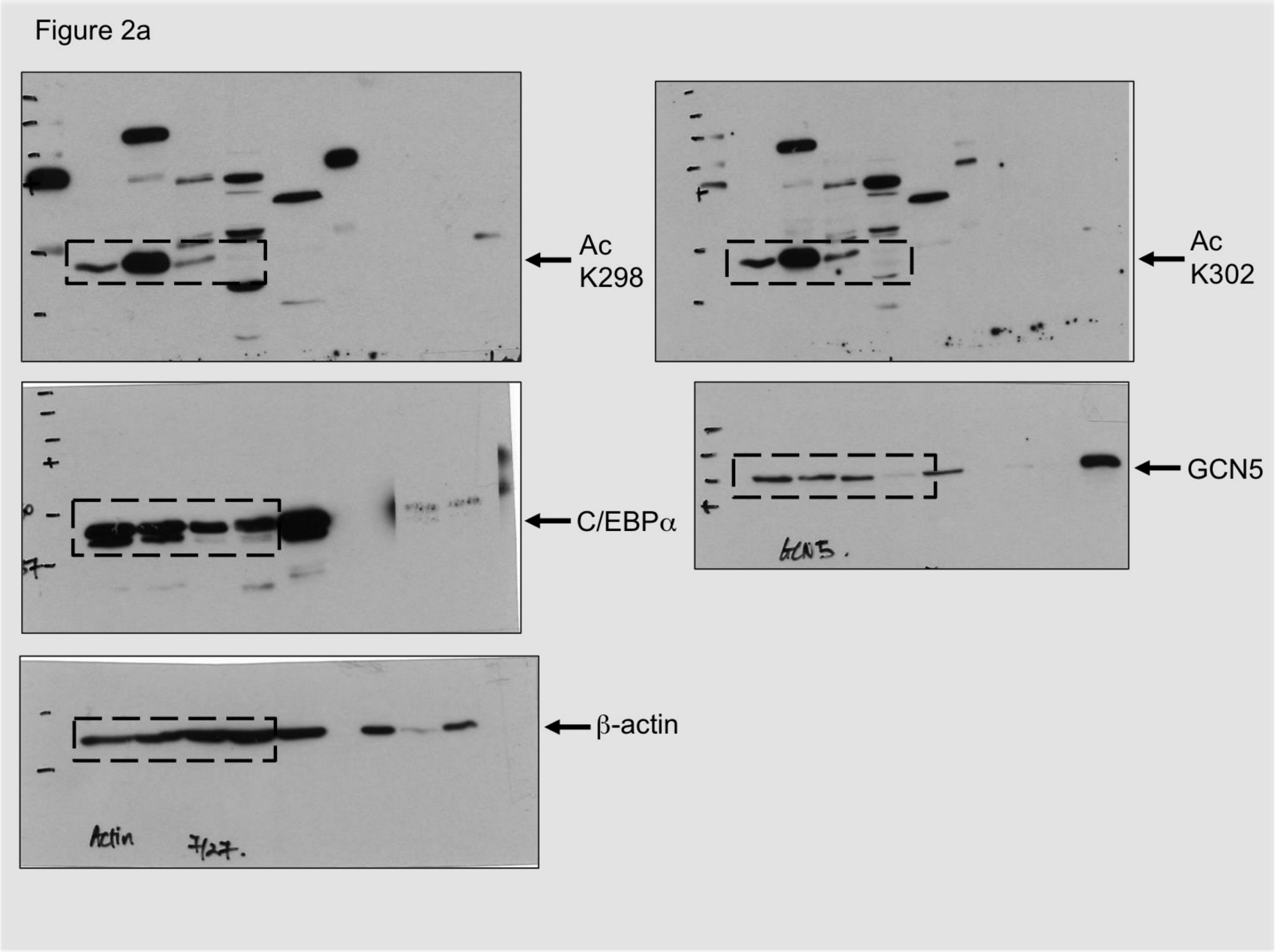

Supplementary Figure 7 (cont.)

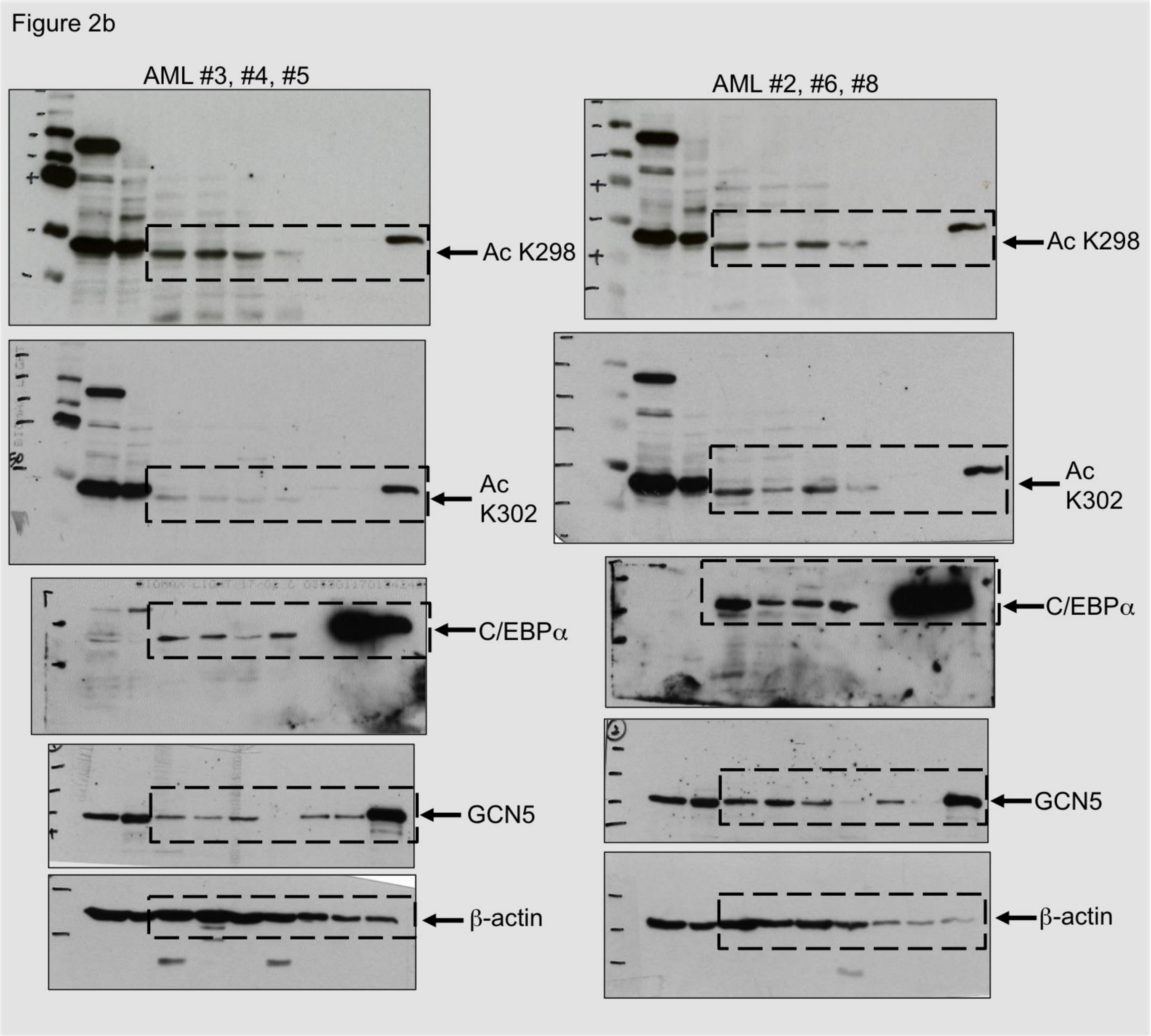

Supplementary Figure 7. Full scans of Western blot data shown in Figure 2.

Rectangles delimit cropped areas used in the indicated panels in Figure 2.

Supplementary Figure 8

Figure 5a

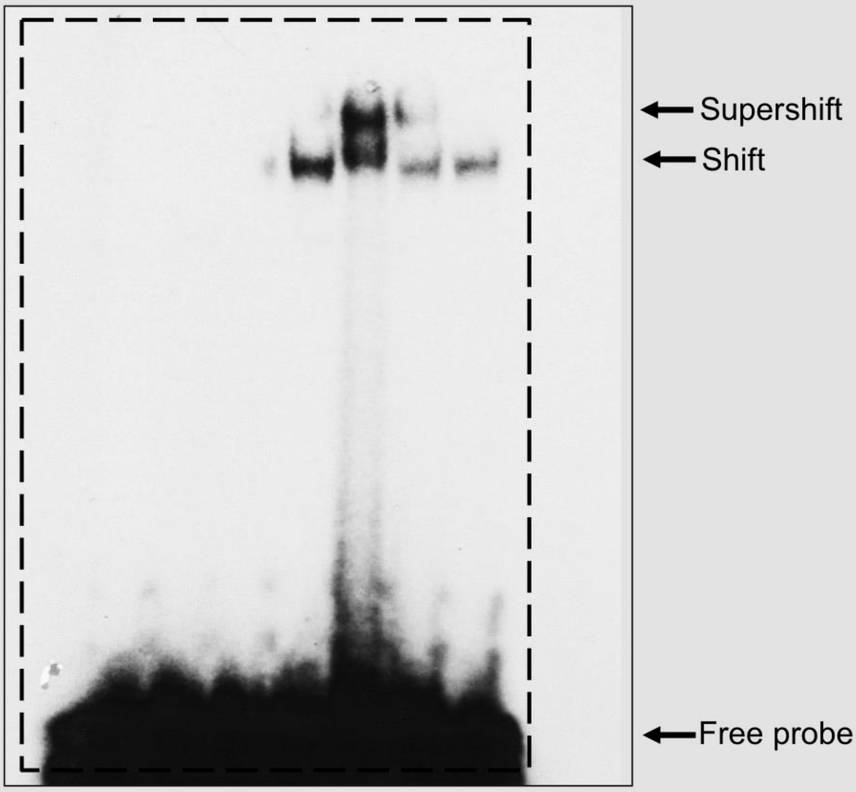

Figure 5b

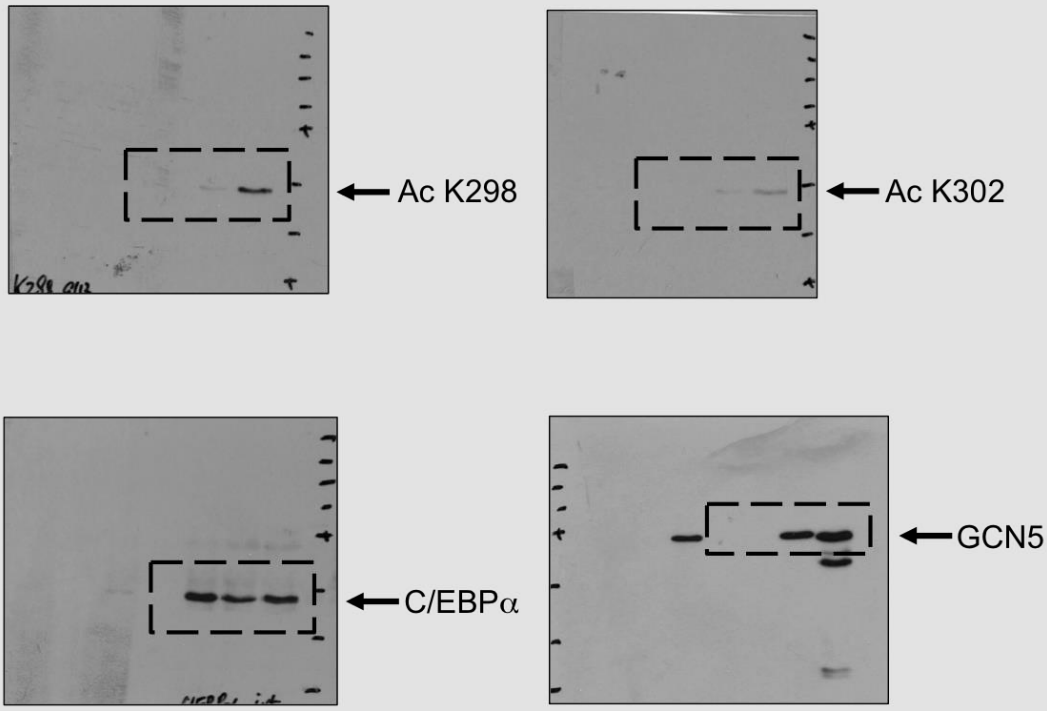

Supplementary Figure 8 (cont.)

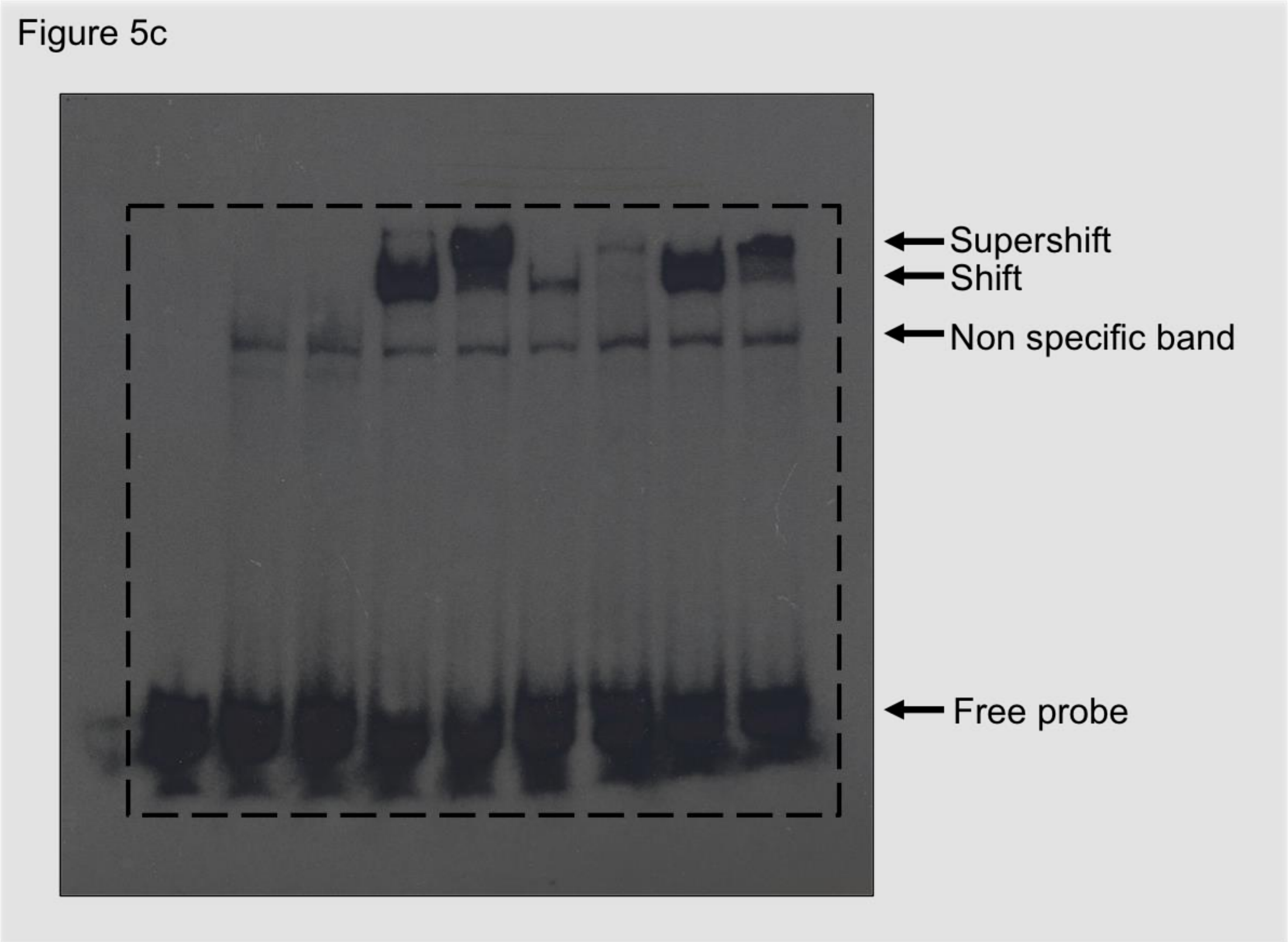

Supplementary Figure 8. Full scans of Western blot and EMSA data shown in Figure 5.

Rectangles delimit cropped areas used in the indicated panels in Figure 5.

Supplementary Figure 9

Supplementary Figure 1b

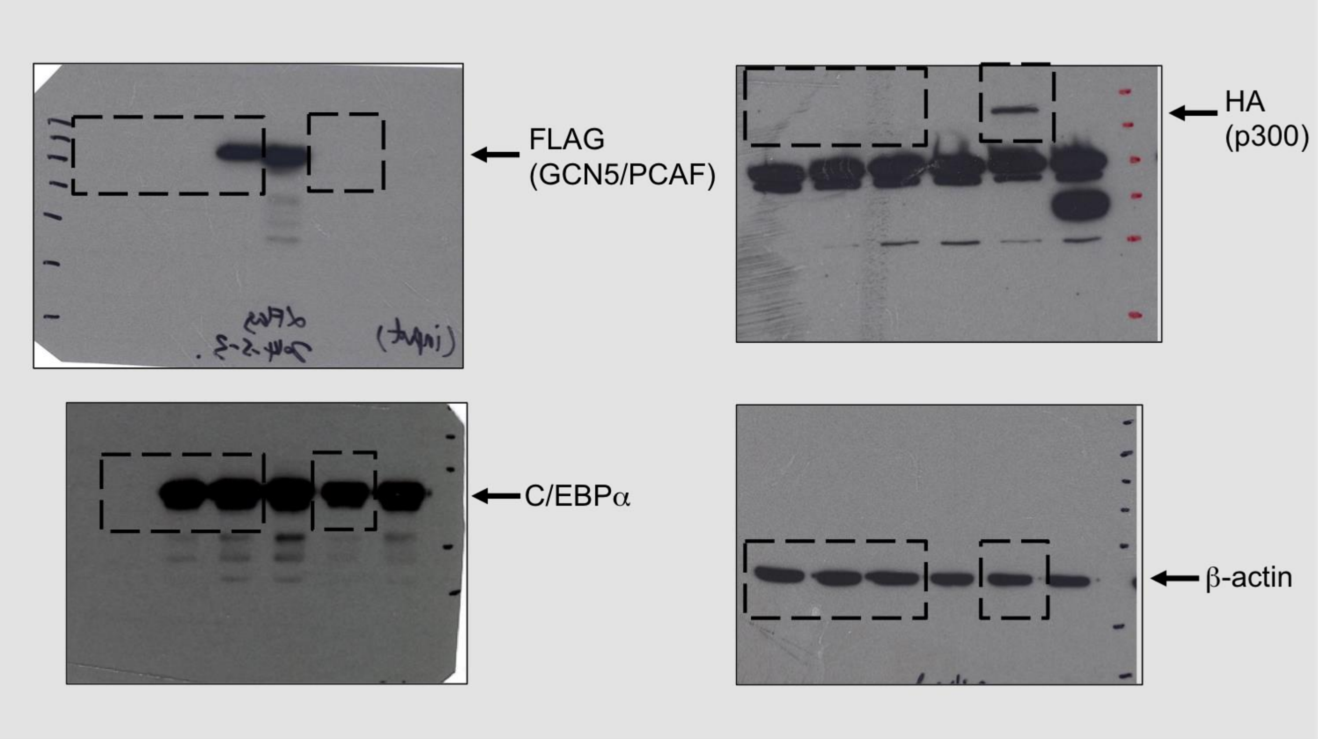

Supplementary Figure 1c

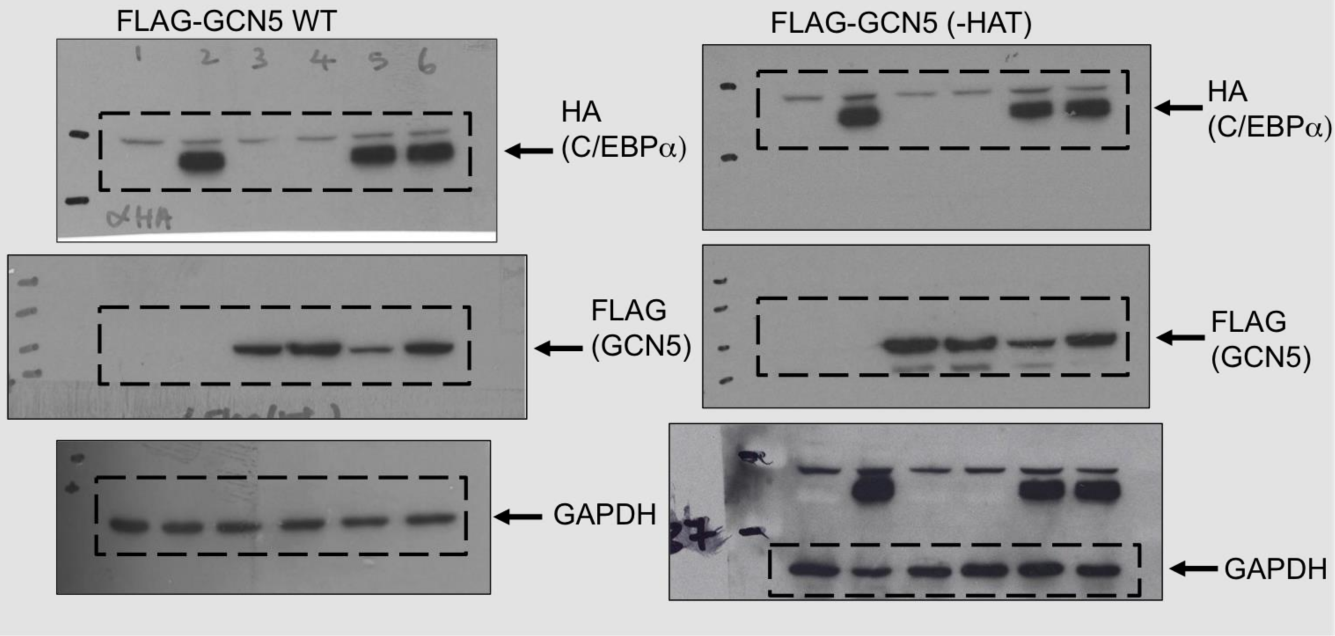

Supplementary Figure 9 (cont.)

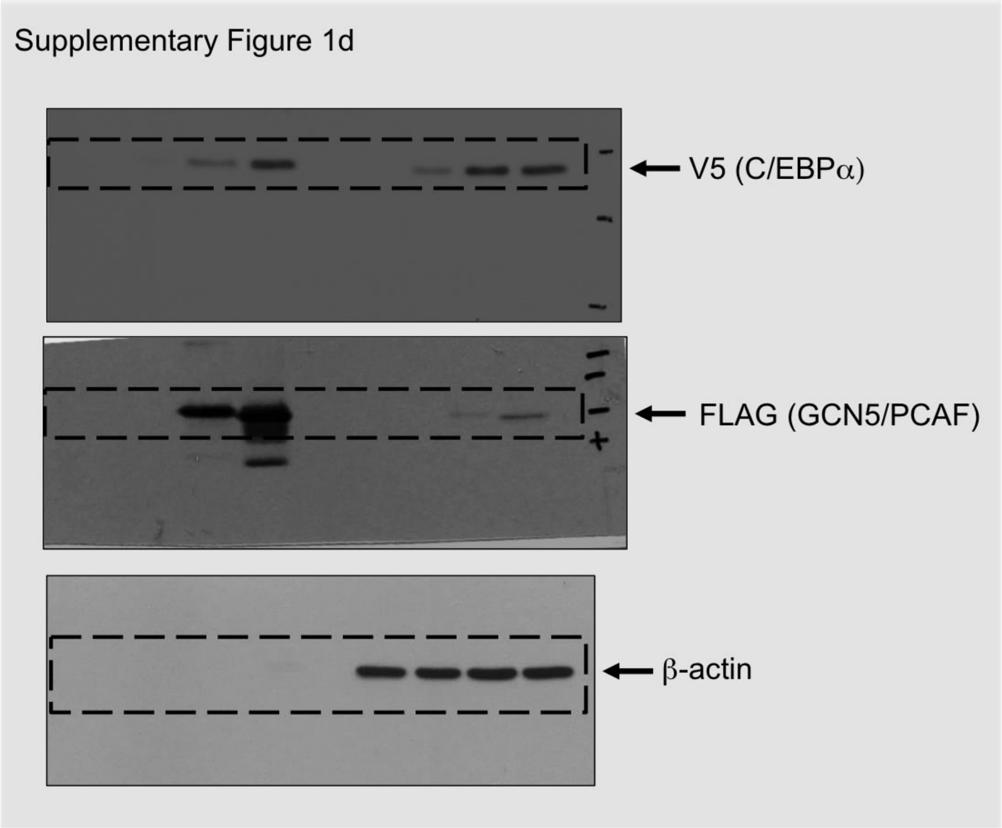

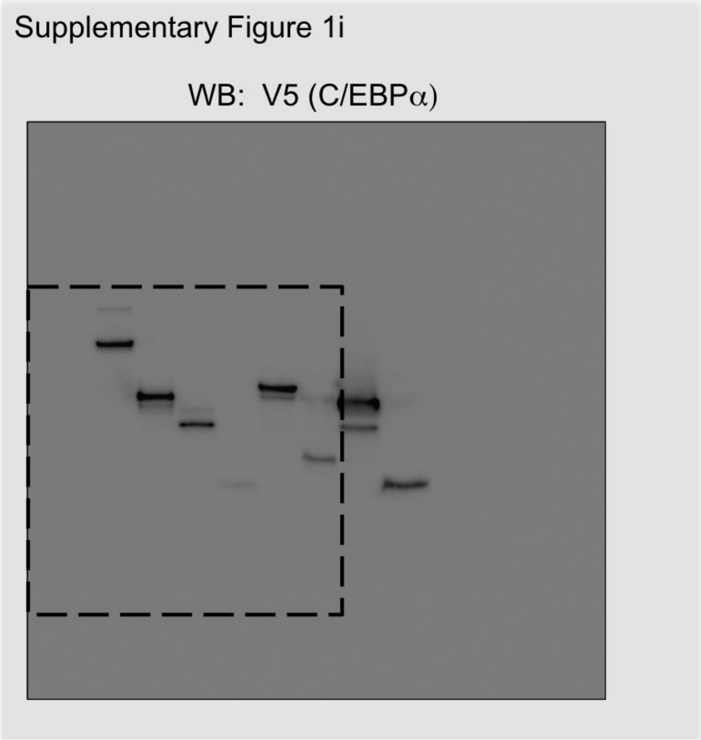

Supplementary Figure 9 (cont.)

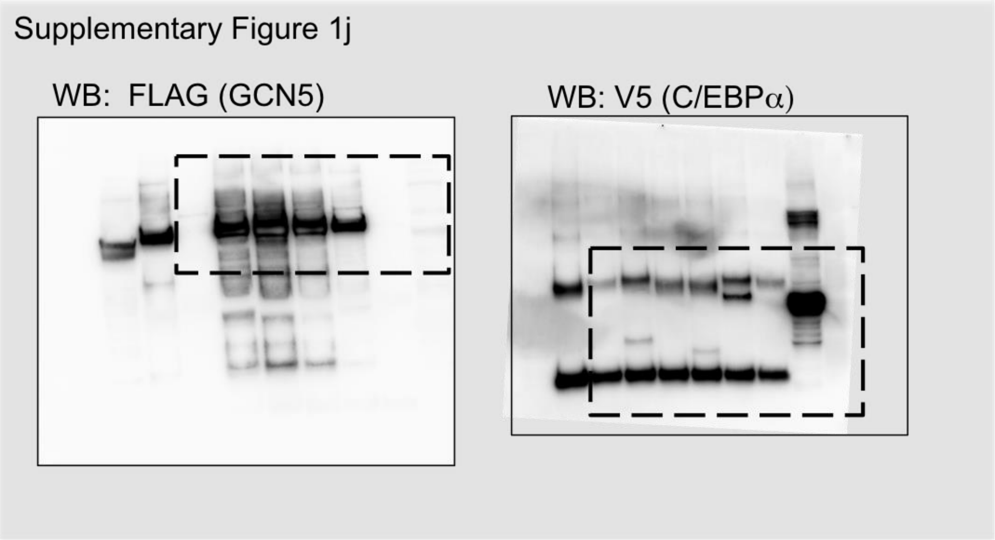

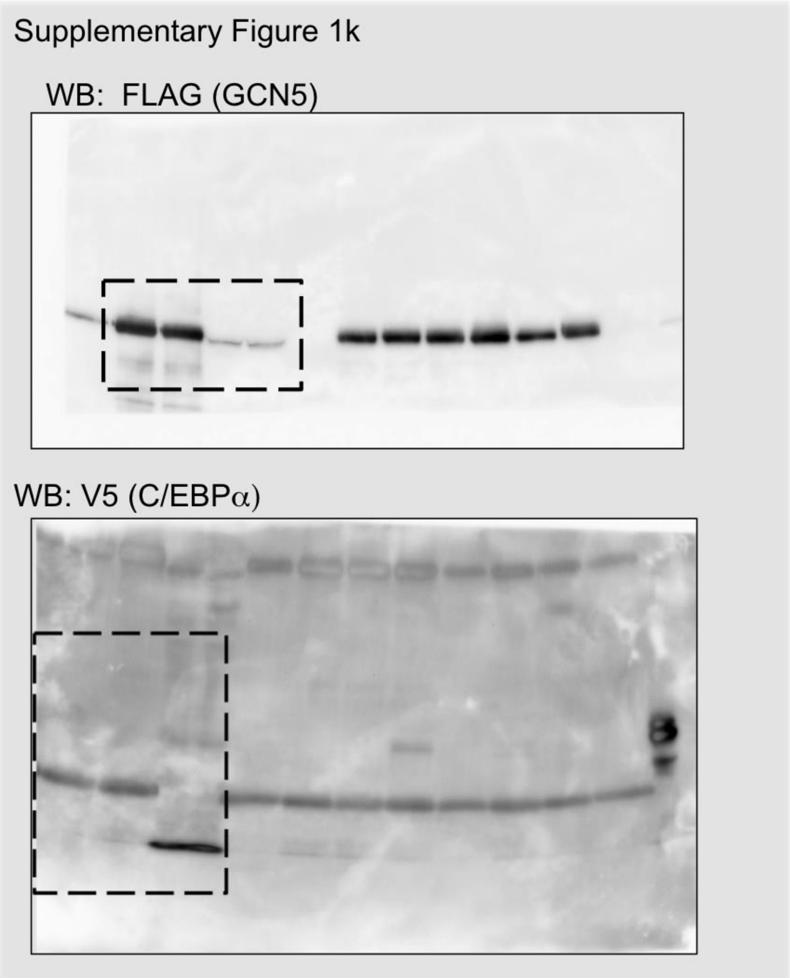

Supplementary Figure 9 (cont.)

Supplementary Figure 1m

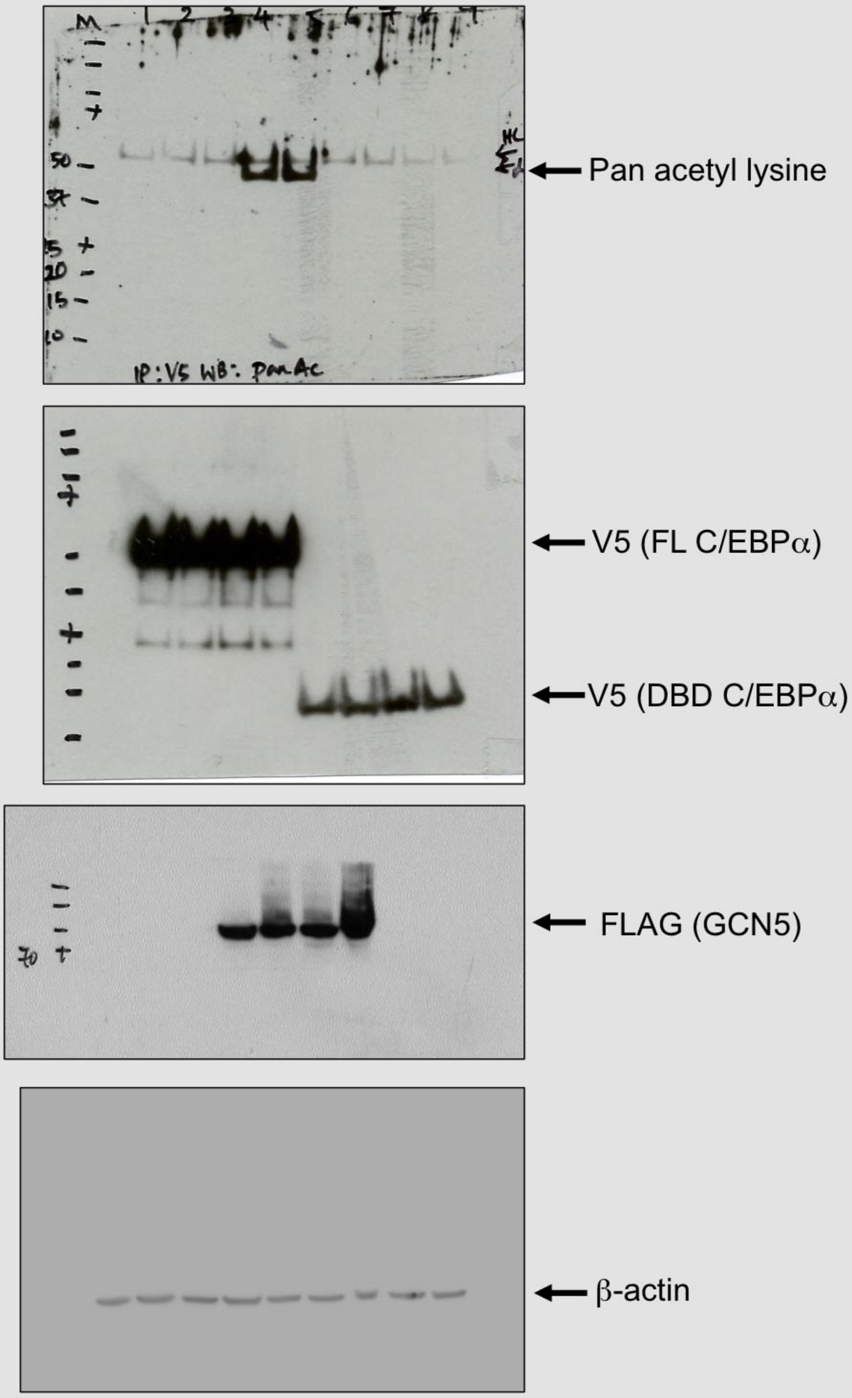

Supplementary Figure 9 (cont.)

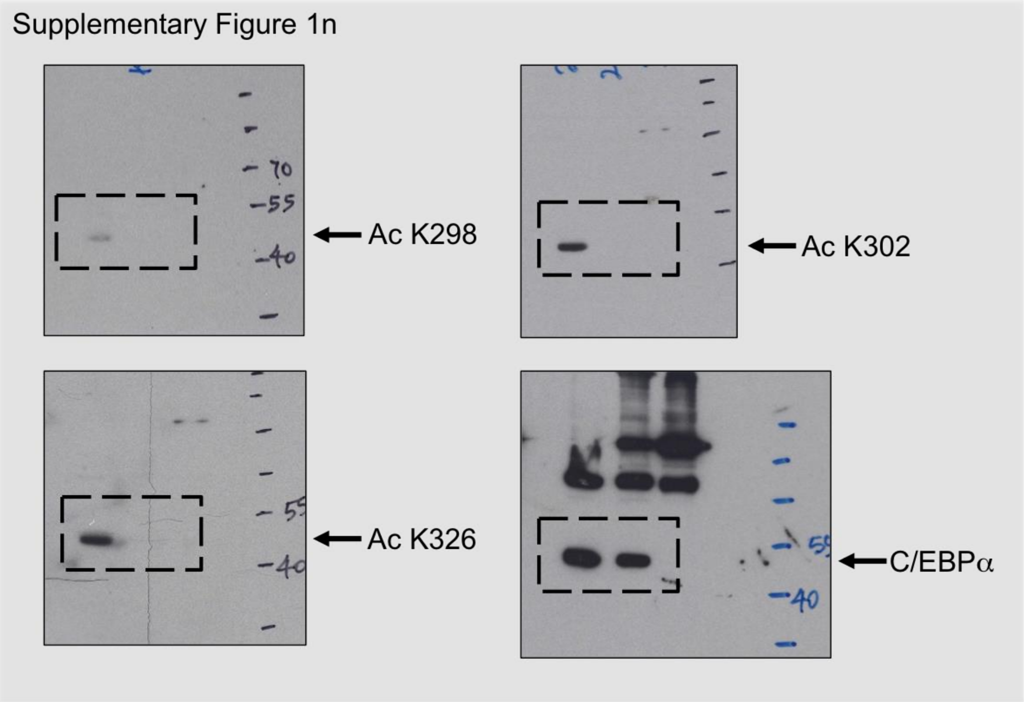

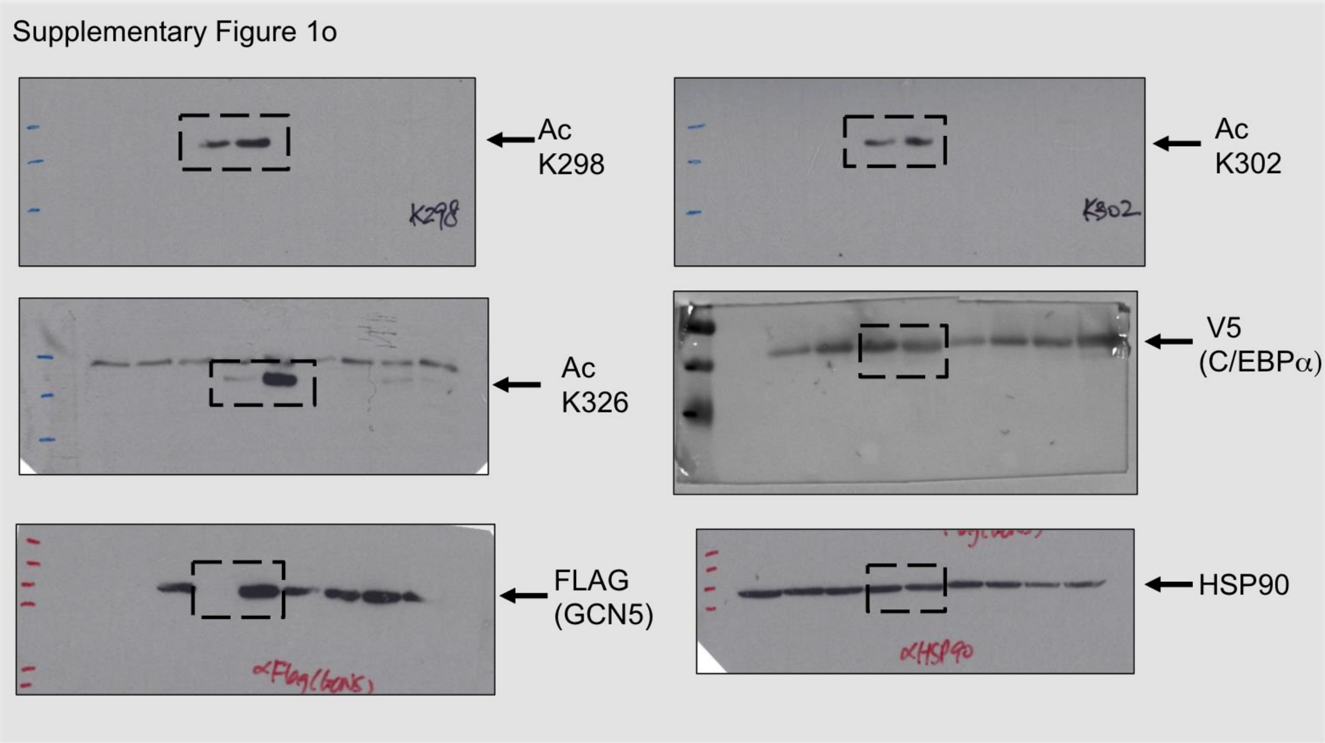

Supplementary Figure 9. Full scans of Western blot shown in Supplementary Figure 1.

Rectangles delimit cropped areas used in the indicated panels in Supplementary Figure 1.

Supplementary Figure 10

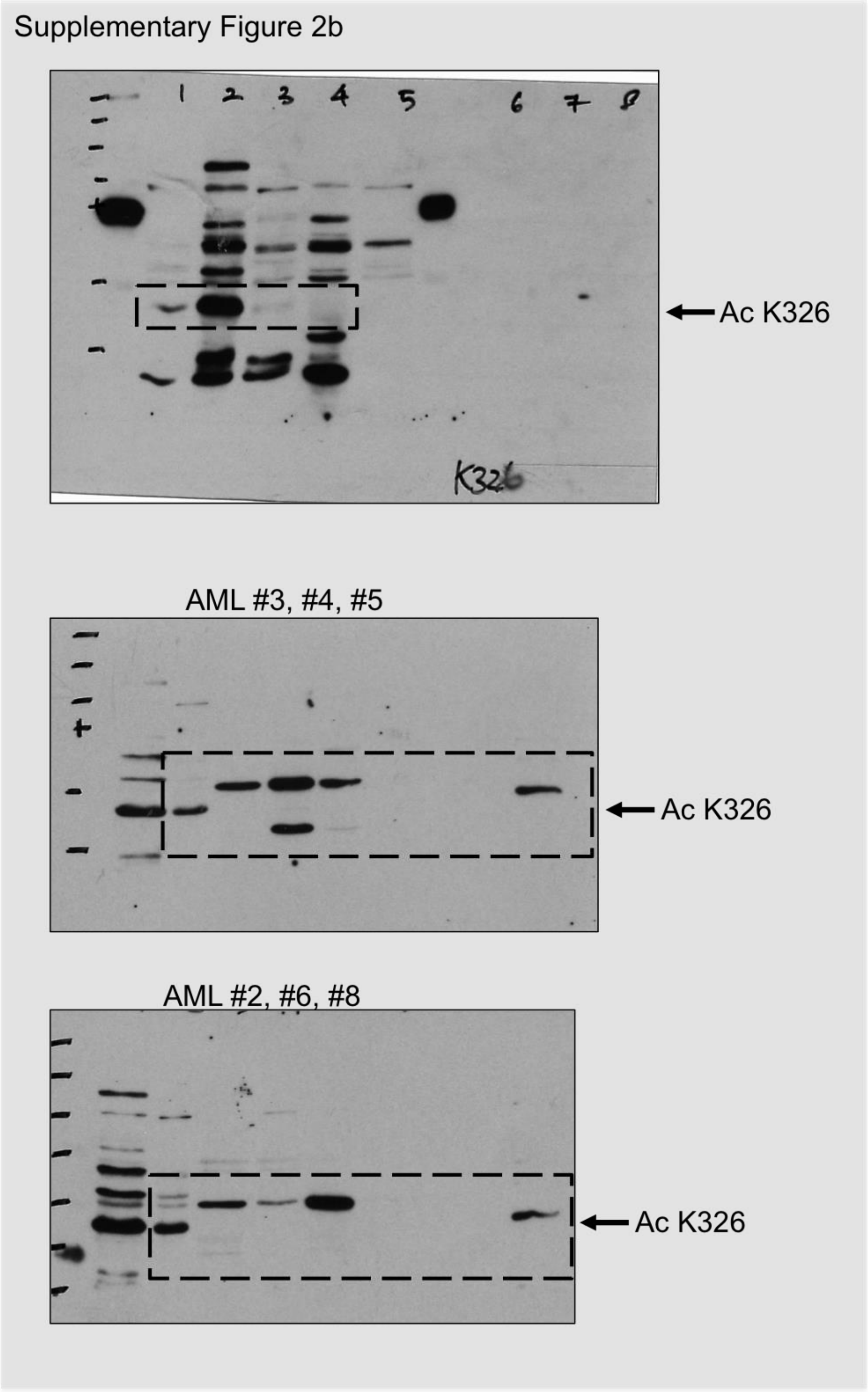

Supplementary Figure 10. Full scans of Western blot shown in Supplementary Figure 2.

Rectangles delimit cropped areas used in the indicated panels in Supplementary Figure 2

Supplementary Figure 11

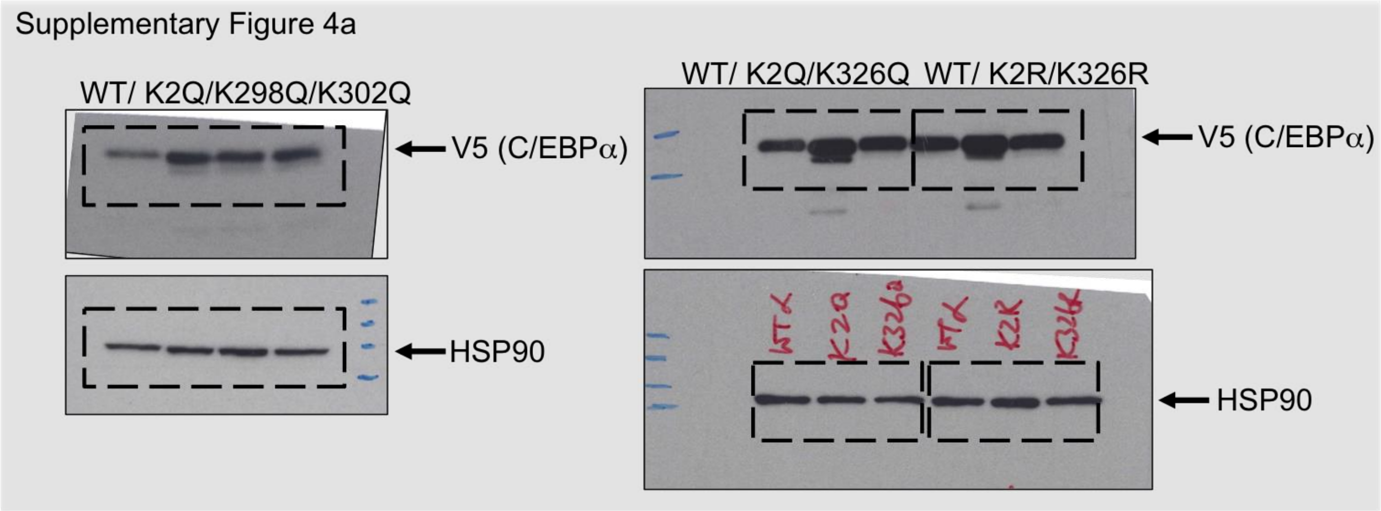

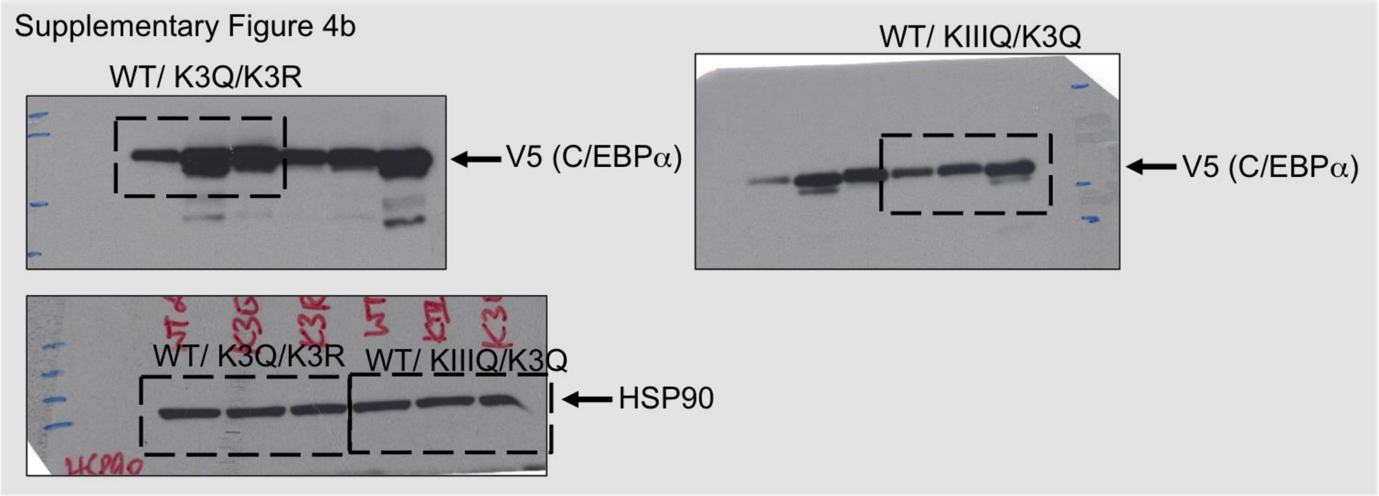

Supplementary Figure 11 (cont.)

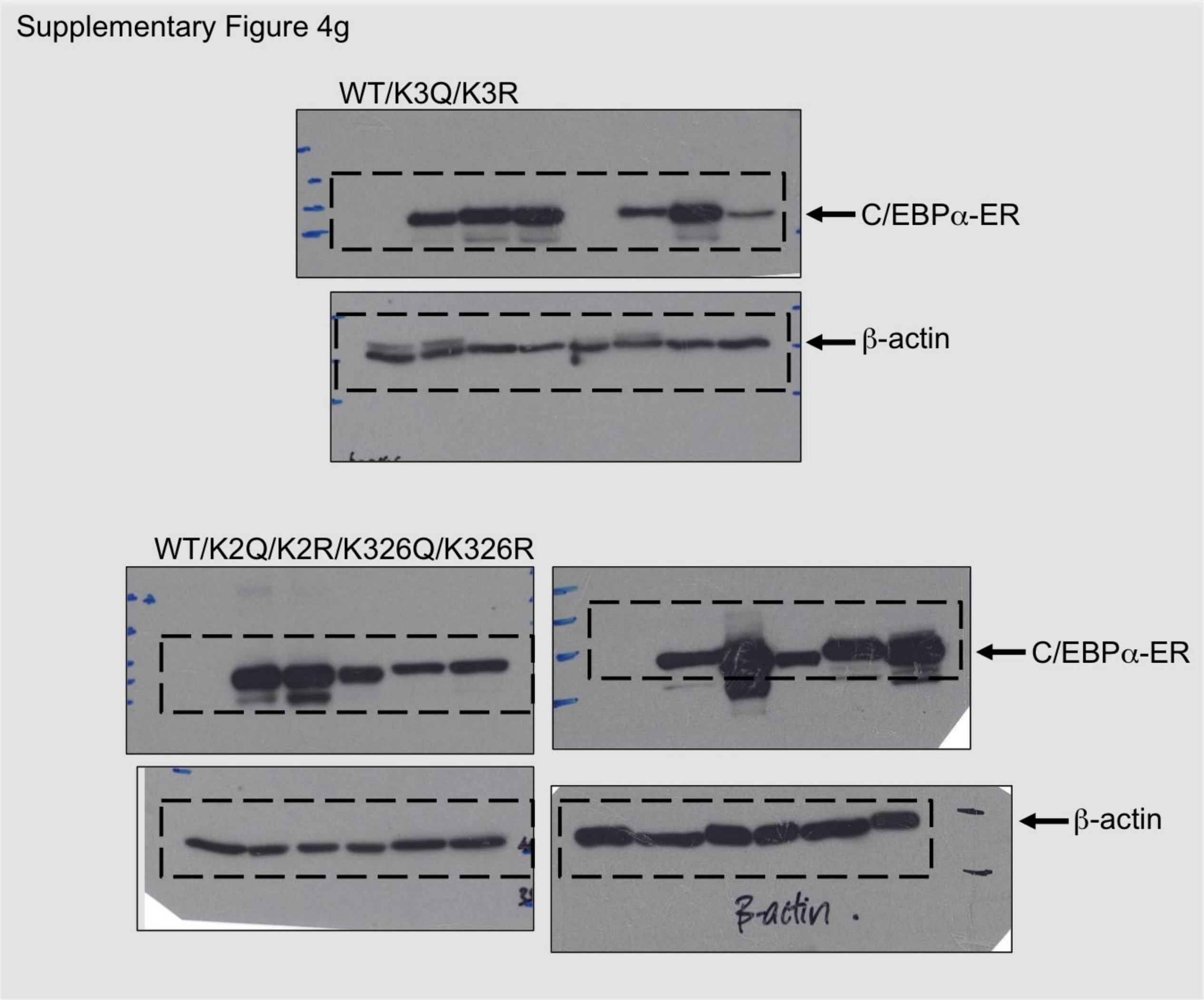

Supplementary Figure 11 (cont.)

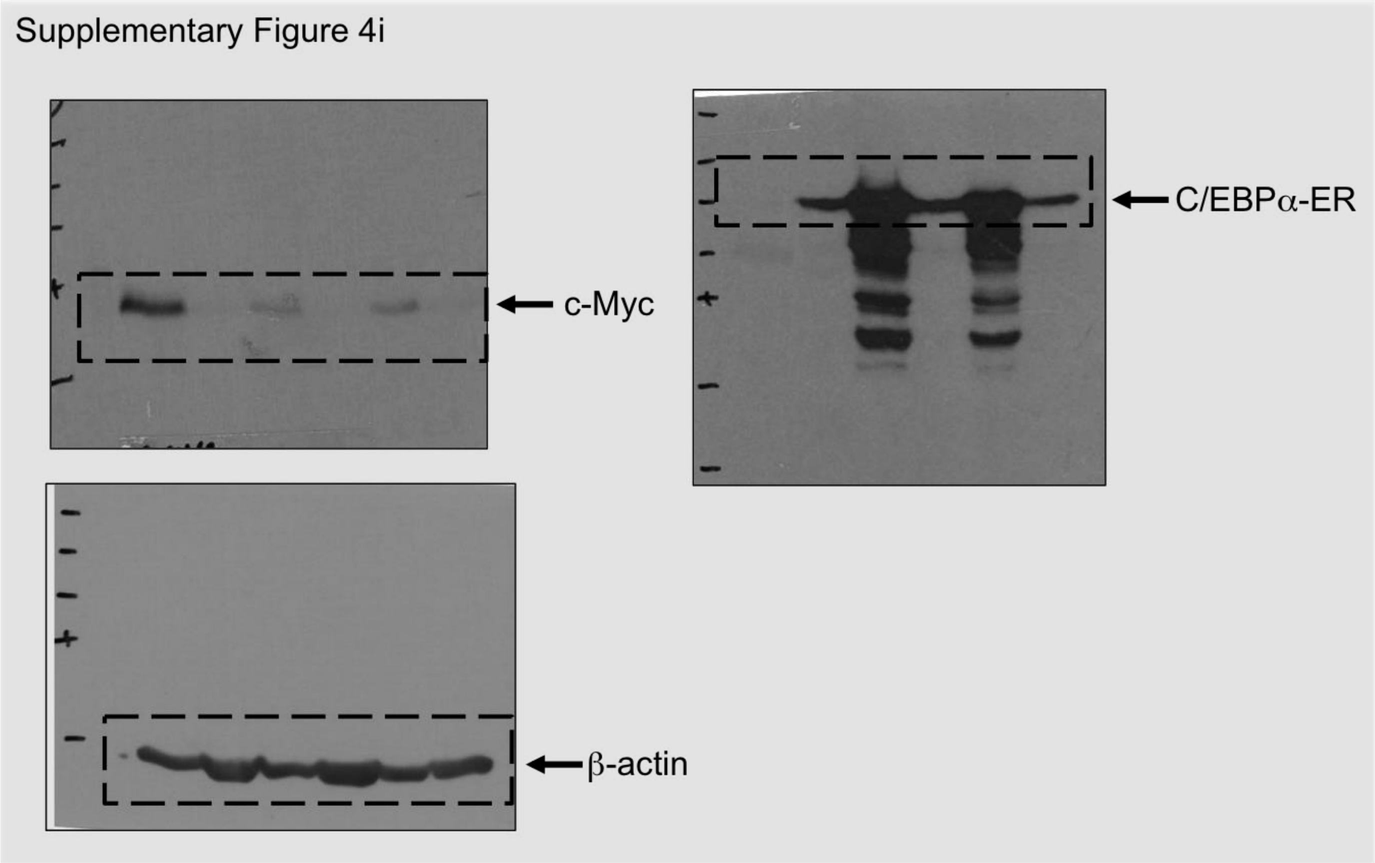

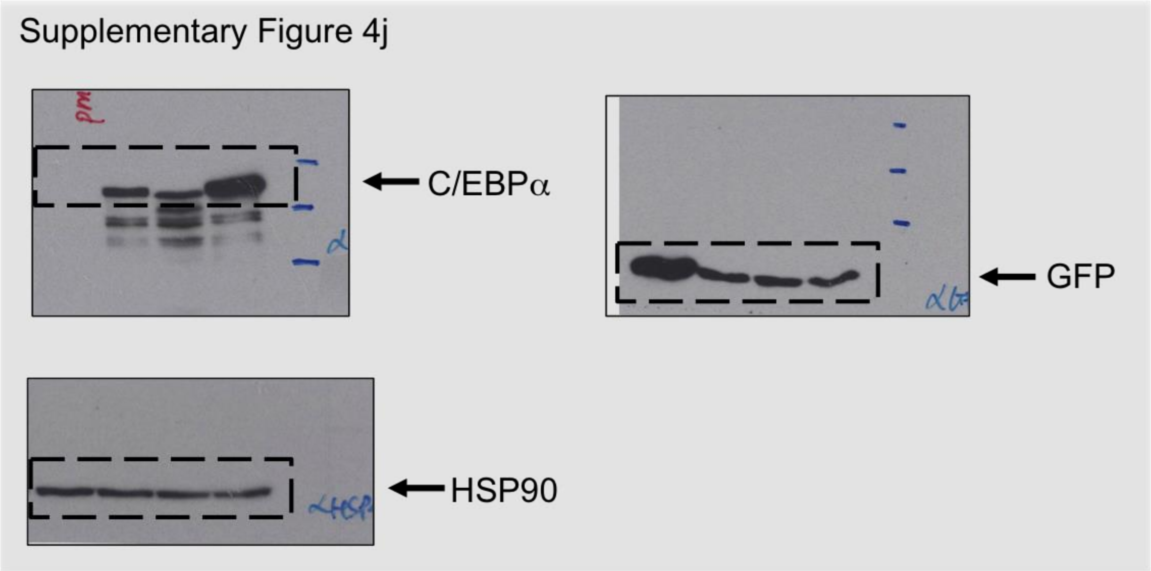

Supplementary Figure 11. Full scans of Western blot shown in Supplementary Figure 4.

Rectangles delimit cropped areas used in the indicated panels in Supplementary Figure 4

Supplementary Figure 12

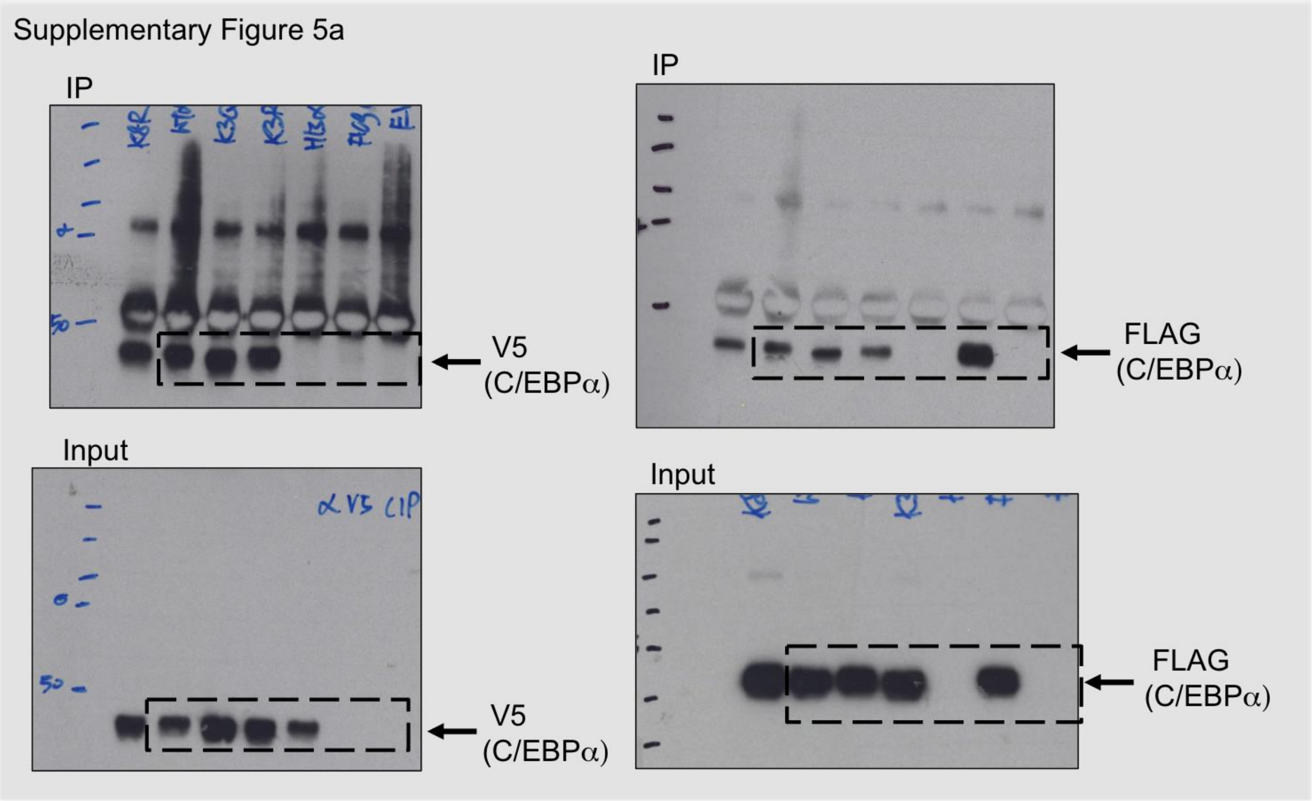

Supplementary Figure 12 (cont.)

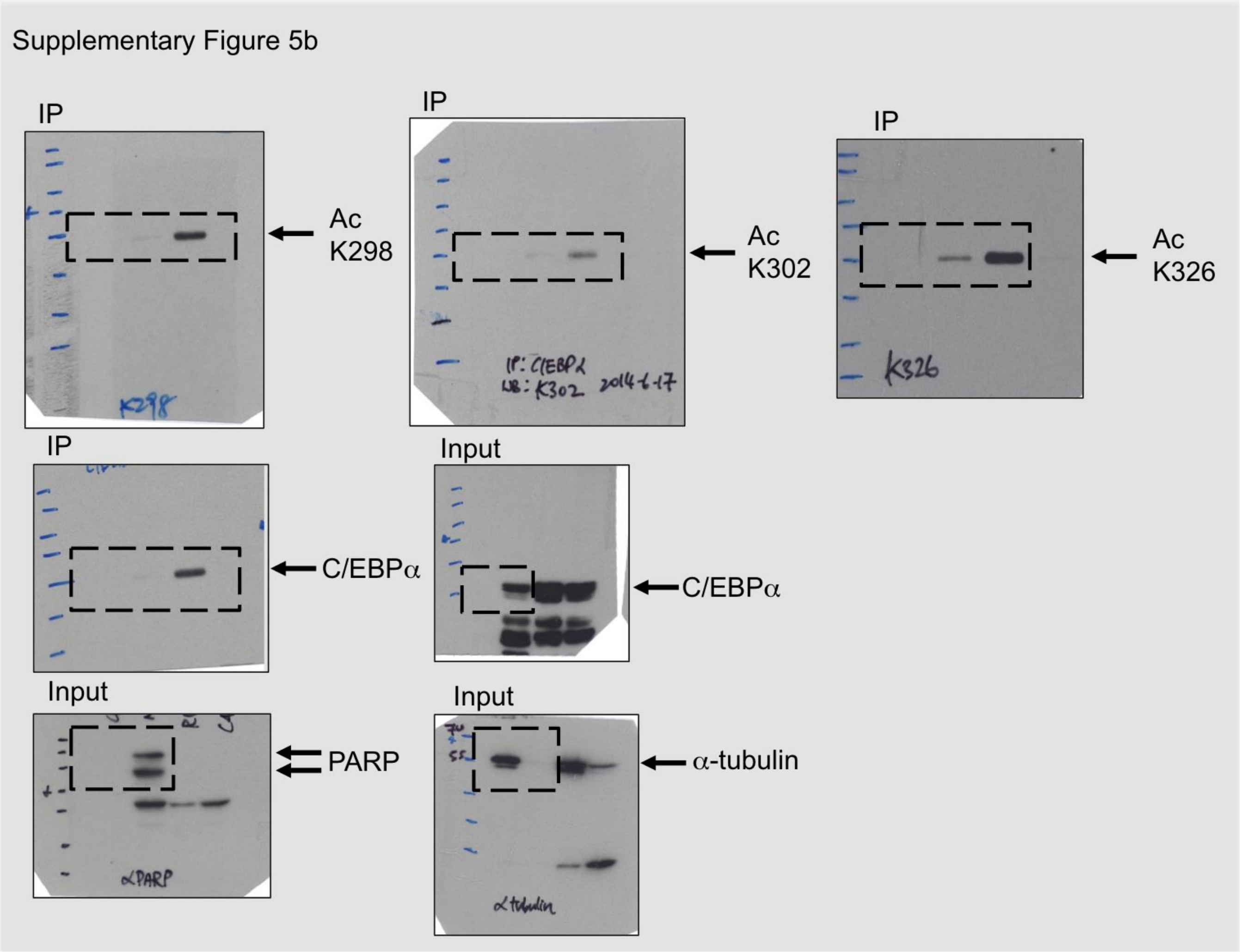

Supplementary Figure 12 (cont.)

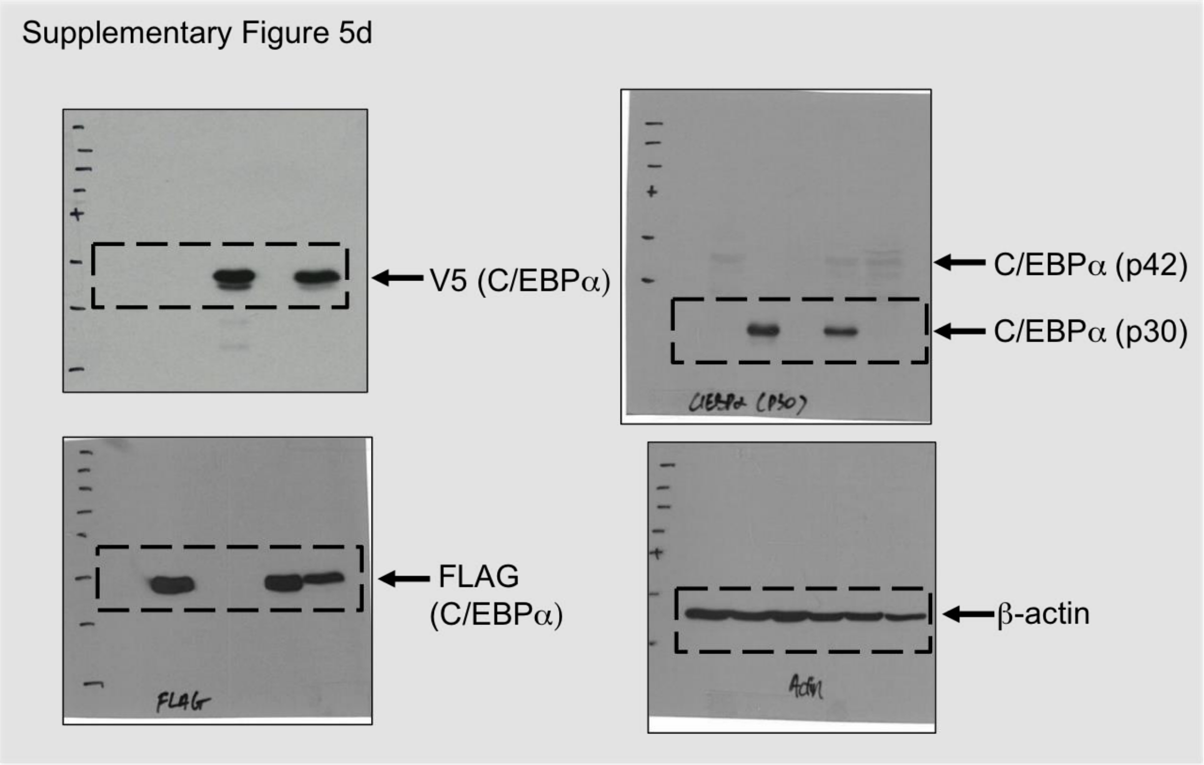

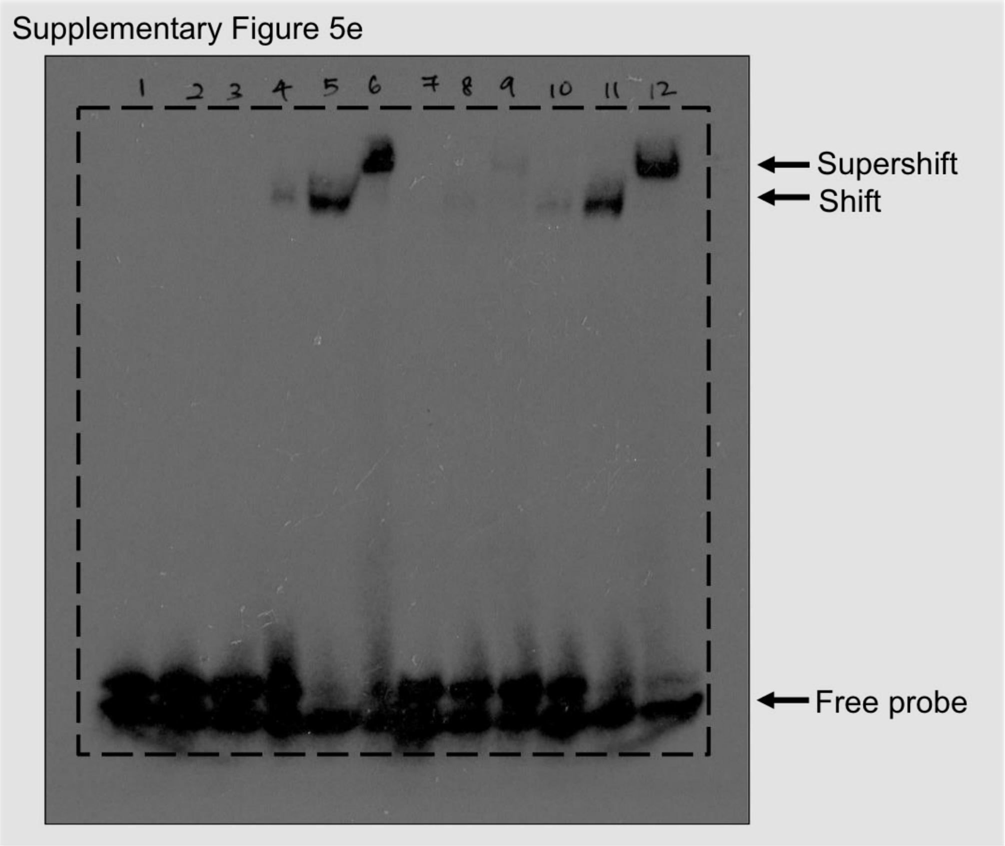

Supplementary Figure 12 (cont.)

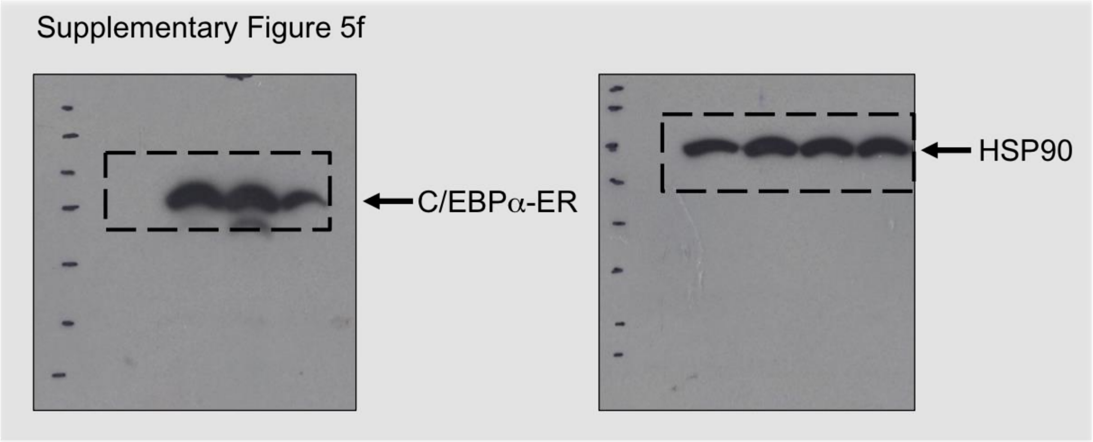

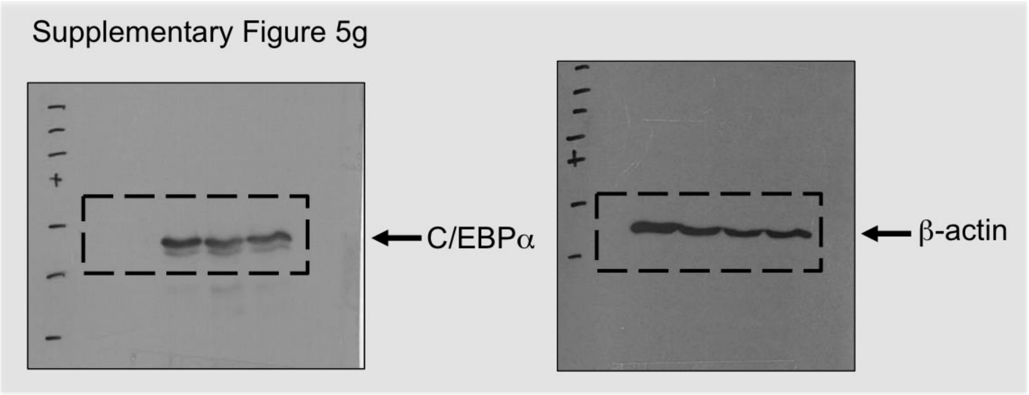

Supplementary Figure 12. Full scans of Western blot and EMSA data shown in Supplementary Figure 5.

Rectangles delimit cropped areas used in the indicated panels in Supplementary Figure 5.

Supplementary Table 1. Peptide sequences used for *in vitro* acetylation assay

	Amino acid position (N→C)	Peptide sequence
P1	81-92	LFQHSRQQEKAK
P2	154-165	ALRPLVIKQEPR
P3	162-173	QEPREEDEAKQL
P4	253-264	LKGLGAAHPDLR
P5	266-276	SGGSGAGKAKK
P6	277-288	SVDKNSNEYRVR
P7	294-306	IAVRKSRDKAKQR
P8	303-315	AKQRNVETQQKVL
P9	323-333	RLRKRVEQLSR
P10	343-354	RQLPESSLVKAM

Peptide sequences used for *in vitro* acetylation assay in Figure 1f. Highlighted in red are the putative acetylated lysine residues.

Supplementary Table 2. Summary of leukemia status of patient samples

AML sample #	Leukemic subtype	Cytogenetics	C/EBPα
2	M2	Normal	238_239insG, 929_930insTCT
3	AML-MDS	Complex	Negative
4	AML-MDS	Normal	Negative
5	M4	Normal	Negative
6	M1	Normal	Negative
8	M5	Trisomy 8	Negative

Leukemia status of patient samples used in Figure 2b and Supplementary Figure 2b

Supplementary Table 3. Hydrogen bonds between CEBPA and DNA

Gene	RES298--A^{-5}	RES302--T^{-4}	Total
WT-DNA	185	219	404
K2Q-DNA	53	151	204
K2Ac_a-DNA	23	163	186
K2Ac_b-DNA	6	114	120

Number of hydrogen bonds formed between protein and DNA key residues during 3 ns of MD simulation. RES = LYS, GLN or ACK.

Supplementary Table 4. Electrostatic interactions between CEBPA and DNA

Gene	Average	s.d.	s.e.m.
WT-DNA	-282.7	43.2	2.5
K2Q-DNA	-23.5	14.9	0.9
K2Ac_a-DNA	-16.9	9.3	0.5
K2Ac_b-DNA	-49.9	11.3	0.7

Electrostatic interaction [in kcal mol^{-1}] between protein residues 298 and K302 and DNA residues T^{-4}, A^{-5} and T^{-6} based on the 3 ns MD trajectories.
s.d.= standard deviation; s.e.m.= standard error of the mean.

Supplementary Table 5. Primary and secondary antibodies

Antigen	Company	Clone	Catalog #	Application /Dilution
C/EBPα	Santa Cruz	14AA	sc-61	IP 2 μg/WB 1:1000/EMSA 1 μg
C/EBPα	Santa Cruz	N-19	sc-9315	WB 1:1000
GCN5	Santa Cruz	H-75	sc-20698	WB 1:1000
ERα	Santa Cruz	HC-20	sc-543	WB 1:1000/ ChIP 2 μg
β-actin	Santa Cruz	C4	sc-47778	WB 1:5000
HA probe	Santa Cruz	F-7	sc-7392	WB 1:2000
GAPDH	Santa Cruz	L-18	sc-48167	WB 1:2000
C/EBPα	Cell Signalling Technology	D56F10	#8178	WB 1:2000
GFP	Cell Signalling Technology	D5.1	#2956	WB 1:2000
FLAG	Sigma	M2	A8592	WB 1:10000
α-tubulin	Sigma	B-5-1-2	T5168	WB 1:5000
pan-acetyl-lysine	Miilipore (Upstate)	4G12	05-515	WB 1:100
HSP90 antibody	Abcam	S88	ab1429	WB 1:2000
secondary anti-rabbit IgG	Amersham	-	NA934	WB 1:2000
secondary anti-mouse IgG	Santa Cruz	-	sc-2005	WB 1:2000
Clean-blot IP detection HRP-conjugated secondary antibody	Thermo Scientific	-	21232	WB 1:1000

Supplementary Table 6. Primary antibodies for FACS

Antigen	Specificity	Clone	Dilution
CD11b/Mac-1	human	ICRF44	1:50
Mac-1	mouse	M1/70	1:200
Gr-1	mouse	RB6-8C5	1:200
c-kit	mouse	2B8	1:100
Sca-1	mouse	D7	1:100
CD3	mouse	145-2C11	1:200
CD8	mouse	53-6.7	1:200
B220	mouse	RA3-6B2	1:100
CD19	mouse	1D3	1:100
Ter119	mouse	TER-119	1:100

Antibodies were from BD Pharmingen, Biolegend or eBioscience.

Supplementary Table 7. Human ChIP primers

Gene	Forward sequence	Reverse sequence	Product size (bp)
G-CSFR promoter	ATTCCCCAGCCCTTTAAGAC	CTGCAGTCCAGCTTCTCTCC	218
G-CSFR exonic	GGGAGTCCCATAACAGCTCA	AGTGGAGTCACAGCGGAGAT	194
involucrin promoter	GCCGTGCTTTGGAGTTCTTA	CCTCTGCTGCTGCCACTT	98
